# EBV-associated diseases: Current therapeutics and emerging technologies

**DOI:** 10.3389/fimmu.2022.1059133

**Published:** 2022-10-27

**Authors:** Srishti Chakravorty, Behdad Afzali, Majid Kazemian

**Affiliations:** ^1^ Department of Biochemistry, Purdue University, West Lafayette, IN, United States; ^2^ Immunoregulation Section, Kidney Diseases Branch, National Institute of Diabetes and Digestive and Kidney Diseases (NIDDK), National Institutes of Health (NIH), Bethesda, MD, United States; ^3^ Department of Computer Science, Purdue University, West Lafayette IN, United States

**Keywords:** high-throughput sequencing technologies, EBV therapeutics, EBV animal models, molecular mechanisms of EBV-host interactions, EBV-associated diseases and cancers, EBV vaccines

## Abstract

EBV is a prevalent virus, infecting >90% of the world’s population. This is an oncogenic virus that causes ~200,000 cancer-related deaths annually. It is, in addition, a significant contributor to the burden of autoimmune diseases. Thus, EBV represents a significant public health burden. Upon infection, EBV remains dormant in host cells for long periods of time. However, the presence or episodic reactivation of the virus increases the risk of transforming healthy cells to malignant cells that routinely escape host immune surveillance or of producing pathogenic autoantibodies. Cancers caused by EBV display distinct molecular behaviors compared to those of the same tissue type that are not caused by EBV, presenting opportunities for targeted treatments. Despite some encouraging results from exploration of vaccines, antiviral agents and immune- and cell-based treatments, the efficacy and safety of most therapeutics remain unclear. Here, we provide an up-to-date review focusing on underlying immune and environmental mechanisms, current therapeutics and vaccines, animal models and emerging technologies to study EBV-associated diseases that may help provide insights for the development of novel effective treatments.

## Introduction

Oncogenic viruses cause approximately 15-20% of all human cancers (1, 2). According to the International Agency for Research on Cancer (IARC), there are seven major human oncogenic viruses (3). These include DNA viruses, such as Epstein–Barr virus (EBV; also known as HHV4), Kaposi sarcoma-associated herpesvirus (KSHV; also known as HHV8), Hepatitis B virus (HBV), human papillomaviruses (HPV) and Merkel cell polyomavirus (MCPyV) and RNA viruses, such as human T-lymphotropic virus 1 (HTLV-1) and Hepatitis C virus (HCV). Despite many differences, these viruses have evolved common mechanisms to persist and replicate within host cells and facilitate escape of infected cells from the host’s immune surveillance. EBV and KSHV are two oncogenic viral agents of the γ-Herpesviridae subfamily that are known to modulate a plethora of biological processes in viral-associated cancers. The γ-Herpesviridae family is divided into two genera: *Lymphocrytoviridae* which includes EBV and *Rhadinoviridae* which includes KSHV. γ-herpesviruses encompass a broad range of pathogens in lower mammals ranging from murine herpesvirus-68, bovine herpesvirus 4 and equine herpesvirus 2 that closely resemble the rhadinovirus. Interestingly, to date, lymphocryptoviruses have been found only in primates and humans (4).

EBV was first discovered by Epstein, Achong, and Barr in 1964 who isolated this virus from the cells of a Burkitt lymphoma (BL) patient in Africa (5, 6). Since then, it has become evident that EBV infects ~95% of the world’s adult population. The typical transmission route is through bodily fluids, such as saliva, where the orally transmitted virions infect resting B and epithelial cells of the oral cavity. Primary infection is typically asymptomatic, although 35-50% of the human adolescent population develop infectious mononucleosis (IM) approximately 1 month after infection, and the virus persist throughout an individual’s life (7–10). After acute infection, a dormant state is established due to a strong, virus-specific T cell response (7). However, when the balance between the virus and host immune system is disrupted, EBV can drive malignant transformation of both lymphoid and epithelial origins, causing ~200,000 deaths annually (11–13).

As a herpesviruses, EBV can cause either latent or lytic infection. In epithelial cells, EBV typically undergoes lytic replication. In B cells, EBV usually establishes lifelong latency with rare sporadic reactivations. During latency only a few essential viral genes are expressed and production of virions are stalled (14–17). The switch from latent to lytic phase is governed by several factors (18, 19). While EBV-encoded products in both phases can play a role in transformation and tumorigenesis, the literature is more extensive on the oncogenic role of latent genes compared to lytic genes. However, it is challenging to target latent EBV using current immunotherapeutic strategies, specifically due to reduced antigen expression. As a result, patients with EBV^+^ or EBV^–^ tumors are typically subjected to similar treatment regimen. This underscores the need to investigate the complexity of EBV-host interactions to help the development of EBV-specific cancer therapies. In this review, we will first discuss the EBV lifecycle and different types of EBV-associated malignancies. We will then summarize the major underlying molecular mechanisms and therapeutic strategies for EBV^+^ cancers. Lastly, we will discuss some of the preclinical animal models and emerging technologies for investigating different aspects of host-pathogen interactions in EBV-associated malignancies.

## Epstein-Barr virus infection

### Lifecycle

**Figure 1:**
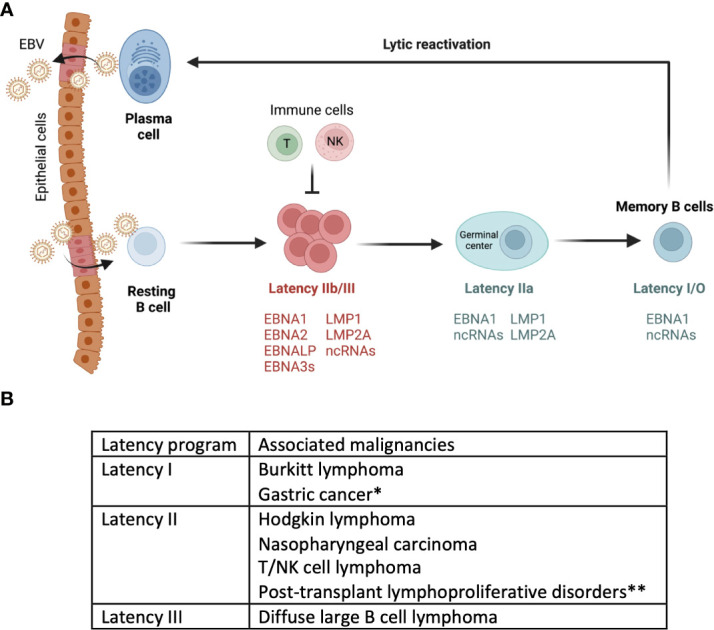
Model of EBV infection cycle. **(A)** Upon primary infection through saliva, EBV infects B cells. The figure depicts a model of EBV infection, where in EBV drives naïve B cells into latency III program. This activation leads to their differentiation into latency I/O memory B cells. This is often followed by spontaneous or induced reactivation of EBV within circulating memory B cells. This figure is adapted/modified from Guo et al (44). **(B)** Depending on the type of latency or lytic program, EBV infected cells are associated with different malignancies.*Gastric cancer cells also express genes that are associated with latency II programs. **Post-transplant lymphoproliferative disorders express some of the genes in latency III as well.

Epstein-Barr Virus (EBV) exhibits a biphasic lifecycle that includes latent and lytic (replicative) phases (20). Upon infection the virus typically establishes latency within the host cell (21). During latency, only a handful of latent genes that are necessary for the maintenance and persistence of the viral genome are expressed. EBV encodes eight latency genes whose expression in host cells and/or malignancies defines EBV latency programs (20). Based on which of the eight latent viral genes are expressed, viral infection is categorized into three main latency programs, latency III, II and I/0 (22). EBV-infected naïve B cells exhibit a latency III program, which allows for the proliferation and expansion of infected cells (23). Latency III genes include 6 EBV nuclear antigens (EBNA1, 2, 3A, 3B, 3C, LP), 2 latent membrane protein (LMP1 and LMP2), EBV-encoded small RNAs (EBERs), and EBV-encoded microRNAs (miRNAs) (24, 25). The cells in this latency program are highly immunogenic and can be rapidly eliminated by the host immune response, specifically by EBV-specific T cells (26). Latency II has a more restricted expression of EBV genes, namely EBNA1, LMP1, and LMP2A/B making them less immunogenic. Eventually, EBV sequentially shuts down the expression of all the latent genes except EBNA1 and a few EBV-encoded RNAs in latency I. Latency II can also be divided to IIa and IIb based on the expression of LMPs and EBNA2-3 (IIb is EBNA2-3^+^LMP^–^; IIa is EBNA2-3^–^LMP^+^). In most individuals, EBV persists quiescently within a subset of memory B cells (<0.005% B cells in the peripheral blood) without expressing any viral genes in latency 0 state, also referred to as a ‘true latency’ (24, 27, 28). Latent EBV genes are reported to promote tumorigenesis, inhibit apoptosis, and suppress recognition of infected cells by host immune cells (29). EBV-related malignancies are linked with different EBV latency programs. Lymphoproliferative disorders that are commonly associated with immunosuppression such as post-transplant lymphoproliferative diseases (PTLDs) and acquired immunodeficiency syndrome (AIDs) associated lymphomas exhibit latency III (29). Hodgkin lymphoma, T/NK cell lymphomas and nasopharyngeal carcinoma (NPC) exhibit latency II (30). Gastric carcinoma and Burkitt lymphoma exhibit latency I program (27, 31). So far, EBV in latency 0 has not been associated with any malignancies, presumably due to dormancy during this program (**Figure 1**).

The lytic phase is necessary for EBV progeny production and horizontal transmission of virus from host to host, so represents an integral aspect of viral pathogenesis (32). The switch from latent to lytic cycle can be either spontaneous or chemically induced. Some of the commonly used agents to induce lytic cycle include phorbol esters (PMA), sodium butyrate, calcium ionophores, DNA methyltransferase inhibitors (DNMTi), transforming growth factor-beta (TGF-β), doxorubicin and gemcitabine (because these are stress inducing chemotherapeutic drugs) and anti-IgG or anti-IgM as B-cell receptor stimulants (33–35). During lytic reactivation, the full repertoire of >80 viral genes is temporally regulated and expressed during three phases - immediate early (IE), early (E), and late (L). The first phase is primarily initiated by BZLF1 (ZEBRA) and BRLF1, the two key EBV immediate-early (IE) lytic transcription factors. Both genes function to promote their own and each other’s expression, as well as the expression of viral E genes, that code for proteins needed for viral replication (e.g., viral DNA polymerase). BZLF1 forms a homodimer *via* its basic leucine zipper motif and binds to BZLF1-responsive elements (ZRE) on DNA (36). The binding of BZLF1 to CpG methylated DNA leads to activation of several lytic viral genes that are silenced in latent cells by CpG methylation (37, 38). In addition, binding of BZLF1 to the origin of lytic replication (oriLyt) ZRE promotes lytic viral DNA synthesis (39). Similarly, BRLF1 binds to the BRLF1-responsive elements (RRE) on DNA and is reported to induce lytic replication *via* the PI3K and ERK signaling pathways (40, 41). Both BZLF1 and BRLFI are quintessential for EBV lytic replication since knocking out these genes blocks the latent to lytic switch. In addition, overexpression of BZLF1 and BRLF1 in latently infected cells can induce EBV lytic reactivation (42). This lytic induction leads to a cascade of viral gene expression, which promote viral DNA replication and virion production. Following viral replication, late viral genes code for structural proteins, such as gp350/220 encoded by the *BLLF1* gene are expressed (32). Interestingly, during lytic DNA replication in γ-herpesviruses, continuous DNA synthesis is needed for the transcription of late lytic viral genes but not for early lytic genes (20, 43). The virions can disseminate viral particles within host cells and among hosts. EBV replicates in latency I, II and III *via* proliferation of activated B cells. Interestingly, lytic replication can only be efficiently induced from latency I/0, and after extensive methylation of the viral genome. This is because BZLF1 prefers binding to methylated CpG sequences to initiate infectious particle production (38, 42).

### Genome organization and DNA methylation

The EBV genome is packaged similarly to that of host cells, that is to say into nucleosomes, except loci that harbor the origin of plasmid replication (OriP) (44, 45). Nucleosome folding along with several histone modifications at the promoter of lytic genes keep them transcriptionally silent during latent infection. For example, the recruitment of histone deacetylases (HDACs) at BZLF1 and EBNA2 Cp promoters maintain EBV latency (46) (47, 48) and, as expected, HDAC inhibitors, such as sodium butyrate, can activate the EBV lytic cycle (49, 50). Of note, EBV DNA within virions or soon after lytic replication is nucleosome free, potentially to allow its encapsulation into the nucleocapsid (44, 51).

DNA methylation is typically associated with transcriptional silencing (52, 53). The EBV genome is known to be hypermethylated in the latent form and in virions (54). DNA methylation plays a critical role in transcriptional regulation of LMP1 and EBNA2 and thus contributes to the transition among latency programs (55). Consistently, inhibitors of DNA methylation, such as 5-azacytidine can reactivate latently infected lymphoblastoid cells (LCLs) (56). However, since the OriP region is required for EBV transcriptional regulation, it is typically depleted of DNA methylation.

### Entry into host cells

EBV typically exhibits dual tropism with the capacity to actively infect and replicate both in epithelial and B-cells. Sometimes EBV can also infect other targets such as T lymphocytes and natural killer (NK) cells (7, 57). The entry of EBV into target cells is facilitated by its envelope glycoproteins (gp). B-cell entry requires glycoproteins gp350, gH, gL, gB and gp42, whereas epithelial cell entry needs BMFR2, gH, gL and gB (58–61). In epithelial cells, EBV is more likely to be transferred from EBV-positive B lymphocytes that cause lytic infection (62). T-cell and NK-cell entry seem to also require gp350 and gp42, respectively (63, 64).

The EBV virion has a diameter of 150–170 nm, consists of a linear, ~172 kbp double stranded DNA that codes for more than 85 protein coding genes (65, 66). However, the exact function of 30-40% of these genes remains unknown (67). The EBV genome also has several tandem repeat regions that serve various functions (20, 68). The entire genome is enclosed within an icosahedral capsid surrounded by a layer of tegument proteins and a lipid envelope that is made up of several unique glycoproteins. EBV can enter human B cells *via* a high-affinity interaction between viral gp350 and host complement receptor type 2 (CR2) protein. HLA class II can act as a co-receptor (15, 69). These protein-protein interactions stimulate endocytosis of the virus into non-clathrin coated vesicles of B cells. The virus also infects epithelial cells as well as T- or NK-lineage cells albeit at a lower frequency. Ephrin Receptor A2 (EphA2) was recently identified as the entry receptor for EBV in epithelial cells. This protein interacts with EBV glycoproteins gH/gL and gB (70). Although less is known about the mechanisms of EBV entry into other cells, CR2 has been identified as the entry receptor for T lymphocytes (64) but is apparently not essential for entry into NK cells (71). HLA class II also plays a role for entry into NK cells but its role for T cells remains less clear (63).

Upon entering B cells, the viral genome typically persists in the nucleus as a circular episome, expressing a subset of genes that promote survival of the infected host cell (58, 59). Typically, after initial infection, the EBV genome rapidly circularizes either before or at the same time as the initial phase of viral mRNA synthesis (21, 72–74). After B cell infection, EBV initiates an often asymptomatic, lifelong latency program in a few cells with extremely low viral activity. During this stage, the EBV episome is replicated by the host cell DNA polymerase primed on the EBV origin of plasmid replication (OriP) (75, 76).

### Variants and risk factors

EBV was originally divided into two major sub-variants, type 1 and type 2, based on the sequence of two EBV-encoded genes - EBNA2 and EBNA3 (77, 78). Type 1 is prevalent globally (e.g., B95-8, GD1, and Akata strains) while type 2 (e.g., AG876 and P3HR-1 strains) is endemic to sub-Saharan Africa (79). Currently, more that 71 distinct EBV strains have been identified. EBV variants have different replicative properties and individuals may become infected with two or more strains. With the advent of high-throughput sequencing technologies, it is now possible to sequence EBV genomes from clinical specimens of diverse populations with different malignancies. The first sequenced genome was of the prototypical EBV B95-8 strain. This strain harbored a 12-kb deletion in its genome. It was not until 2014 that this defect was noted and EBV from Raji strains was recovered to get the final complete sequence of wild-type EBV (EBV-wt,26 GenBank accession no. NC_007605.1) (65). This is now the gold standard reference sequence for many research groups in the field. It has been reported that certain EBV strains have more oncogenic potential than others. For instance, the NPC derived EBV strain, M81, spontaneously replicates at an unusually high rate in B cells and has an extremely high propensity to infect epithelial cells (80). This ‘super infectious’ property is attributed to a single nucleotide polymorphism (SNP) in the *BZLF1* promoter region that confers binding by host cellular transcription factors, notably NFATc1 (81). Increasing studies are now investigating the heterogeneity of EBV latent and lytic genes among the different EBV strains in order to identify high-risk EBV strains (79). Doing so will potentially help identify high-risk infected individuals and facilitate development of effective EBV vaccines and anti-EBV T-cell therapies.

## EBV encoded latent gene products and oncogenesis

EBV latent proteins are generally considered key drivers of tumorigenesis in EBV-associated cancers and thus it is important to understand their functions in establishment of persistent infection and cellular transformation. In this section we will briefly describe the function of EBV-encoded latent gene products and their role in transformation and oncogenesis in EBV-associated malignancies.

### Epstein–Barr virus nuclear antigen 1

EBNA1 is a transcription factor that is essential for EBV episomal maintenance and replication (82, 83). Consistently, EBV variants that harbor EBNA1 deletion do not have the capacity to establish episomal latent infection (84). The DNA binding domain of EBNA1 is necessary but not sufficient for EBV replication and requires the N-terminal region (85). Since EBNA1 lacks enzymatic activity, it primarily recruits host cellular factors to replicate EBV episomes and to govern mitotic segregation (86). Of note, EBNA1 can also function as a transcriptional repressor and can downregulate its own transcription in an autoregulatory loop (87–89). In terms of oncogenic potential, EBNA1 is involved in progression of carcinogenesis. Specifically, EBNA1 deletion significantly decreases immortalization efficiency, while its overexpression inhibits apoptosis (90, 91). EBNA1 modulates several cellular signaling pathways that provide survival advantage to infected cells (92). While EBNA1 is reported to enhance phosphorylation of STAT1 in one gastric cancer cell line and two nasopharyngeal cancer cell lines, it inhibits anti-tumor TGF-β1 and NF-κB pathways, promoting tumorigenesis (93, 94). EBNA1 also upregulates several proteins involved in metastasis and oxidative stress in EBV^+^ NPC cells (95). In addition, EBNA1 induces loss of promyelocytic leukemia (PML) nuclear bodies and subsequently abrogates PML functions, such as p53 activation and apoptosis, resulting in increased survival of gastric cancer cells (96). The fact that EBNA1 is the only EBV protein that is consistently expressed in all latency types, and therefore in all EBV-associated tumors, makes it a key target for EBV specific therapies. Consistently, pharmacological inhibition of EBNA1 using a small-molecule inhibitor VK-1727 has been tested in various *in vivo* xenograft mouse models for specific EBV^+^ cancers. These studies have demonstrated that inhibition of EBNA1 can selectively suppress EBV^+^ tumor cell proliferation (97).

### Epstein–Barr virus nuclear antigen 2

EBNA2 is a transcriptional activator of both cellular (e.g., *CD21*, *CD23* and c-*MYC*) and viral genes (e.g., LMP1 and LMP2) (98, 99). EBNA2 plays a crucial role in transcriptional reprogramming of B cells to facilitate growth and survival (100, 101). However, unlike EBNA1, it cannot bind to DNA directly and requires host transcription factors, such as the Notch pathway DNA-binding factor RBP-Jκ and PU.1 to regulate gene transcription (102). In terms of oncogenic potential, EBNA2 plays a crucial role in the transformation process and functionally mimics Notch (103, 104). Consistently, P3HR-1, a variant EBV strain in which EBNA2 and the last two exons of EBNA-LP are deleted, does not transform B cells *in vitro*. EBNA-LP, a transcriptional co-activator of EBNA2, is also an important EBV oncoprotein that drives B cell transformation and functions by up-regulating the expression of EBNA2 targets (105). In addition, EBNA2 activates *MYC* enhancers *via* long-range interactions. MYC can both increase proliferation and sensitize cells to apoptosis. However, it is also a known proto-oncogene, so unsurprisingly, EBNA2-mediated *MYC* activation seems to promote lymphomagenesis in Burkitt lymphoma (106).

### Epstein–Barr virus nuclear antigen 3 family proteins

The EBNA3 protein family members are stable, tightly regulated and consist of EBNA3A, EBNA3B and EBNA3C (107). EBNA3 proteins are well studied classes of transcriptional regulators known to regulate both EBV (e.g., *LMP1*) and host gene (e.g., *CD21*) expression and, depending on context, can function as activators or repressors of gene expression (108–110). All EBNA3 proteins play a role in transformation and prolonged persistence of EBV in infected B cells. EBNA3 transcripts are generated from the Cp latency promoter and are reported to be only expressed in B cells as a part of the latency III program (111). Like EBNA2, EBNA3 proteins also do not directly bind to DNA but are, rather, recruited by cellular DNA binding factors, such as RBP-Jκ (112, 113). RBP-Jκ tethers EBNA3s to chromatin, but binding of EBNA2 and EBNA3 to RBP-Jκ are mutually exclusive (113, 114). In terms of their oncogenic potential, the EBNA3 family of proteins have antagonistic functions but cooperate in a complex to facilitate EBV persistence, as well as to promote oncogenic transformation. EBNA3A and EBNA3C are considered oncogenes since they are also involved in B cell transformation. However, in the absence of EBNA3A and EBNA3C, EBV can latently persist in humanized mice (115). Despite the sequence and structural similarity and reports about their functional cooperativity, EBNA3 proteins are often have opposing functions. For example, EBNA3B is dispensable for B cell transformation but inhibits EBNA3A- and EBNA3C-mediated oncogenic functions *in vivo*. In addition, like EBNA2, EBNA3 has long-range interactions with enhancers and super-enhancer elements that drive oncogenesis (116).

### Latent membrane protein 1

EBV encoded LMP1 is an essential membrane protein that has an N-terminal cytoplasmic tail, six transmembrane domains and a C-terminal cytoplasmic region that is divided into two C-terminal activation regions 1 and 2 (CTAR1 and CTAR2). These regions are required for tethering LMP1 to the plasma membrane and its signaling activity (117).. LMP1 mimics cellular CD40 receptor, a member of the (TNFR) superfamily and can drive growth and differentiation of B cells by substituting CD40 functions *in vivo* (118). LMP1 signaling is primarily mediated by the ability of host TNFR-associated factors (TRAFs) or death domain protein TRADD to interact with CTAR1 or CTAR2 to facilitate activation of upstream regulators of several signaling pathways (119, 120). In terms of oncogenic potential, LMP1 is a well-documented EBV oncogene and is essential for transformation of B cells *in vitro*. LMP1 acts as a constitutively active CD40 receptor and thus can activate target signaling pathways (e.g. the NF-kB pathway) independent of ligand engagement (121–123). These includes pro-tumorigenic functions, such as increase in cell proliferation, cytokine production (IL-6, IL-8), apoptotic resistance (by upregulating the expression of anti-apoptotic protein (Bcl-2, A20)), immune modulation, anchorage-independent growth, metabolism, angiogenesis, metastasis and invasion, all of which are known to contribute to EBV-mediated pathogenesis (124–126).

### Latent membrane protein 2

The LMP2 gene encodes two dominant isoforms, LMP2A and LMP2B. LMP2B is the smaller isoform that structurally lacks a short cytoplasmic N-terminal domain that harbors an essential survival signal known as immunoreceptor tyrosine-based activation motif (ITAM) (75, 127). Interestingly, B cell receptor (BCR) also has an ITAM motif which, upon phosphorylation, recruits and activates the *Src* family and *Syk* protein tyrosine kinases (PTKs) and promotes B cell proliferation and differentiation. However, the association of these PTKs with the phosphorylated ITAM of LMP2A negatively regulates PTK activity (127), thereby inhibiting BCR-driven calcium flux, tyrosine phosphorylation and BZLF1 induction in LCLs (128). In terms of oncogenic potential, LMP2A and LMP2B seems to be dispensable for *in vitro* B-cell transformation (129). Indeed, LMP2 has anti-oncogenic potential (130, 131).

### Other EBV-encoded latent gene products

In addition to the latent proteins mentioned above, there are a few other EBV-encoded latent gene products including EBERs, BARTs and EBV microRNAs. Two EBERs (EBER 1 and 2) are small non-coding RNAs that are expressed during all latency programs. In terms of oncogenic potential, EBERs do not seem to be essential because EBER-deleted EBV strain can similarly transform primary B-lymphocytes (132). However, they can affect cellular processes by enhancing anti-inflammatory cytokine IL-10 production *via* RIG-I/IRF3 activation (133). In certain B cell lymphomas with restricted type 1 latency, EBER2 and EBNA1 can induce expression of cytokines (e.g., IL6) or cytokine receptors (e.g., CD25) to promote B cell survival (134). The BARTs encode BARF0, RK-BARF0, A73 and RPMS1. The function of BART proteins encoded by corresponding ORFs needs further examinations. The 44 EBV microRNAs are arranged either adjacent to the BHRF1 gene or within the BART introns. These microRNAs are associated with different EBV latency programs (135, 136) and are differently induced during the lytic cycle (137). However, the significance and function of most remain unclear.

## EBV-associated diseases

EBV is associated with a wide variety of diseases and malignancies. Infectious mononucleosis (IM) is an extremely common, self-limiting, and acute disease associated with primary EBV infection. It is characterized by lymphadenopathy, transient fever and hepatosplenomegaly that usually resolves in time. Chronic active EBV infection (CAEBV), although rare, is a severe and fatal condition characterized by unusually high EBV DNA load (10^3^–10^7^ copies/mL) (138), which is now considered to be one of the EBV^+^ T or NK cell lymphoproliferative diseases and can lead to two lethal conditions: hemophagocytic lymphohistiocytosis and chemotherapy-resistant lymphoma (139). Historically CAEBV was partially managed using immunomodulatory agents such as interferon-α (IFN-α) and IL-2 (140), but JAK/STAT inhibitors are now a standard component of treatment (141).

EBV is also a major risk factor for immunocompromised patients. In HIV patients the lack of efficient and EBV-specific T cell responses significantly increases the risk of developing EBV-associated lymphoma (142, 143). Oral hairy leukoplakia (OHL) is a hyperproliferative disorder observed in immunocompromised patients that is triggered by EBV lytic state (144–147). Post-transplant lymphoproliferative disorder (PTLD) represents severe, life-threatening uncontrolled B cell proliferation post-organ or bone-marrow transplantation, which in majority cases is associated with EBV reactivation and replication (148). EBV infection is typically associated with cases of early onset (within one year of transplantation) compared to late-onset PTLDs (149) and higher EBV viral loads increase the risk of PTLD (150). One of the main reasons for EBV reactivation during transplantation is the use of immunosuppressants to prevent transplant rejection. However, these immunosuppressants inhibit all T cells, including EBV-specific ones, providing an opportunistic means for EBV to escape from immune surveillance (151). Consistently, pre-emptive treatment with inhibitors of EBV DNA replication can reduce the incidence of PTLD (152).

EBV infection has also been implicated in the development of autoimmune diseases, such as multiple sclerosis (MS) (153). MS is characterized by autoreactive B cells in the cerebrospinal fluid (CSF) that attack the myelin sheath of the central nervous system (CNS). Recently, a large-cohort study on 62 million serum samples taken from over 10 million US military personnel provided compelling evidence suggesting a necessary but not sufficient role of EBV infection towards the development and progression of MS (154). Pathologically, another recent study showed that EBNA1 mimics the CNS protein glial cell adhesion molecule (GlialCAM), which is expressed by myelin sheath-forming cells. Antibodies against a particular region of EBNA1 highly cross-react with GlialCAM in MS patients, potentially resulting in “off-target” autoimmune attack against the myelin sheath in CNS of patients with MS (155).

The ability of EBV to immortalize B cells is testament to its tumorigenic potential (156, 157). Indeed ~1-2% of all human tumors are attributed to EBV, equating to ~300,000 new cases worldwide in 2020 (158–160). EBV infects both genders, however, EBV-associated malignancies are slightly more prevalent in males compared to females (161). EBV infection is associated with various lymphomas, including Burkitt’s lymphoma (BL), Hodgkin lymphoma (HL), diffuse large B cell lymphoma (DLBCL), NK/T cell lymphoma and primary effusion lymphoma (6, 162), as well as epithelial malignancies, such as NPC and gastric carcinoma (GC). Below, we will discuss some EBV-associated malignancies in greater detail.

### Burkitt’s lymphoma

BL is a highly aggressive B cell non-Hodgkin neoplasm first reported in Africa by Denis Burkitt (163). The WHO classification describes three clinical variants of BL: endemic (eBL), sporadic (sBL), and immunodeficiency-related (usually HIV-related) (164). While around 95% of eBL are EBV^+^, only 15% of sBL and 40% of immunodeficiency-related BLs are associated with EBV (67). Despite primarily having the type I latency programs, some other EBV genes (e.g., EBNA2) are sporadically detected (165). Although the role of most EBV genes in BL pathogenesis remains unclear, it has been shown that EBNA1 can inhibit apoptosis in BL cell lines by interacting with host proteins, such as p53-regulator USP7 and an anti-apoptotic protein survivin (166).

The eBL variant is common in malaria endemic regions and is commonly characterized by the presence of large tumors in the head and abdominal cavity. EBV is detected in nearly all the cells of eBL tumors, however the exact underlying mechanism has not yet been fully elucidated (167). Fortunately, BL tumors typically respond to intensive chemotherapy and are often curable when diagnosed early but it still remains a fatal disease in much of the affected sub-Saharan African population (168). This is attributed to several factors, such as diagnostic delay, inadequate healthcare, poverty, and malnutrition (169, 170). Outside of malaria-endemic regions, the occurrence of BL is about 10-fold lower mostly constitutes the sBL variant, which is concurrent with a lower EBV prevalence (10-30%) (171). Clinically, the majority of sBL cases present as tumors in the abdominal and thoracic cavities. Despite a poor prognosis, current ongoing clinical trials using a modified chemotherapeutic approach are showing some promise (NCI 9177 trial). Nonetheless, more specific and less toxic treatment options are needed.

The best known molecular feature of BL is the translocation of the proto-oncogene MYC to an enhancer locus next to the immunoglobulin heavy chain gene, causing constitutive expression of MYC (172, 173). However, in addition to enhanced MYC activity, the development of BL requires additional genetic or epigenetic aberrations (174). Over the years, extensive genomic and transcriptomic characterization of BL cases have identified genes that are recurrently mutated (e.g., *BCR*, *TCF3*, *ID3*, *CCND3*, *ARID1A* and *SMARCA4*) (175, 176). For instance, it has been experimentally demonstrated that mutations in *ID3* promote proliferation and cell cycle progression (177). Genes in the ID3-TCF3-CCND3 pathway are frequently mutated in MYC-rearranged eBLs and may represent one of the major underlying causes of BL (178). Nevertheless, the mutational and transcriptional landscape of EBV^+^ BLs is quite distinct from EBV^–^ BLs and is primarily attributed to the presence of EBV. Recent studies have explored the spectrum of aberrations in EBV^+^ BLs and the complex interplay between specific viral-host transcriptional programs (179, 180). For example, the frequency of *MYC*, *ID3*, *TCF3* and *p53* somatic mutations is lower in sBLs, while the frequency of mutation in *ARID1A*, *RHOA* and *CCNF* are higher in eBLs (179). Interestingly, it has been previously reported that LMP2A enhances MYC driven lymphomagenesis through activation of the PI3K-pathway (181–183), suggesting that activation of PI3K by LMP2A might be an alternative and/or convergent mechanism to the one driven by *TCF3*/*ID3* mutations. In addition to LMP2A, a recent study described the role of LMP1 in MYC-induced lymphomagenesis in a subset of BL cases (184). Further, comparative transcriptome analysis of eBL and sBL tumors have highlighted key mutational differences between the two types of BLs, with sBL having a significantly higher mutational burden, which correlates strongly with the EBV status rather than geographical distribution (185, 186). A recent study has found a genome-wide increase in aberrant somatic hypermutation (SHM) in EBV^+^ BLs, attributed to the higher expression of host activation-induced cytidine deaminase gene AICDA, which is also a target of EBNA3C (187). Overexpression of AICDA increases the likelihood of DNA breaks and *MYC* translocations as well as pathogenic mutations (188). Other factors, such as co-infection, seem to also contribute to BL pathogenesis. For example, *Plasmodium falciparum* induces DNA damage, which can turn EBV-infected B cells into eBL. Likewise, impaired immune surveillance in HIV-infected patients can induce EBV-associated BLs (179, 189, 190).

### Hodgkin lymphoma

HL is a lymphoid neoplasm that originates from B cell. The two major forms of HL are the classical type (cHL) and the nodular lymphocyte predominant type (NLPHL), the latter being considered as an EBV^–^ malignancy. One of the main features of cHL is the presence of large multinucleated cells known as Hodgkin and Reed-Sternberg (HRS) cells (191). Although derived from B cells, HRS cells lack the normal B cell phenotype which is attributed to functional aberrations in key B-cell associated TFs, such as PAX5, EBF1, TCF3/E2A and NF-κB (192, 193). In addition, the HRS cells co-express various hematopoietic cell markers and have anomalous activation of several signaling pathways (e.g., NF-κB and JAK/STAT), attributed to the frequent mutations of key TFs and/or cellular interactions within the tumor microenvironment (TME) (194). Globally, nearly 50% of cHL cases are EBV^+^ but the EBV prevalence varies with geography. For instance, about 30-40% of cHL cases in North America and Europe are EBV^+^, while in Africa, Asia, and Latin America, all cases are EBV^+^ (192). EBV^+^ cHLs are typically characterized by a massive immune cell infiltration (195). Although the exact mechanistic role of EBV in cHL pathogenesis is unclear, the presence of EBV throughout disease progression underscores its role in maintaining the tumor phenotype (196). EBV^+^ cHLs exhibit type II latency program, maintaining high levels of LMP1 and LMP2A proteins in all HRS cells (191, 197). LMP1 and LMP2A can both contribute to the pathogenesis of cHLs by mimicking cellular receptors, namely CD40R and BCR, that are essential for cell survival and expansion (99, 198–204).

### Nasopharyngeal cancer

NPC is a unique and complex form of a head and neck epithelial cancer. While the disease prevalence is extremely low in the Western world, it is endemic in Asia, Southeast Asia, North Africa, Greenland, Alaska, and the Middle East, affecting around 30 per 100,000 individuals. The distinct geographical distribution pattern of NPC cases worldwide suggests both environmental (e.g., consumption of preserved, salt-cured foods) and genetic factors (e.g., mutations in *HLA*, *TNFRSF19*, *CDKN2A/B*, and *TERT*) as its etiology (205–208). Nonetheless, EBV infection is reported to be a critical risk factor and plays an essential role in NPC progression (209–211). About 90% of malignant cells in NPC are either undifferentiated or poorly differentiated squamous epithelial cells that typically express several EBV latency type II gene products (212). These include EBER1/2, EBNA1, LMP1, LMP2, BARF1, and several other EBV-encoded non-coding transcripts. LMP1 is one of the key oncogenic drivers of NPC that is expressed in 20%–60% of NPCs and all pre-malignant or pre-invasive lesions, making it a prime therapeutic target (213).

NPCs harbor a high somatic mutation burden. A recent study identified more than 50 mutations per tumor in a panel of 111 micro-dissected EBV^+^ tumor samples. A whole exome sequencing study of NPCs identified a range of somatic mutations in key cellular genes and pathways including p53, HLA, NF-κB, MAPK, and P13K (214). Given that somatic mutations in NF-κB pathway were mutually exclusive to LMP1-overexpressing NPCs, the NF-κB pathway activation either by EBV or mutation seems to be vital for NPC pathogenesis (215). This is corroborated by another genome-wide analysis that reported that ~90% of the EBV^+^ NPCs have constitutive activation of NF-κB inflammatory pathways either due to somatic mutations or expression of EBV encoded LMP1 oncogene (216). Additionally, chromosome instability (CIN) is hallmark of NPCs. Early studies have linked EBV infection with genomic instability. Detailed differences in the genomic and epigenomic landscapes of EBV driven epithelial malignancies have been reviewed elsewhere (207).

### EBV-associated gastric cancer

Gastric cancer (GC) is one of the leading causes of cancer-related mortality (165). Nearly 10% of the 950,000 yearly new cases of GC cases are attributed to EBV infection. EBV^+^ GC usually mimics the histological features of lymphoepithelioma-like carcinoma, in which dense lymphocytic infiltrates (mainly CD8^+^ T cells) are present. EBV^+^ GC is, in fact, of the four molecular subtypes of GC, namely EBV^+^ GC, GC with microsatellite instability, genomically stable GC, and GC with chromosomal instability (217). EBV^+^ GC and GC with microsatellite instability are mutually exclusive. There are two common theories regarding the mechanism of occurrence of EBV^+^ GC. First, that EBV enters the digestive tract *via* the saliva and directly infects gastric epithelial cells. Second, EBV within B cells of the stomach is reactivated (through unknown mechanisms) and infects surrounding gastric epithelial cells (218). EBV^+^ GC cells exhibit latency I or intermediate latency I/II programs (219, 220). Consistently, EBNA1 and LMP2A are expressed in 100% and ~50% of EBV^+^ GC cases, respectively, but LMP1 is not expressed (221). EBV^+^ GCs have distinct genomic aberration, clinicopathological features, cellular gene methylation, and comparatively favorable prognosis compared to EBV^–^ GCs (217, 222–224). Unlike NPCs and EBV^–^ GCs, p53 mutations are rare in EBV^+^ GCs (225). This might also partially explain a comparatively favorable prognosis for EBV^+^ patients since it is known that mutations in p53 reduces sensitivity to chemotherapy and radiation (226). EBV can also extensively induce cellular DNA methylation, which could inhibit tumor suppressor genes (e.g. *p16* and *E cadherin*) and thus increase the risk of cancer formation (227). Recent studies report the increased expression of certain immune checkpoint proteins in EBV^+^ GC, such as PD-L1 and IDO-1 and their upstream regulators (180), which could explain their favorable response to immune checkpoint therapy (228). The pathogenic role of EBV, underlying molecular mechanisms and current treatment options for EBV^+^ GC have been further discussed elsewhere (218).

## Mechanisms underlying EBV induced diseases

**Figure 2:**
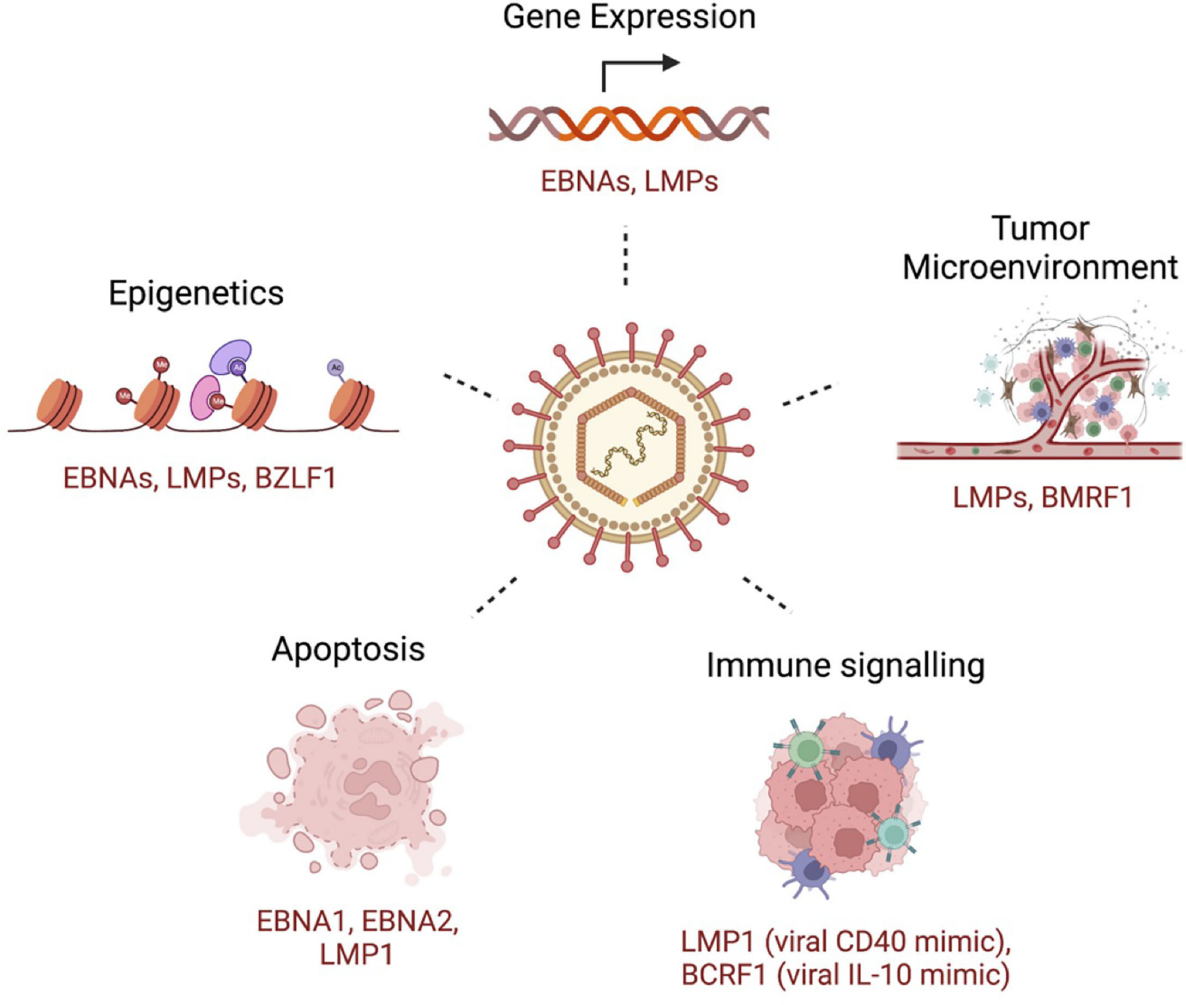
A few examples of key host cellular processes perturbed by EBV. Examples of EBV-encoded gene products that are related to the indicated mechanism are shown.

To establish infection and persistence, EBV employs different strategies to evade the host immune response and to compromise innate and adaptive immunity during its life cycle (229). Some of these mechanisms includes genetic and epigenetic alterations, inhibition of apoptosis, enhanced cell proliferation and inhibition of immune recognition of EBV-infected cells (**Figure 2**). Broadly, tumorigenesis can occur by i) enhancing antiapoptotic or reducing proapoptotic gene expression; ii) promoting cell growth and survival signaling pathways; and iii) shaping the tumor microenvironment for malignant cells to escape immune surveillance. In this section, we will briefly discuss some of these mechanisms, focusing on the role of the tumor microenvironment and immune escape mechanisms in EBV-induced malignancies.

### Tumor microenvironment

EBV is known to alter the cellularity and the properties of the tumor microenvironment (TME) thereby shaping an immunosuppressive environment. This involves inhibition of anti-tumor effector immune cells, such as NK cells and CD8^+^ T cells and recruitment and differentiation of immune-suppressive and/or anti-inflammatory cells, such as regulatory T cells (Tregs), dendritic cells (DCs), Th17 cells, M2-polarized tumor-associated macrophages (TAMs) and myeloid-derived suppressor cells (MDSCs) (230). In addition to the immune compartment, TME also includes stromal cells, soluble mediators such as chemokines and cytokines that can be modified by EBV. Together, these changes facilitate tumor growth by several mechanisms including promoting immune evasion. Extensive research has been done to understand the TME of both EBV^–^ and EBV^+^ malignancies (67). Studies on the TME are limited for certain EBV^+^ malignancies due to low incidence rates (e.g. NK/T cell lymphoma) or high heterogeneity among the sites of the disease and immune compartment (e.g. PTLD).

It is known that tumor cells can affect immune cell infiltration as well as drive the infiltrated immune cells towards a tolerogenic or exhausted state rendering them non-functional. Although highly variable, EBV^+^ carcinomas are generally characterized by high immune cell infiltration. This includes CD8^+^ T cells, CD4^+^ T cells (Th1, Th2, Treg cells, etc.) and CD163^+^ M2-TAMs (TAMs) (231). T cells are prevalent in EBV^+^ epithelial cancers. For example, EBV^+^ GCs attract high numbers of CD8^+^ T cells better known as cytotoxic T lymphocytes (CTLs) (232) and the CTL infiltration is positively correlated with EBV viral load (233). Despite a significant increase of CD8^+^ T cells within the TME of EBV^+^ NPCs, they exhibit an exhaustion signature and reduced cytotoxic activity (234, 235). In EBV^+^ NPC, LMP1-mediated glycolysis promotes MDSC expansion within TME leading to tumor-induced immunosuppression (236). Tregs and the CD8^+^ T cells are dominant in cHL. However, CD8^+^ T cells are primarily exhausted due to the high levels of PD-L1 expression (237). In contrast, higher numbers of M2-TAMs seems to be the most prominent in the TME of Burkitt lymphomas (238), which are also known to affect tumor progression *via* upregulation of immune checkpoints and expression of specific cytokines. Similarly, an increased frequency of CD57^+^ NK cells is reported in EBV^+^ GC, NPCs and cHL compared to their EBV^–^ counterparts (231). Unlike T cells, the role and the presence of B cells within the TME of EBV^+^ malignancies are mixed and warrant further investigation.

Altered expression profile of certain soluble mediators including cytokines within the TME have an important role in EBV-associated malignancies and often these alterations precede immune cell infiltration. For instance, EBV induces host CXCL9, CXCL10, and CCL20 in some EBV^+^ tumors, which in turn attract regulatory T cells into TME (239, 240). The cytokines often have pleiotropic effects. For instance, IL-10 is known to downregulate the expression of HLA class I and II antigens, induce Tregs (which in turn inhibit T-cell proliferation and IFN-γ secretion) and inhibit CD8^+^ T cell cytotoxic function resulting in an overall immune-suppressive microenvironment within the tumors (241–243). Other soluble mediators such as IL-1β, IL-4, IL-6, IL-8, and IL-13, IFN-γ, CXCL10 and CXCL12 are also frequently upregulated in EBV^+^ malignancies and implicated in disease progression (231). It is also known that EBV proteins such as LMP1 and EBNA1 can significantly promote an immunosuppressive microenvironment by promoting expression of chemokines and cytokines. Compared to their EBV^–^ counterparts, EBV+ GC cells have an overall higher involvement of Th1 and CD8^+^ T cells and produce more cytokines/chemokines including CCL20, CCL22, and CCL17 (244, 245). Other non-immune cells within the TME (e.g., cancer-associated fibroblasts) are also known to produce pro-inflammatory cytokines and have been reported to surround tumor cells in EBV^+^ solid tumors (246).

### Immune evasion

The human immune system has developed several strategies to combat invading pathogens. Central components of such strategies are the innate and adaptive immune responses. While innate immune responses are the first line of defense, they are often non-specific. In contrast, the adaptive immunity is more specific and long lasting and maintains specific memory of invading pathogens. Despite these host immune defense tools, EBV can establish latency within infected cells suggesting that the virus has developed mechanisms to escape, inhibit, or subvert host immune responses to ensure its own persistence. A typical mechanism of innate immune evasion in EBV infection is downregulation of pattern recognition receptors, such as toll like receptors (TLRs). Similarly a general mechanism of adaptive immune-evasion in EBV-associated malignancies is the overexpression of immune checkpoint proteins (e.g., PD-L1, IDO-1, CTLA-4, LAG-3, TIM-3, and VISTA), thus making them susceptible to treatment with immune checkpoint blocking immunotherapy (247). Below, we will briefly discuss the strategies used by EBV to evade the innate and adaptive immune responses.

### Innate immune response and EBV evasion

The innate immune response against EBV originates from both the EBV-infected cells themselves (B and epithelial cells) as well as from bystander cells like myeloid and NK cells. One of the major elements of innate immunity are the pattern recognition receptors (PRRs) that can recognize a diverse array of pathogen associated molecular patterns (PAMPs) and recruit downstream effector mechanisms, such as secretion of type I interferons in response. To date, 10 TLRs have been identified in humans. TLR9 is the key receptor for sensing EBV and is abundantly expressed in B cells and certain myeloid cells. TLR9 specifically senses unmethylated CpGs of EBV DNA motifs present in viral particles immediately after primary infection in B cells. Upon stimulation, TLR9 can activate the NF-κB pathway, which in turn promotes production of pro-inflammatory cytokines and B cell proliferation.

Dendritic cells (DCs) can sense, phagocytose, process and present antigens to cells of the adaptive immune system. DCs are generally classified into two types, conventional DCs (cDCs) and plasmacytoid DCs (pDCs), which express TLR3 and TLR9, respectively. Unlike TLR9, TLR3 is endosomally located and recognizes dsRNAs, including EBV-RNAs (EBERs). Nevertheless, both TLR3 and TLR9 stimulate type I IFN production when triggered. Even monocytes and macrophages sense EBV *via* TLR3 and TLR9 leading to cytokine and chemokine production. Another interesting cellular player are the NK cells. NK cells are critical cytotoxic innate lymphocytes that target infected cells. Like DCs, NK cells have two broad subsets, CD56^bright^ and CD56^dim^, the latter being more relevant in B cells restricted EBV infections (248). This is supported by studies which suggest that deficiencies in NK cells can increase the occurrence of EBV-driven pathologies. Consistent with this notion, NK cells recognize and preferentially target infected cells with lytic EBV infection (249, 250). Despite the intricate network of innate immune players, EBV has developed strategies to counteract innate immunity. For example, EBV can reduce expression of several TLRs. For example, EBV lytic protein BGLF5 reduces TLR9 expression and LMP1 suppresses TLR9 function in EBV^+^ PTLDs and cHLs. A detailed review of interplay between EBV and host innate immune responses can be found elsewhere (251).

### Adaptive immune response and EBV evasion

Adaptive immunity can be broadly classified into humoral and cell-mediated processes, which are mediated primarily by B and T cells, respectively. Adaptive humoral responses are the direct product of interaction between antigens and immunoglobulin (Ig) on the surface of naïve B cells, which leads to secretion of antigen-specific antibodies and antigen presentation to T cells. T cells in turn help B cells with Ig-class switching and affinity maturation either specifically *via* CD40L/CD40 binding or non-specifically *via* interleukin/cytokine release (252). Primary EBV infection triggers an immediate IgM response to viral capsid antigen (VCA) and *BMRF1* encoded early antigen diffuse (EaD) complexes. The importance of humoral immune responses and molecular details of antibody response against EBV has been previously reviewed (252). EBV-specific T cells are key players in determining the fate of EBV infected cells. Both types of T cells, namely, CD8^+^ cytotoxic and CD4^+^ helper T cells can recognize EBV antigens presented on the surface of infected cells by HLA molecules (253, 254). While HLA class I antigens are expressed on almost all nucleated cells, HLA class II antigens are expressed on the surface of antigen presenting cells (APCs) (253). Despite increased infiltration of CD8^+^ T cells in EBV^+^ tumors compared to EBV^–^ tumors (255), EBV has developed several strategies to evade T cell responses, for example by downregulating HLA expression, blocking antigen presentation pathways or creating an immunosuppressive TME. The latter is mediated by increased production of immunosuppressive cytokines and/or increased expression of immune checkpoint molecules that are known to induce T cell exhaustion (229).

Glycoprotein programmed death ligand 1 (PD-L1) represents one of the several immune checkpoint molecules that is modulated by EBV and is used as a mechanism of immune evasion by many tumors. This occurs as a function of PD-L1 engagement with cell surface receptor programmed death 1 (PD-1 or CD279) expressed on T cells (256). PD-1 interacts typically with its ligand PD-L1 (CD274 or B7-H1) and less frequently with PD-L2 (CD273 or B7-DC) (257). While PD-L1 is expressed by a wide-range of cell types, the latter is expressed only by specific cell types, including DCs, mast cells and macrophages (258). PD-1 is an inhibitory receptor that is rapidly upregulated upon antigen-mediated T cell receptor (TCR) stimulation (259). Recent studies have identified PD-1 expression in a plethora of other immune cell subsets such as B cells, DCs, NK cells, and monocytes (260, 261). The PD-1/PD-L1 pathway plays a crucial role in immune tolerance by fine-tuning the quality and duration of T cell response thereby serving as a ‘rheostat’ of immune response (262, 263). This is partly achieved by counterbalancing the T cell activation signal triggered by binding of CD28 on T cells with CD80/CD86 on APCs. The binding of PD-1 receptor with its cognate ligands attenuates TCR signaling and leads to T cell exhaustion (264). This inhibitory interaction serves to protect target tissues from hyper-activated immune mediated damage. Tumor cells take advantage of this mechanism by frequently upregulating PD-L1 expression to escape the host anti-tumor immune response (265). Consequently, the PD-1/PD-L1 axis serves as one of the promising targets for immunotherapy in such malignancies. Infectious viruses such as Epstein-Barr virus (EBV), hepatitis C virus (HCV) and hepatitis B virus (HBV) leverage the PD1/PD-L1 pathway to facilitate escape of infected cells from the antiviral immune response (266). Consistently, PD-L1 expression is higher in EBV^+^ relative to EBV^–^ tumors in NPC, GC and DLBCL (180).

Several mechanisms have been reported for increased PD-L1 expression. These include alterations at the genetic level, specifically the amplification of the chromosomal region 9p24.1, which includes the genes PD-L1, PD-L2, and JAK2 (217, 267). Such genetic alterations have been associated with certain B-cell lymphomas and gastric cancer (268–271). Interestingly, in ~40% of cHLs, increased PD-L1 expression is not due to this amplification but attributed to upregulation by certain EBV-encoded gene products (272). The dysregulated expression of PD-L1 in cancer has been attributed to the oncogenic activation of multiple signaling pathways, including JAK/STAT, PI3K/Akt/mTOR, MEK/ERK, and Jun/AP-1 which can either act independently or synergistically to regulate PD-L1 expression (273–275). In addition, another important mechanism of PD-L1 upregulation is 3’-UTR disruption of PD-L1 by EBV insertion at this locus (276). EBV encoded genes are also known to modulate host immune responses. BZLF1 (Zta) can induce expression of host immunosuppressive genes, such as *TGFB1*, which further downregulate expression of immune responsive genes, such as *TLR9*, *IFI6*, and *IL23A* (277, 278). LMP1 promotes AP-1, JAK-STAT and NF-κB signaling mediated activation of PD-L1 (279–281), suggesting that LMP1-mediated signaling might also be a key player in the immune escape strategy in cancers that express LMP1 (e.g. NPC, cHLs and DLBCLs). LMP1 also promotes proliferation and survival and LMP1-driven PD-L1 upregulation correlates with poor prognosis in certain lymphomas (282). Lack of LMP1 expression in EBV^+^ eBLs, consequently, is associated with absence of PD-L1 expression observed in these tumors (283). Likewise, EBNA1 also modestly promotes IFN-γ-induced PD-L1 overexpression in GC cell lines (284). The role of other EBV genes in inducing PD-L1 expression in EBV-associated cancers is less clear and further investigation is needed to determine how different viral gene products affect immune responses and PD-L1 expression in different EBV-associated cancers.

## Therapeutic strategies for targeting EBV-associated malignancies

**Figure 3:**
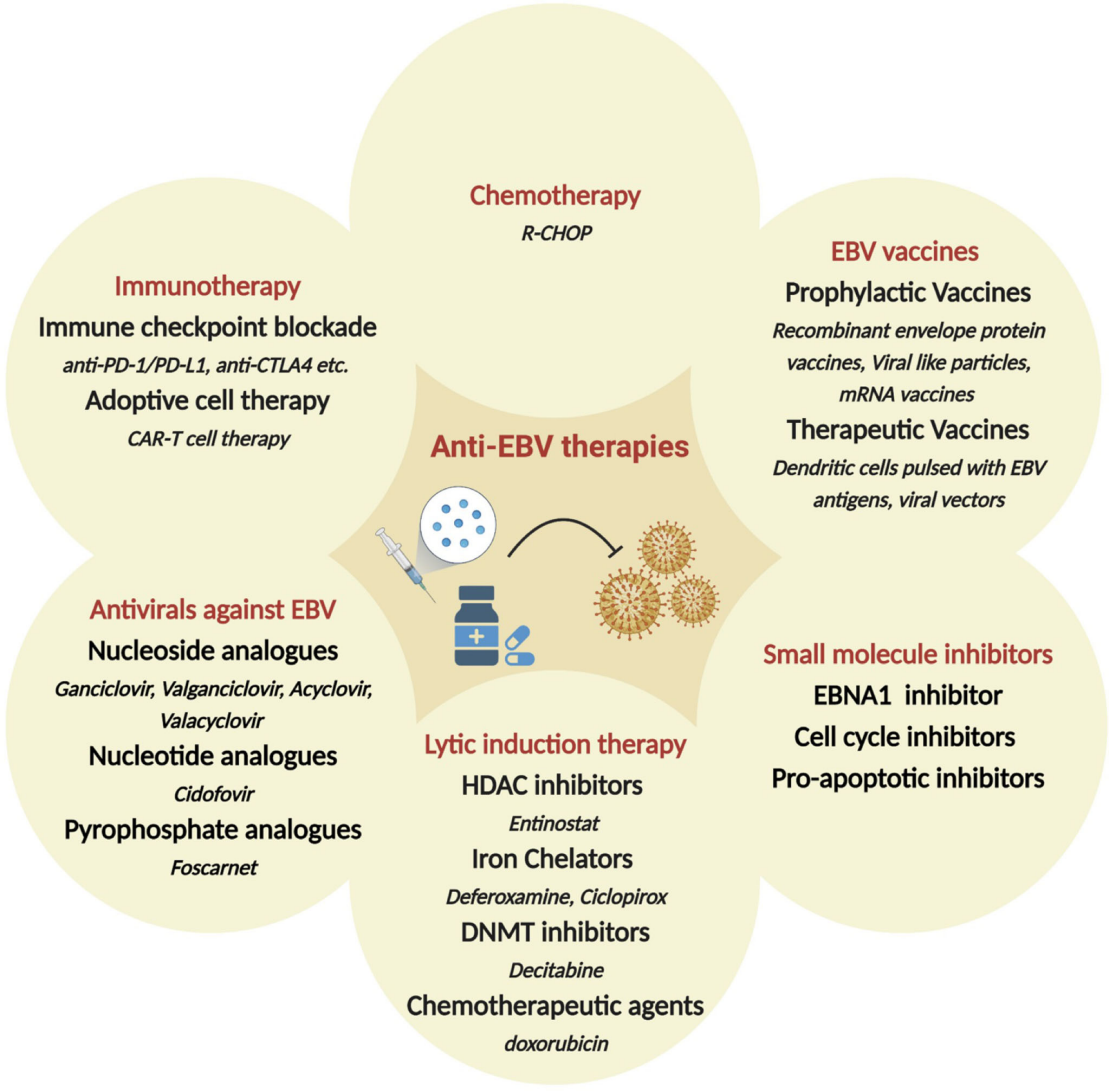
Therapeutic approaches hypothesized or in clinical use, for the treatment of EBV-associated malignancies. Shown are broad categories of treatments (red), subcategories (bold) and specific examples (italicized). HDAC: Histone deacetylase; DNMT: DNA methyltransferase; R-CHOP: Rituximab plus cyclophosphamide, doxorubicin, vincristine and prednisone.

Since EBV contributes to malignant cell transformation and is found in almost every cell of EBV^+^ tumors, it has been considered a potential target for precision medicine and individualized cancer treatment. While non-specific chemotherapy is typically the first line of therapy, several additional strategies have been proposed specifically targeted towards EBV^+^ malignancies (**Figure 3**). These include i) antivirals against EBV; ii) small molecule inhibitors for EBV-encoded gene products, such as LMP1 and EBNAs; iii) induction of the lytic form of EBV replication in tumor cells in combination with prodrugs that are cytotoxic in lytically infected cancer cells; iv) enhancing the host immune response to viral antigens expressed by EBV-infected tumor cells; v) use of EBV vaccines. Additional strategies that are under consideration include induction of EBV episome loss by treating tumors cells with low-dose hydroxyurea and expressing toxic genes using EBV-dependent approaches (285, 286). There is also considerable enthusiasm for immune checkpoint therapies for the management of EBV associated cancers. Some are approved or under investigation in clinical trials for the treatment of NPC, GC, and HL. Based on initial trial reports, PD-1 targeting treatments, such as pembrolizumab and nivolumab, seem to improve longevity and/or partial response, especially in patients with PD-L1^+^ tumors (287–293). Here, we will discuss some of the major therapeutic strategies for EBV^+^ cancers, focusing on recent developments and highlighting current gaps and/or challenges.

### Chemotherapy

For lymphomas, such as BLs, the conventional chemotherapeutic regimen include R-CHOP (Rituximab plus cyclophosphamide, doxorubicin, vincristine and prednisone), CALGB (cancer and leukemia group B), Hyper-CVAD +/- R (cyclophosphamide, vincristine, doxorubicin, and dexamethasone, with or without Rituximab), CODOX-M/IVAC +/- R (cyclophosphamide, vincristine, doxorubicin, methotrexate, ifosfamide, etoposide, and cytarabine, with or without Rituximab), and dose-adjusted (DA) R-EPOCH (rituximab, etoposide, prednisone, vincristine, cyclophosphamide, and doxorubicin) (294). Although BLs and HLs are generally curable, these regimens for relapsed aggressive B cell lymphomas are typically ineffective. Currently a novel chemotherapeutic approach is under investigation in a phase II clinical trial (NCT01964755) for relapsed EBV-associated lymphomas that utilizes a combination of drugs to potentiate the function of zidovudine (ZDV) to suppress NF-κB and viral latency.

Unlike in lymphomas, there is limited evidence on the clinical gains of chemotherapy alone in EBV^+^ epithelial cancers. Surgery (typically gastrectomy) and chemotherapy remain the first line of treatment for patients with EBV^+^ gastric cancer. Nevertheless, the efficacy of chemotherapy remains speculative. Corallo et al. reported that 6 EBV^+^ GC patients who received fluorouracil and platinum as first-line of chemotherapy had a 3-year survival rate of 80% compared to 26.5% in EBV^–^ GC patients (295). However, another observational cohort study with 31 patients reported an overall response rate of only 29% in metastatic EBV-GC patients who received taxane/trastuzumab, fluoropyrimidine and platinum as the first-line therapy (296). Owing to the single center nature of these studies and small sample sizes, it is necessary to confirm these observations in larger cohorts and clinical trials (297). From the limited evidence it seems that patients with EBV^+^ GC have few metastases, longer survival, and high disease control rates. Although, chemotherapy helps some patients by increasing the frequency of event free and overall survival, it is still insufficient to treat EBV^+^ cancers completely and eradicate infected cells. Combining chemotherapy with immunotherapy has provided encouraging preliminary results but further exploration and development of more effective combinatorial strategies are required.

### Adoptive cell therapy

Adoptive cell therapy (ACT) is a form of immunotherapy where specialized *in vitro* expanded or modified immune (usually T) cells are transferred to patients to enhance or repress immunity. These T cells are either specific for an antigen (e.g. viral protein or tumor-associated antigen) or are genetically engineered to express chimeric antigen receptor (CAR) or modified T cell receptor (TCR). The first ACT was performed in 1994, where donor leukocytes which included EBV-specific cytotoxic T cells (CTLs) were infused into 5 patients who had developed EBV-associated PTLD. Complete remission was observed in 5/5 patients, however all of them developed graft-versus-host disease (GVHD) due to alloreactive T cells (298). Since then, significant progress has been made in the field of ACT for patients with EBV-associated PTLDs and the concept is expanding to include patients with NPC and HL, as summarized elsewhere (299). One of the strategies to minimize alloreactivity (i.e. to reduce the risk of GVHD) includes infusion of *in vitro* stimulated and expanded EBV-specific CTLs that are donor-derived (300, 301). Interestingly, adoptively transferred CTLs not only restore the anti-EBV immune response but can also establish long-term persistence (302, 303). Similar strategies for ACT have been developed for patients with NPC and HL, where CTLs specific to EBV latent antigens (e.g. EBNA1, LMP1, LMP2) are expanded *ex vivo* and infused into patients (304). Phase I/II clinical trials with such immunotherapy approaches have increased the overall survival of patient with recurrent or refractory NPC (305–307) and HL (308).

A modified version of ACT for the treatment of EBV^+^ cancers is to engineer T cell receptors before transferring T cells to the patients. Such EBV-specific TCR-engineered T cell therapy is based on the rationale that TCRs on CD8^+^ T cells can be re-engineered to specifically recognize EBV latent and lytic proteins. The stability and anti-tumor effect of these chimeric TCRs have been evaluated in murine models and provide encouraging results (309). For instance, T cells expressing LMP1-specific TCR inhibited tumor growth and prolonged survival in xenograft mice (310). Another type of ACT being widely investigated is EBV-specific CAR T cell therapy. CAR-T cell therapies targeting specific antigens, for instance CD19, CD20, CD22 and CD30, have provided encouraging results for treatment of lymphoma in clinical trials (311–313). One drawback of CAR T cell therapy is that CD8^+^ T cells engineered with a CAR will also express their own native TCR, so the potential for auto-reactivity remains. Another the major limitation of CAR-T cell therapy is specificity for tumor-associated antigens, as some of these might be expressed by normal cells. This leads to adverse off-tumor toxicity, cytokine release syndrome and deficiencies in B-cell mediated humoral responses. Some of these limitations have been addressed by developing CAR-T cells that are specific to EBV antigens such as LMP1, since they would be specifically expressed in EBV infected malignant cells (314). In fact, LMP1-specific CAR-T cells exhibit enhanced tumor inhibition in LMP1-positive NPC xenograft mouse models (315). The translation of these therapies for the treatment of EBV-associated cancers warrants further evaluations before it can be prescribed as personalized immunotherapy for EBV^+^ cancers in humans.

### Antiviral therapy

Several antiviral drugs have been identified and are being currently evaluated for clinical use. These can be broadly divided into three classes: 1) nucleoside analogs such as acyclovir (ACV), ganciclovir (GCV), penciclovir (PCV), and their oral prodrugs valacyclovir (VACV), valganciclovir (VGCV) and famciclovir (FAM), respectively; 2) nucleotide analogs such as cidofovir (CDV); and 3) pyrophosphate analogs, including foscarnet. Although these antiviral agents have been clinically evaluated for different viruses, their clinical utility in the context of EBV-associated malignancies is lacking. To date, there is no effective Food and Drug Administration (FDA) or European Medicines Agency (EMA) approved antiviral therapy available for EBV infections. Nevertheless, we will briefly discuss some of these antiviral agents in the context of EBV-associated diseases. A more in-depth review of these drugs can be found published elsewhere (316).

Nucleoside analogs such as ACV and GCV inhibit EBV *in vitro*. The antiviral effect of ACV is attributed to the preferential incorporation of its triphosphate into the viral DNA due to high-affinity interaction with EBV polymerase compared to the cellular polymerase. This process irreversibly and specifically terminates viral DNA elongation and replication (317). The effective dose of ACV against EBV is orders of magnitude lower (0.3μM) than host cells (250 μM) (318), resulting in a highly favorable therapeutic index and toxicity profile. The antiviral effect of GCV is greater than ACV but it is more toxic. Antiviral (e.g. ACV, GCV or VACV) prophylaxis has significantly reduced development of PTLD in high-risk EBV-seronegative lung transplant patients (319) and reduces EBV viremia in pediatric renal transplant patients (320). Clinical trials administering ACV along with prednisolone have shown the inhibition of EBV replication in the oral cavity, however they do not alleviate the duration or intensity of clinical symptoms (321). Importantly, none of the nucleoside analogs have any effect on latent infections. This is because the viral enzymes that are needed for the prodrug activity are not expressed during latent phase (322). Other nucleoside analogs with efficacy against varicella zoster virus, such as omaciclovir, have not yet been evaluated against EBV (323).

Cidofovir is a nucleotide analog that possesses both antiviral and antiproliferative properties and is metabolized into its active form by cellular kinases (324). The antiproliferative effect of Cidofovir on EBV-infected NPCs has been previously reported (325). Consistently, intra-tumoral injection of cidofovir suppresses tumor growth in EBV^+^ NPC xenografts in nude mice (326). Another study showed that treatment of NPC (C15) and BL (Raji) cell lines with cidofovir decreases expression of LMP1 and EBNA2 oncoproteins and increases apoptosis and enhances ionizing radiation (IR)-induced regression of EBV^+^ NPC and BL tumor bearing nude mice (327). Tenofovir (TFV) is an acyclic nucleoside/nucleotide analog that has already been approved for the treatment of HIV and HBV infection, where it acts as an inhibitor of the viral reverse transcriptase (328). The prodrugs of tenofovir, disoproxil fumarate (TDF) and tenofovir alafenamide (TAF), are both orally bioavailable and are more potent than ACV, PCV and GCV (329). All of these antiviral agents target EBV DNA polymerase, however, unlike others, the tenofovir prodrugs are metabolized independently of viral enzymes to their active forms and depend on host enzymes, thus, permitting their usage for latently infected cells (330). Despite the availability of a plethora of antiviral agents, there aren’t any effective antivirals for EBV-associated cancers. However, studies on compounds like tenofovir holds some promise and warrants further study (331).

Foscarnet is a pyrophosphate analogue with a broad antiviral activity against the *Herpesviridae* family (332). As a pyrophosphate analogue, it disrupts viral DNA polymerase activity by inhibiting cleavage of pyrophosphate from the nucleoside triphosphate. Unlike ACV and GVC, foscarnet does not depend on viral protein kinases for its activity, making it useful in cases of ACV/GCV acquired resistance. However, it might be less tolerated in patients due to increased toxicity. Several case reports have shown benefits of foscarnet in treatment of EBV^+^ PTLDs (333, 334). However, the systematic efficacy of foscarnet in treatments of EBV-associated needs to be further evaluated.

### Lytic induction therapy

**Figure 4:**
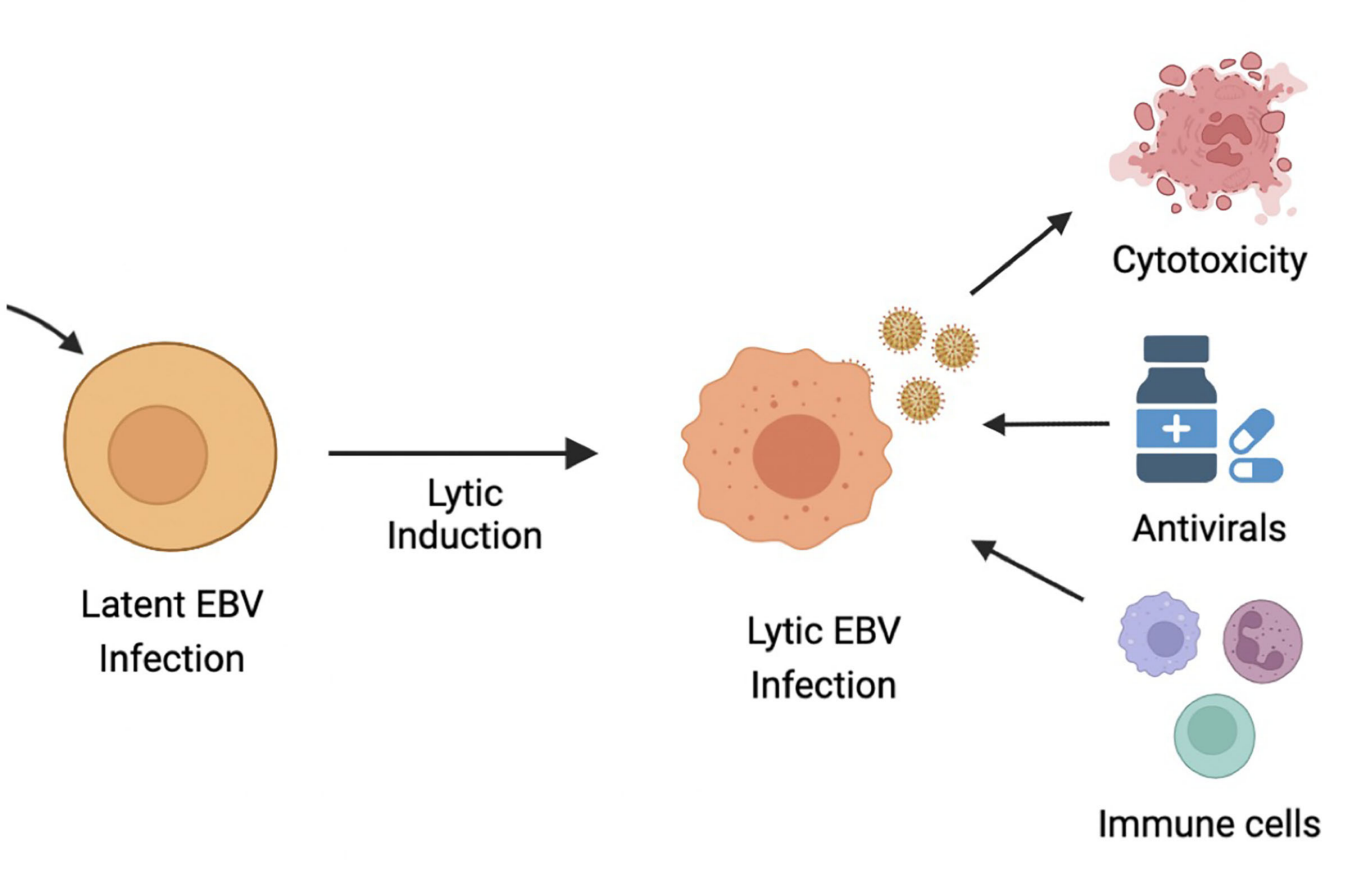
Ltic induction or cytolytic virus activation therapy (CLVA). CLVA can make latently infected cells susceptible to antivirals ang immune recognition.

As discussed, latent EBV infection is associated with human malignancies, such as BL, PTLD, NPC, GC, HL and non-HLs. GCV and ACV are commonly used antiviral drugs that require the EBV lytic encoded protein kinase (EBV-PK) and thymidine kinase (EBV-TK) for the conversion of pro-drugs into active viral drugs. As a result, these drugs are inefficient in eliminating EBV-infected cells that are in the latent state. One therapeutic approach is therefore the induction of EBV lytic replication, also known as cytolytic virus activation (CLVA), in combination with antiviral drugs to enable specific targeting of tumor cells that harbor EBV in a lytic state (18, 335). CLVA within infected tumor cells can induce i) a cytotoxic or cytostatic effect from the lytic viral proteins; ii) expression of viral enzymes that metabolize and activate antiviral pro-drugs, such as ACV and GCV; and iii) a range of antigenic viral proteins that can now be recognized by host-immune cells (336) (**Figure 4**).

Although, several classes of lytic inducers have been identified and their mechanism of action has been elucidated in different cell types, only one clinical study has reported a promising outcome in a small fraction of patients with EBV^+^ tumors (337, 338). This is primarily because these compounds have three major drawbacks limiting their use in clinical settings. First, most of these compounds have low efficiency and can induce the lytic cycle in only a small percentage of cells, therefore a considerable proportion of cells are refractory (339). Second, the efficiency of these lytic inducers is heavily dependent on the cell type, thus cannot be broadly utilized for all EBV-associated malignancies. Many lytic inducers also have serious side effects, therefore their translation into clinical use is challenging. Lastly, there is a concern that chemical induction of EBV could promote viral dissemination (340, 341).

Over the years, scientists have identified several classes of organic and chemical compounds that are able to induce the EBV lytic cycle in latently infected cells. Protein kinase C (PKC) activators (PMA), HDAC and DNA methyl transferase (DNMT) inhibitors, chemotherapeutic agents and anti-IgG are among the known classes of lytic inducers. Interestingly, evidence suggests that several distinct mechanisms of lytic induction may exist because synergistic effects have been observed when different lytic inducers are combined. For instance, treatment of BL and GC cell lines with combinations of PMA and sodium butyrate or valproic acid and cisplatin leads to significantly higher EBV lytic reactivation compared to individual treatments. Large-scale chemical library screens in GC and NPC cells have identified two additional distinct compounds that could induce EBV reactivation, one that resembles iron chelators and one that activates the MAPK pathway (342). Different classes of lytic inducers has been previously reviewed here (339). In this section, we will briefly discuss HDAC inhibitors and iron chelators that have been reported to induce the EBV lytic cycle *via* PKC-δ and HIF-1α pathways, respectively (343).

### Histone deacetylase inhibitors

Of all the different inducers of the EBV lytic cycle, inhibitors of histone deacetylase (HDACi) have been well studied. These include sodium butyrate (NaB), valproic acid (VPA), suberoyl anilide hydroxamic acid (SAHA or Vorinostat), and romidepsin. Use of HDACi alone or in combination with GCV are currently being tested for use in patients. For example, butyrate in combination with GCV has shown promising results in patients with refractory EBV^+^ lymphoid malignancies (344). However, the efficacy of this combination therapy has received limited success *in vivo* due to the poor oral bioavailability and short half-life of butyrate (345). A systematic study of HDACi have ranked panobinostat, belinostat, butyrate, entinostat, oxamflatin, apicidin, and largazole from highest to lowest in their ability to induce EBV-TK and EBV-PK kinases, suggesting that despite the structural diversity, most HDACi can function as inducers of EBV lytic replication (346). Interestingly, despite belonging to the same class, these HDACi might invoke different mechanisms of action to induce EBV reactivation. For instance, VPA antagonizes the ability of other HDACi to induce EBV lytic reactivation (347).

### Iron chelators

Iron is a nutrient that plays an important role is various aspects of cell biology including growth and differentiation. It is used by heme-containing proteins and serves as a cofactor for many enzymatic activities. Desferioxamine is a widely used drug to treat iron-overload. Desferrithiocin, an orally available iron chelator, is a more potent substitute of Desferioxamine due to higher bioavailability. Both these compounds adversely inhibit proliferation of T cells, which can be rescued by the addition of iron (in the form of ferrous chloride, FeCl_2_) (348). Iron chelators induce the EBV lytic cycle in certain cancer cells by inhibiting enzymatic activity of protein hydroxylases (349). A novel compound, named C7, has been recently described and reported to induce early, but not late, lytic proteins *via* intracellular iron chelation, alleviating the concern about viral dissemination (342, 350). However, C7 seems to direct only a small proportion of cells into the lytic cycle. Moreover, due to the abundance of iron *in vivo* and within the TME, delivery of iron chelators into tumors remains challenging, suggesting that further studies are needed to identify novel compounds and/or overcome these limitations.

### Small molecule inhibitors

Due to highly penetrant effects, small molecule inhibitors have been of great interest in treating various cancers. In viral-associated cancers, essential viral proteins are great targets because inhibitors against them might have lower toxicity against host cells. In EBV-associated cancers, since EBNA1 has served as a promising target for virus-targeted therapies as it has no cellular homologue and is constitutively expressed in all EBV^+^ cancer cells. There are several ways by which EBNA1 can be functionally perturbed, and significant advances are currently being made in the field of EBNA1 targeting therapies. These include inhibiting DNA binding activity of EBNA1 (351, 352), disrupting homodimerization (353), blocking its interaction with key host cellular proteins, such as USP7, CK2 or targeting its oncogenic partners, such as MDM2 (354). VK-2019 is a small molecule inhibitor that binds to EBNA1 and disrupts its DNA binding activity. It is administered orally and is currently undergoing phase 2 clinical trial (NCT03682055) for patients with advanced NPC. Further studies are needed to identify specific inhibitors of other latent EBV proteins, including LMP1, or viral genes that are essential for EBV transformation.

### Prophylactic EBV vaccines

To our knowledge, there are currently no FDA-approved vaccines for EBV. Since EBV is a causative agent for a range of diseases a prophylactic or preventative vaccine would be the most beneficial and cost-effective therapeutic approach to manage EBV-associated IM, malignancies and autoimmune diseases. The rationale for prophylactic vaccination is to prevent EBV infecting its target cells by inducing an antibody response. Below we will briefly discuss strategies for developing prophylactic vaccines against EBV.

Considering that EBV requires multiple envelope proteins to enter target cells, these serve as excellent candidates for developing recombinant envelope protein vaccines. EBV glycoprotein gp350 (BLLF1), is the most abundant envelope protein, and initial studies on EBV vaccine development primarily focused on gp350 (355, 356). However, a large-scale clinical trial using soluble gp350 failed in preventing infection, albeit reducing the development of IM after EBV infection (357). In addition, gp350 vaccine candidates only protect B cells but no other EBV target cells (e.g. epithelial cells) from infection. EBV glycoproteins gH/gL and gp42 bind to HLA-DR on B cells and integrins and ephrin receptor A2 on epithelial cells, respectively, and facilitate EBV fusion to the cell membrane (70, 358–360). Since, these viral glycoproteins are integral components of the core viral fusion machinery, they also serve as excellent candidates for prophylactic vaccine development (299). To this end, in 2019, Bu et al. developed an EBV gH/gL/gp42 based nanoparticle vaccine. This vaccine inhibits EBV infection of both epithelial and B cells by eliciting an antibody response that targets the virus membrane-fusion proteins in mice and non-human primates-macaques (356). This year, a novel EBV gp350-ferritin nanoparticle vaccine was developed by researchers in NIAID/NIH that has entered the Phase I clinical trial to determine its safety and immunogenicity in humans (NCT04645147). Taken together, these studies suggest that targeting multiple EBV glycoproteins - gH/gL, gB and gp350 - together could synergistically induce highly effective EBV neutralizing activity. Additionally, evidence suggests that these viral glycoproteins can also induce T cell immune responses to further enhance vaccine efficacy by recruiting T cells to either kill or inhibit transformation of recently infected cells if neutralizing antibodies are ineffective, for example due to variations in EBV protein sequences (361, 362).

Recombinant viral vectors are also commonly used to develop therapeutic vaccines. Essentially, these are live viruses that are engineered to express specific proteins that help elicit an immune response. Such vaccines can infect target cells and induce a CD8^+^ T cell response, enhance the anti-inflammatory response by serving as adjuvants themselves and have high gene transduction efficiency (363). The first EBV vaccine was developed in 1995 and tested in humans. This was a live recombinant vaccinia-based virus, expressing EBV envelope protein BLLF1/gp350 (364). However, this vaccine was discontinued due to adverse effects. In 2004, Taylor et al. developed a chimeric antigen construct using a modified vaccinia virus “Ankara” (MVA) vector that encoded the C-terminal portion of EBNA1 and entire LMP2 (MVA-EL). Upon transduction, the EL protein can be processed by HLA I and II, resulting in CD8^+^ and CD4^+^ T cell responses (365). Since these two EBV latent proteins are expressed in NPC, MVA-EL was tested for safety and immunogenicity as a therapeutic vaccine for patients with EBV^+^ NPCs in phase I clinical trials (NCT01147991). Indeed, this vaccine was well tolerated and induced EBV-antigen specific T cell responses in 8/14 patients in UK and 15/18 patients in Hong Kong (366). Further studies are needed to determine its translation to the clinical setting for treatment, either alone or in combination with other modalities including T cell therapies. In 2012, another group developed and evaluated the ability of a recombinant adenoviral vector-based vaccine (AdE1-LMPpoly) to induce EBV-specific T cell responses in recurrent or metastatic NPC in a phase I clinical trial (ACTRN12609000675224). Encouragingly, EBV-specific T cells were expanded in 16/24 NPC patients and infusion of AdE1-LMPpoly–generated T cells was tolerated and prolonged survival by 2.3-fold. A phase II randomized clinical trial is necessary to confirm these observations (367). A comprehensive list of current vaccines is available here (368, 369).

In addition to the EBV envelope protein-based vaccine and recombinant viral vectors, development of viral like particle (VLP) vaccines is another area of active research. The design of such viral particles is based on the rationale that a non-infectious version of EBV will elicit an EBV-specific innate and adaptive immune response in a safe and effective manner (370). Mechanistically, VLPs are phagocytosed and processed by DCs. DCs then activate CD8^+^ and CD4^+^ T cells by presenting viral antigens on HLA class I and II, respectively (371). While CD8^+^ T cells mount a cytotoxic response, CD4^+^ T cells elicit an anti-tumor response *via* Th1 and Th2 type responses that produce pro-inflammatory cytokines (e.g. IFN-γ, TNF-α, and IL-4, IL-10, respectively) (371). The status of VLP developments against oncoviruses and their biological and chemical characterization has been recently reviewed (372). In 2015, a novel EBV vaccine based on the Newcastle disease virus (NDV) VLP platform was developed, consisting of EBVgp350/220 ectodomain fusion protein that structurally mimicked EBV. This VLP elicited a long-lasting neutralizing antibody response in mice, but the responses were comparable to soluble gp350/220 (373). Thus, a more immunogenic VLP was developed that incorporated additional EBV glycoproteins and latent antigens – EBNA1 and LMP2. Immunization with gH/gL-EBNA1 and gB/LMP2 VLPs produced high neutralizing antibody titers *in vitro* and EBV-specific T cell responses in vaccinated BALB/c mice (374). DNA-free VLPs/LPs typically consists of EBV structural proteins that are weakly immunogenic towards CD8^+^ T cells. As a result, humoral and cell-mediated immune responses that recognize these structural proteins offer limited to no protection against latently infected cells. Another strategy is to use EBV particles themselves for VLP vaccines. The first EBV VLP was created by removing the terminal repeats that result in production of large amounts of defective viral particles without the viral DNA that could bind to both B and epithelial cells (375, 376). In subsequent studies, more viral packaging proteins (BFLF1, BFRF1, BBRF1) and viral oncogenes (EBNA2, 3A, 3B and 3C, LMP1 and BZLF1) were deleted to improve the safety profile of these VLPs, while maintaining immunogenic potential (377, 378). In 2018, a more immunogenic EBV VLP was created by fusing EBNA1 and EBNA3C to the EBV tegument protein BNRF1. As a result, only 14% of mice vaccinated with modified VLPs had detectable viral load in the peripheral blood compared to 100% of the control PBS-vaccinated mice (379). While, VLP-based therapeutics are being developed and evaluated in early clinical trials, none have yet reached the phase III efficacy clinical trial stage. This is attributed to two major limitations. First, they suffer from production efficiency and scalability, which is partly because of the use of mammalian cells that lead to low viral titers and the presence of contaminants from human producer cell lines. Second, they offer low immunogenicity as epitope-based vaccines. This necessitates the need to co-administer an adjuvant or design better antigen delivery systems. Nonetheless, there are some promising ongoing studies on EBV-derived VLP vaccines to prevent EBV^+^ cancers.

With the success of mRNA vaccines against SARS-CoV2, researchers have developed an EBV mRNA vaccine based on the same platform. Moderna has recently launched phase I clinical trial of its EBV mRNA vaccine (mRNA-1189) that have shown high EBV neutralizing antibody titers in mice (NCT05164094). This investigational vaccine targets EBV glycoproteins - gp350, gB, gH/gL and gp42 - and is hypothesized to prevent IM and EBV infection. Despite the potential challenges with mRNA-based approach for an asymptomatic virus like EBV (299), results from these trials will help in developing effective preventative treatment approaches for EBV infections and related disorders.

## Preclinical models to study EBV biology

There are several malignancies that associated with EBV infection. Despite decades of research in this field, the precise role of this virus in the tumorigenic process and immunoevasion is not fully understood. While EBV^+^ cell lines, including LCLs, serve as a good model system to study EBV-host biology *in vitro*, mouse models help investigate and understand EBV-specific biology *in vivo*. Since the γ-herpesvirus have co-evolved along with their hosts, i.e. humans and monkeys, there is a lack of suitable counterparts in rodents (380). Unfortunately, the murine γ-herpesvirus 68 (MHV-68) lacks EBV’s transforming ability and thus fails to recapitulate EBV-induced tumorigenesis (380). Consistently, major differences in molecular mechanisms, tumorigenesis, cellular tropism, and immune responses between MHV-68 and EBV infections have been observed (381). As such, murine models used to study EBV-associated malignancies are typically immunodeficient. Transferring human peripheral blood mononuclear cells (PBMCs) from EBV-seropositive human donors into immunodeficient mice can generate PBMC-derived EBV^+^ B cell tumors (382). Zhang et al. further developed a genetically engineered mouse model to study EBV-driven lymphomas found in immunosuppressed patients (383), underscoring the relevance of immunodeficient murine models for the study of EBV disease (382). However, since these mice are immunocompromised, they are unable to induce a host immune response. Transferring human PBMCs to overcome this barrier often leads to severe xeno-graft versus host disease (GVHD) in these mice, limiting the duration of the study (384). Recognizing these limitations, the field has shifted to developing and using lymphocyte-deficient mice which have the potential to be reconstituted with human immune components (385). These mice are valuable resources to investigate human specific viral infections, as well as develop and test therapeutic vaccines (386). A more comprehensive review of murine models for EBV-associated tumorigenesis can be found elsewhere (386, 387). Despite being an excellent model system for EBV-associated lymphomas, these mice are not suitable to study EBV^+^ epithelial cancers since the mice lack human epithelial cells. In these cases, nonhuman primate animal models might be of justified use. These have been reviewed by others (388).

## High-throughput approaches to study EBV-associated cancers

With the advent of massively parallel sequencing technologies, researchers have been able to better characterize the complexity of interactions between host and viral genes and identify novel genomic and epigenomic alterations within EBV-infected cancer cells that are druggable. Specifically, these technologies enable the capture of all the genetic material inside host cells, which can then be used to simultaneously study the biology of both host cells and infecting viruses (180, 389–392). These studies specifically overcome a limitation of laborious traditional EBV genetic studies where only individual viral or host genes are studied in isolation outside of the tumor context. In this section, we will discuss some of the sequencing approaches that are used to explore the genetic, transcriptomic and epigenomic landscapes in the context of EBV-associated malignancies.

### Whole genome sequencing

The investigation of EBV genome sequences is important due to their association with several human malignancies (165). Prior to 2013, GenBank had whole genome sequences from less than 10 strains of EBV (393). Conventional sequencing techniques were inefficient, expensive and could help investigate only a few viral genes at a time. They typically involved digestion of genomic DNA *via* restriction enzymes, cloning and Sanger sequencing. Moreover, the large size of the EBV genome (~172kbp) added to the cost and time. Whole genome sequencing (WGS) technologies marked a new era of EBV genome sequencing and could be performed with or without EBV enrichment (180, 394). The WGS approach is quite sensitive to detecting viral derived sequences as well as integrated viral regions within the host genomes. As such, WGS has not only helped identify distinct EBV variants, mutations and oncogenes but has also allowed for a comprehensive survey of EBV integration in a wide variety of human malignancies as reviewed here (395, 396). Additionally, WGS of EBV-associated tumors have been extremely informative in identifying a range of distinct host variations that promote cancer (65, 186, 214, 217).

### RNA sequencing

The use of high-throughput sequencing technologies to study the transcriptomic landscapes of disease have become a common practice. The general workflow for such techniques begins with bulk RNA extraction from the biospecimens under investigation, followed by RNA selection (mRNA or ribosomal-free RNA), cDNA synthesis, library preparation and sequencing. In the case of viral-associated malignancies, such techniques have provided key insights into cellular genes and pathways that are affected by virus (397, 398). Additionally, since these sequencing technologies are agnostic of the origin of the RNA within the cells, they capture genetic materials of both host and infecting viruses (391, 392, 399). As such, transcriptomics studies of EBV-associated malignancies have revealed important aspects of EBV biology including expression program and its interactions with the host to promote disease (180, 390, 400, 401). For example, a large-scale transcriptomic study of EBV-associated cancers classified EBV^+^ cancer types into molecular sub-types according to activation or repression of interferon signatures which was correlated with expression of several immune checkpoint genes such as PD-L1 and IDO1 (180).

Of note, the heterogeneity of cells within the biospecimens, specifically tumors and their microenvironments, present a challenge for interpreting bulk transcriptomics data. For example, a change in expression of a gene or activation of a pathway could reflect either a change in tumor cells and/or a change in the cellularity of the TME, for example by immune cell infiltration. Recent computational tools that can deconvolute the composition of cells from bulk data have provided some remedy for this issue (402–404). Nevertheless, the accuracy of these tools remains limited, and they have not yet widely applied to study TME in EBV-associated malignancies. The advent of single-cell RNA-sequencing (scRNA-seq) has overcome this challenge and has revolutionized the field of transcriptomics and helped scientists map and generate individual cell atlases (405). Specifically, scRNA-seq has enabled the study of host-pathogen interaction in viral-associated diseases as well as cellular heterogeneity (406, 407). scRNA-seq is rapidly being adopted to study EBV-associated malignancies. For example, recent studies have revealed the landscape of both tumor and infiltrating immune cells in NPCs that are associated with prognosis (234, 235, 408). Additionally, scRNA-seq are now used to delineate rare cell subpopulations such as cancer stem cells, cell-cell interactions *via* receptor-ligand analyses, cell differentiation *via* trajectory and time-resolution analyses (409). Additional steps also allow TCR, BCR and cell-surface protein expression (CITE-seq) sequencings at the single cell level to supplement scRNA-seq for the study of the diversity of immune cells. Given these technologies, it is exciting to get detailed understanding of how EBV affects these aspects of biology across various diseases and conditions, such as response to therapeutic treatments. It is important to point out that the detection of lowly expressed genes in scRNA-seq is challenging and faces a frequent “drop-out” where it might be only sporadically detected across different cells. This could be an issue for detecting EBV genes in malignancies associated with latency where most EBV genes are expressed at low levels.

### Other sequencing technologies

Transcriptional regulation, epigenetic changes and physical interactions are paramount to EBV biology and understanding EBV-associated diseases. Appending high-throughput sequencing to traditional lab techniques such as chromatin immunoprecipitation (ChIP) has enabled genome-wide detection of transcription factor (TF) bindings and epigenetic changes, such as histone modifications and DNA methylations. For instance, ChIP-seq of EBV TFs has revealed many binding sites across the host genome, which has implications for pathogenic mechanisms (410). Conversely, many cellular TFs can also bind the EBV genome (390). ChIP-seq analysis of EBV infected GC and NPC cell lines reveal a redistribution of characteristic histone marks such as H3K4me1/3 and H3K27ac (411). Of note, one of the limitations of these technologies is their requirement for millions of cells, however, recent technologies such as CUT&RUN/Tag sequencing have resolved this issue. Additional adjustments to high-throughput sequencing technologies by ligating proximal chromatin regions (e.g., Hi-C) has enabled to study physical interactions between genomic loci. This technology has enabled the study of interactions between EBV episomes and host genomes that are consequential to host gene regulation (412, 413). There has been a large number of additional assays deployed to study specific aspects of EBV biology genome wide (414, 415). For example, assay for transposase-accessible chromatin using sequencing (ATAC-seq) has been developed to study chromatin accessibility and has been extensively used to study how EBV infection affects chromatin accessibility. It is important to note that most of these assays utilize bulk sample processing and therefore observations in heterogeneous cell populations should be carefully interpreted, as discussed above for bulk RNA-seq. Recently, the limits of some of these technologies have been pushed to the single cell level and integrated into scRNA-seq platforms to enable multiomics based investigation of EBV infection (409).

## Concluding marks

As the above studies demonstrate, EBV is a causative agent and/or associated with a plethora of diseases including cancer and autoimmunity. Despite decades of research, the underlying mechanisms governing how the interactions between EBV and host cells promote carcinogenesis are incompletely defined. As a result, effective and individualized treatments for EBV-associated diseases still remain either non-specific or lacking. Nevertheless, it is also obvious that EBV is heavily regulated by a variety of factors, and it extensively regulates cellular processes and the microenvironment. High-throughput methods are ideal for revealing complex networks of tissue and disease associations. As discussed, these technologies have their own limitations. Utilizing orthogonal and multiomics technologies can typically overcome some of these limitations. For example, one of the limitations of single-cell transcriptomics is the loss of spatial information during the tissue dissociation, which are important for understanding disease biology. Recent high resolution (down to the sub-cellular level) spatial transcriptomics methods can help overcome such issues, however, due to their recent development, they have not yet been employed to study EBV-associated diseases. Additional factors such as the microbiome might also be relevant to EBV-disease biology (416) and should be considered in designing tools and models. Rigorous computational modeling is also needed to accurately identify shared or tissue-specific signatures across EBV-associated diseases. Lastly, as discussed above, better animal models and drug delivery systems are needed in order to translate laboratory findings.

## Author contributions

All authors contributed to the article and approved the submitted version.

## Funding

This work was supported by extramural research programs of the NIH (R35GM138283) and the Showalter Trust (research award to MK). This research was supported (in part) by the Intramural Research Programs of the National Institute of Diabetes and Digestive and Kidney Diseases (project number ZIA/DK075149 to BA).

## Acknowledgments

The authors also gratefully acknowledge the SIRG Graduate Research Assistantships Award to SC and support from the Purdue University Center for Cancer Research, P30CA023168.

## Conflict of interest

The authors declare that the research was conducted in the absence of any commercial or financial relationships that could be construed as a potential conflict of interest.

## Publisher’s note

All claims expressed in this article are solely those of the authors and do not necessarily represent those of their affiliated organizations, or those of the publisher, the editors and the reviewers. Any product that may be evaluated in this article, or claim that may be made by its manufacturer, is not guaranteed or endorsed by the publisher.

## References

[1] zur Hausen H de Villiers EM. Cancer “causation” by infections–individual contributions and synergistic networks. Semin Oncol (2014) 41:860–75. doi: 10.1053/j.seminoncol.2014.10.003 25499643

[2] Varn FS Schaafsma E Wang Y Cheng C. Genomic characterization of six virus-associated cancers identifies changes in the tumor immune microenvironment and altered genetic programs. Cancer Res (2018) 78:6413–23. doi: 10.1158/0008-5472.CAN-18-1342 PMC6239894 30254145

[3] Kitsou K Iliopoulou M Spoulou V Lagiou P Magiorkinis G. Viral causality of human cancer and potential roles of human endogenous retroviruses in the multi-omics era: An evolutionary epidemiology review. Front Oncol (2021) 11:687631. doi: 10.3389/fonc.2021.687631 34778024 PMC8586426

[4] Longnecker R Neipel F. Introduction to the human gamma-herpesviruses. In: Arvin A Campadelli-Fiume G Mocarski E Moore PS Roizman B Whitley R Yamanishi K, editors. Human herpesviruses: Biology, therapy, and immunoprophylaxis. Cambridge: Cambridge University Press (2007). 21348071

[5] Epstein MA Achong BG Barr YM. Virus particles in cultured lymphoblasts from burkitt’s lymphoma. Lancet (1964) 1:702–3. doi: 10.1016/S0140-6736(64)91524-7 14107961

[6] Esau D. Viral causes of lymphoma: The history of Epstein-Barr virus and human T-lymphotropic virus 1. Virol (Auckl) (2017) 8:1178122x17731772. doi: 10.1177/1178122X17731772 PMC5621661 28983187

[7] Cohen JI. Epstein-Barr Virus infection. N Engl J Med (2000) 343:481–92. doi: 10.1056/NEJM200008173430707 10944566

[8] Balfour HH Jr. Dunmire SK Hogquist KA. Infectious mononucleosis. Clin Transl Immunol (2015) 4:e33. doi: 10.1038/cti.2015.1 PMC4346501 25774295

[9] Katz BZ Shiraishi Y Mears CJ Binns HJ Taylor R. Chronic fatigue syndrome after infectious mononucleosis in adolescents. Pediatrics (2009) 124:189–93. doi: 10.1542/peds.2008-1879 PMC2756827 19564299

[10] Luzuriaga K Sullivan JL. Infectious mononucleosis. N Engl J Med (2010) 362:1993–2000. doi: 10.1056/NEJMcp1001116 20505178

[11] Tillman H Vogel P Rogers T Akers W Rehg JE. Spectrum of posttransplant lymphoproliferations in NSG mice and their association with EBV infection after engraftment of pediatric solid tumors. Vet Pathol (2020) 57:445–56. doi: 10.1177/0300985820913265 PMC7478125 32202225

[12] Longnecker RM Kieff E Cohen JI. Epstein-Barr virus. Sixth Edition Vol. 1. Wolters Kluwer Health Adis (ESP) (2013). Fields Virology.

[13] Cohen JI Mocarski ES Raab-Traub N Corey L Nabel GJ. The need and challenges for development of an Epstein-Barr virus vaccine. Vaccine (2013) 31(Suppl 2):B194–6. doi: 10.1016/j.vaccine.2012.09.041 PMC3636506 23598481

[14] Babcock GJ Decker LL Volk M Thorley-Lawson DA. EBV persistence in memory b cells *in vivo* . Immunity (1998) 9:395–404. doi: 10.1016/S1074-7613(00)80622-6 9768759

[15] Young LS Yap LF Murray PG. Epstein-Barr Virus: more than 50 years old and still providing surprises. Nat Rev Cancer (2016) 16:789–802. doi: 10.1038/nrc.2016.92 27687982

[16] Miller G El-Guindy A Countryman J Ye J Gradoville L. Lytic cycle switches of oncogenic human gammaherpesviruses. Adv Cancer Res (2007) 97:81–109. doi: 10.1016/S0065-230X(06)97004-3 17419942

[17] Murata T Tsurumi T. Switching of EBV cycles between latent and lytic states. Rev Med Virol (2014) 24:142–53. doi: 10.1002/rmv.1780 24339346

[18] Kenney SC Mertz JE. Regulation of the latent-lytic switch in Epstein-Barr virus. Semin Cancer Biol (2014) 26:60–8. doi: 10.1016/j.semcancer.2014.01.002 PMC4048781 24457012

[19] Laichalk LL Thorley-Lawson DA. Terminal differentiation into plasma cells initiates the replicative cycle of Epstein-Barr virus *in vivo* . J Virol (2005) 79:1296–307. doi: 10.1128/JVI.79.2.1296-1307.2005 PMC538585 15613356

[20] Arvin A Campadelli-Fiume G Mocarski E Moore PS Roizman B Whitley R. Human herpesviruses: Biology, therapy, and immunoprophylaxis. In: Arvin A Campadelli-Fiume G Mocarski E Moore PS Roizman B Whitley R Yamanishi K, editors. Human herpesviruses: Biology, therapy, and immunoprophylaxis. (Cambridge: Cambridge) (2007). 21348071

[21] Alfieri C Birkenbach M Kieff E. Early events in Epstein-Barr virus infection of human b lymphocytes. Virology (1991) 181:595–608. doi: 10.1016/0042-6822(91)90893-G 1849678

[22] Rickinson A. Epstein-Barr Virus. Virus Res (2002) 82:109–13. doi: 10.1016/S0168-1702(01)00436-1 11885937

[23] Kintner C Sugden B. Conservation and progressive methylation of Epstein-Barr viral DNA sequences in transformed cells. J Virol (1981) 38:305–16. doi: 10.1128/jvi.38.1.305-316.1981 PMC171153 6264105

[24] Babcock GJ Hochberg D Thorley-Lawson AD. The expression pattern of Epstein-Barr virus latent genes *in vivo* is dependent upon the differentiation stage of the infected b cell. Immunity (2000) 13:497–506. doi: 10.1016/S1074-7613(00)00049-2 11070168

[25] David A Thorley-Lawson PD Andrew Gross MD. Persistence of the Epstein–Barr virus and the origins of associated lymphomas. N Engl J Med (2004) 350:1328–37. doi: 10.1056/NEJMra032015 15044644

[26] Tse E Kwong YL. Epstein Barr Virus-associated lymphoproliferative diseases: the virus as a therapeutic target. Exp Mol Med (2015) 47:e136. doi: 10.1038/emm.2014.102 25613733 PMC4314579

[27] Heslop HE. Sensitizing burkitt lymphoma to EBV-CTLs. Blood (2020) 135:1822–3. doi: 10.1182/blood.2020005492 PMC7243150 32437562

[28] Hislop AD Taylor GS Sauce D Rickinson AB. Cellular responses to viral infection in humans: lessons from Epstein-Barr virus. Annu Rev Immunol (2007) 25:587–617. doi: 10.1146/annurev.immunol.25.022106.141553 17378764

[29] Thorley-Lawson DA Gross A. Persistence of the Epstein-Barr virus and the origins of associated lymphomas. N Engl J Med (2004) 350:1328–37. doi: 10.1056/NEJMra032015 15044644

[30] Dugan JP Coleman CB Haverkos B. Opportunities to target the life cycle of Epstein-Barr virus (EBV) in EBV-associated lymphoproliferative disorders. Front Oncol (2019) 9:127. doi: 10.3389/fonc.2019.00127 30931253 PMC6428703

[31] Iizasa H Nanbo A Nishikawa J Jinushi M Yoshiyama H. Epstein-Barr Virus (EBV)-associated gastric carcinoma. Viruses (2012) 4:3420–39. doi: 10.3390/v4123420 PMC3528272 23342366

[32] Kenney SC. Reactivation and lytic replication of EBV. In: Arvin A Campadelli-Fiume G Mocarski E Moore PS Roizman B Whitley R Yamanishi K, editors. Human herpesviruses: Biology, therapy, and immunoprophylaxis. Cambridge: Cambridge University Press (2007). 21348071

[33] zur Hausen H O’Neill FJ Freese UK Hecker E. Persisting oncogenic herpesvirus induced by the tumour promotor TPA. Nature (1978) 272:373–5. doi: 10.1038/272373a0 204874

[34] Faggioni A Zompetta C Grimaldi S Barile G Frati L Lazdins J. Calcium modulation activates Epstein-Barr virus genome in latently infected cells. Science (1986) 232:1554–6. doi: 10.1126/science.3012779 3012779

[35] Ambinder RF Robertson KD Moore SM Yang J. Epstein-Barr Virus as a therapeutic target in hodgkin’s disease and nasopharyngeal carcinoma. Semin Cancer Biol (1996) 7:217–26. doi: 10.1006/scbi.1996.0029 8946606

[36] Packham G Economou A Rooney CM Rowe DT Farrell PJ. Structure and function of the Epstein-Barr virus BZLF1 protein. J Virol (1990) 64:2110–6. doi: 10.1128/jvi.64.5.2110-2116.1990 PMC249368 2157874

[37] Bhende PM Seaman WT Delecluse HJ Kenney SC. The EBV lytic switch protein, z, preferentially binds to and activates the methylated viral genome. Nat Genet (2004) 36:1099–104. doi: 10.1038/ng1424 15361873

[38] Woellmer A Arteaga-Salas JM Hammerschmidt W. BZLF1 governs CpG-methylated chromatin of Epstein-Barr virus reversing epigenetic repression. PloS Pathog (2012) 8:e1002902. doi: 10.1371/journal.ppat.1002902 22969425 PMC3435241

[39] Tsurumi T Fujita M Kudoh A. Latent and lytic Epstein-Barr virus replication strategies. Rev Med Virol (2005) 15:3–15. doi: 10.1002/rmv.441 15386591

[40] Darr CD Mauser A Kenney S. Epstein-Barr Virus immediate-early protein BRLF1 induces the lytic form of viral replication through a mechanism involving phosphatidylinositol-3 kinase activation. J Virol (2001) 75:6135–42. doi: 10.1128/JVI.75.13.6135-6142.2001 PMC114329 11390615

[41] Lee YH Chiu YF Wang WH Chang LK Liu ST. Activation of the ERK signal transduction pathway by Epstein-Barr virus immediate-early protein rta. J Gen Virol (2008) 89:2437–46. doi: 10.1099/vir.0.2008/003897-0 18796711

[42] Wille CK Nawandar DM Panfil AR Ko MM Hagemeier SR Kenney SC. Viral genome methylation differentially affects the ability of BZLF1 versus BRLF1 to activate Epstein-Barr virus lytic gene expression and viral replication. J Virol (2013) 87:935–50. doi: 10.1128/JVI.01790-12 PMC3554042 23135711

[43] Li D Fu W Swaminathan S. Continuous DNA replication is required for late gene transcription and maintenance of replication compartments in gammaherpesviruses. PloS Pathog (2018) 14:e1007070. doi: 10.1371/journal.ppat.1007070 29813138 PMC5993329

[44] Dyson PJ Farrell PJ. Chromatin structure of Epstein-Barr virus. J Gen Virol (1985) 66(Pt 9):1931–40. doi: 10.1099/0022-1317-66-9-1931 2993484

[45] Avolio-Hunter TM Lewis PN Frappier L. Epstein-Barr Nuclear antigen 1 binds and destabilizes nucleosomes at the viral origin of latent DNA replication. Nucleic Acids Res (2001) 29:3520–8. doi: 10.1093/nar/29.17.3520 PMC55891 11522821

[46] Gruffat H Manet E Sergeant A. MEF2-mediated recruitment of class II HDAC at the EBV immediate early gene BZLF1 links latency and chromatin remodeling. EMBO Rep (2002) 3:141–6. doi: 10.1093/embo-reports/kvf031 PMC1083972 11818339

[47] Hsieh CL. Evidence that protein binding specifies sites of DNA demethylation. Mol Cell Biol (1999) 19:46–56. doi: 10.1128/MCB.19.1.46 9858530 PMC83864

[48] Alazard N Gruffat H Hiriart E Sergeant A Manet E. Differential hyperacetylation of histones H3 and H4 upon promoter-specific recruitment of EBNA2 in Epstein-Barr virus chromatin. J Virol (2003) 77:8166–72. doi: 10.1128/JVI.77.14.8166-8172.2003 PMC161941 12829856

[49] Saemundsen AK Kallin B Klein G. Effect of n-butyrate on cellular and viral DNA synthesis in cells latently infected with Epstein-Barr virus. Virology (1980) 107:557–61. doi: 10.1016/0042-6822(80)90326-8 6256952

[50] Jenkins PJ Binne UK Farrell PJ. Histone acetylation and reactivation of Epstein-Barr virus from latency. J Virol (2000) 74:710–20. doi: 10.1128/JVI.74.2.710-720.2000 PMC111591 10623733

[51] Shaw JE Levinger LF Carter CW Jr. Nucleosomal structure of Epstein-Barr virus DNA in transformed cell lines. J Virol (1979) 29:657–65. doi: 10.1128/jvi.29.2.657-665.1979 PMC353198 219253

[52] Bird AP. CpG-rich islands and the function of DNA methylation. Nature (1986) 321:209–13. doi: 10.1038/321209a0 2423876

[53] Jones PL Wolffe AP. Relationships between chromatin organization and DNA methylation in determining gene expression. Semin Cancer Biol (1999) 9:339–47. doi: 10.1006/scbi.1999.0134 10547342

[54] Diala ES Hoffman RM. Epstein-Barr HR-1 virion DNA is very highly methylated. J Virol (1983) 45:482–3. doi: 10.1128/jvi.45.1.482-483.1983 PMC256435 6296457

[55] Lieberman PM Hu J Renne R. Maintenance and replication during latency. In: Arvin A Campadelli-Fiume G Mocarski E Moore PS Roizman B Whitley R Yamanishi K, editors. Human herpesviruses: Biology, therapy, and immunoprophylaxis. Cambridge: Cambridge University Press (2007). 21348071

[56] Ben-Sasson SA Klein G. Activation of the Epstein-Barr virus genome by 5-aza-cytidine in latently infected human lymphoid lines. Int J Cancer (1981) 28:131–5. doi: 10.1002/ijc.2910280204 6172387

[57] Saha A Robertson ES. Epstein-Barr Virus-associated b-cell lymphomas: pathogenesis and clinical outcomes. Clin Cancer Res (2011) 17:3056–63. doi: 10.1158/1078-0432.CCR-10-2578 PMC4287361 21372216

[58] Hutt-Fletcher LM. Epstein-Barr Virus entry. J Virol (2007) 81:7825–32. doi: 10.1128/JVI.00445-07 PMC1951282 17459936

[59] Hutt-Fletcher LM. Epstein-Barr Virus replicating in epithelial cells. Proc Natl Acad Sci U.S.A. (2014) 111:16242–3. doi: 10.1073/pnas.1418974111 PMC4246291 25385596

[60] Neuhierl B Feederle R Hammerschmidt W Delecluse HJ. Glycoprotein gp110 of Epstein-Barr virus determines viral tropism and efficiency of infection. Proc Natl Acad Sci U.S.A. (2002) 99:15036–41. doi: 10.1073/pnas.232381299 PMC137540 12409611

[61] Shannon-Lowe C Rowe M. Epstein Barr Virus entry; kissing and conjugation. Curr Opin Virol (2014) 4:78–84. doi: 10.1016/j.coviro.2013.12.001 24553068

[62] Chandran B Hutt-Fletcher L. Gammaherpesviruses entry and early events during infection. In: Arvin A Campadelli-Fiume G Mocarski E Moore PS Roizman B Whitley R Yamanishi K, editors. Human herpesviruses: Biology, therapy, and immunoprophylaxis. Cambridge: Cambridge University Press (2007). 21348095

[63] Isobe Y Sugimoto K Yang L Tamayose K Egashira M Kaneko T. Epstein-Barr Virus infection of human natural killer cell lines and peripheral blood natural killer cells. Cancer Res (2004) 64:2167–74. doi: 10.1158/0008-5472.CAN-03-1562 15026359

[64] Smith NA Coleman CB Gewurz BE Rochford R. CD21 (Complement receptor 2) is the receptor for Epstein-Barr virus entry into T cells. J Virol (2020) 94(11):e00428–20. doi: 10.1128/JVI.00428-20 32238579 PMC7269432

[65] Santpere G Darre F Blanco S Alcami A Villoslada P Mar Alba M. Genome-wide analysis of wild-type Epstein-Barr virus genomes derived from healthy individuals of the 1,000 genomes project. Genome Biol Evol (2014) 6:846–60. doi: 10.1093/gbe/evu054 PMC4104767 24682154

[66] Bouvard V Baan R Straif K Grosse Y Secretan B El Ghissassi F. A review of human carcinogens–part b: biological agents. Lancet Oncol (2009) 10:321–2. doi: 10.1016/S1470-2045(09)70096-8 19350698

[67] Zheng X Huang Y Li K Luo R Cai M Yun J. Immunosuppressive tumor microenvironment and immunotherapy of Epstein-Barr virus-associated malignancies. Viruses (2022) 14(5):1017. doi: 10.3390/v14051017 35632758 PMC9146158

[68] Bridges R Correia S Wegner F Venturini C Palser A White RE. Essential role of inverted repeat in Epstein-Barr virus IR-1 in b cell transformation; geographical variation of the viral genome. Philos Trans R Soc Lond B Biol Sci (2019) 374:20180299. doi: 10.1098/rstb.2018.0299 30955492 PMC6501908

[69] Chesnokova LS Valencia SM Hutt-Fletcher LM. The BDLF3 gene product of Epstein-Barr virus, gp150, mediates non-productive binding to heparan sulfate on epithelial cells and only the binding domain of CD21 is required for infection. Virology (2016) 494:23–8. doi: 10.1016/j.virol.2016.04.002 PMC4884462 27061054

[70] Zhang H Li Y Wang HB Zhang A Chen ML Fang ZX. Ephrin receptor A2 is an epithelial cell receptor for Epstein-Barr virus entry. Nat Microbiol (2018) 3:1–8. doi: 10.1038/s41564-017-0080-8 29292383

[71] Lee JH Choi J Ahn YO Kim TM Heo DS. CD21-independent Epstein-Barr virus entry into NK cells. Cell Immunol (2018) 327:21–5. doi: 10.1016/j.cellimm.2018.01.011 29499908

[72] Kintner CR Sugden B. The structure of the termini of the DNA of Epstein-Barr virus. Cell (1979) 17:661–71. doi: 10.1016/0092-8674(79)90273-3 225039

[73] Cheung RK Miyazaki I Dosch HM. Unexpected patterns of Epstein-Barr virus gene expression during early stages of b cell transformation. Int Immunol (1993) 5:707–16. doi: 10.1093/intimm/5.7.707 8396414

[74] Hurley EA Thorley-Lawson DA. B cell activation and the establishment of Epstein-Barr virus latency. J Exp Med (1988) 168:2059–75. doi: 10.1084/jem.168.6.2059 PMC2189139 2848918

[75] Kieff E Rickinson AB. Epstein-Barr Virus and its replication. fields virology, 4th ed. Philadelphia: Lippincott Williams & Wilkins (2001). pp. 2511–73.

[76] Morgan SM Tanizawa H Caruso LB Hulse M Kossenkov A Madzo J. The three-dimensional structure of Epstein-Barr virus genome varies by latency type and is regulated by PARP1 enzymatic activity. Nat Commun (2022) 13:187. doi: 10.1038/s41467-021-27894-1 35039491 PMC8764100

[77] Sample J Young L Martin B Chatman T Kieff E Rickinson A. Epstein-Barr Virus types 1 and 2 differ in their EBNA-3A, EBNA-3B, and EBNA-3C genes. J Virol (1990) 64:4084–92. doi: 10.1128/jvi.64.9.4084-4092.1990 PMC247870 2166806

[78] Dambaugh T Hennessy K Chamnankit L Kieff E. U2 region of Epstein-Barr virus DNA may encode Epstein-Barr nuclear antigen 2. Proc Natl Acad Sci U.S.A. (1984) 81:7632–6. doi: 10.1073/pnas.81.23.7632 PMC392202 6209719

[79] Kanda T Yajima M Ikuta K. Epstein-Barr Virus strain variation and cancer. Cancer Sci (2019) 110:1132–9. doi: 10.1111/cas.13954 PMC6447851 30697862

[80] Tsai MH Raykova A Klinke O Bernhardt K Gartner K Leung CS. Spontaneous lytic replication and epitheliotropism define an Epstein-Barr virus strain found in carcinomas. Cell Rep (2013) 5:458–70. doi: 10.1016/j.celrep.2013.09.012 24120866

[81] Bristol JA Djavadian R Albright ER Coleman CB Ohashi M Hayes M. A cancer-associated Epstein-Barr virus BZLF1 promoter variant enhances lytic infection. PloS Pathog (2018) 14:e1007179. doi: 10.1371/journal.ppat.1007179 30052684 PMC6082571

[82] Yates J Warren N Reisman D Sugden B. A cis-acting element from the Epstein-Barr viral genome that permits stable replication of recombinant plasmids in latently infected cells. Proc Natl Acad Sci U.S.A. (1984) 81:3806–10. doi: 10.1073/pnas.81.12.3806 PMC345309 6328526

[83] Yates JL Warren N Sugden B. Stable replication of plasmids derived from Epstein-Barr virus in various mammalian cells. Nature (1985) 313:812–5. doi: 10.1038/313812a0 2983224

[84] Lee MA Diamond ME Yates JL. Genetic evidence that EBNA-1 is needed for efficient, stable latent infection by Epstein-Barr virus. J Virol (1999) 73:2974–82. doi: 10.1128/JVI.73.4.2974-2982.1999 PMC104057 10074147

[85] Frappier L. The Epstein-Barr virus EBNA1 protein. Scientifica (Cairo) (2012) 2012:438204. doi: 10.6064/2012/438204 24278697 PMC3820569

[86] Wu H Kapoor P Frappier L. Separation of the DNA replication, segregation, and transcriptional activation functions of Epstein-Barr nuclear antigen 1. J Virol (2002) 76:2480–90. doi: 10.1128/jvi.76.5.2480-2490.2002 PMC135949 11836426

[87] Sugden B Warren N. A promoter of Epstein-Barr virus that can function during latent infection can be transactivated by EBNA-1, a viral protein required for viral DNA replication during latent infection. J Virol (1989) 63:2644–9. doi: 10.1128/jvi.63.6.2644-2649.1989 PMC250748 2542577

[88] Wysokenski DA Yates JL. Multiple EBNA1-binding sites are required to form an EBNA1-dependent enhancer and to activate a minimal replicative origin within oriP of Epstein-Barr virus. J Virol (1989) 63:2657–66. doi: 10.1128/jvi.63.6.2657-2666.1989 PMC250751 2542579

[89] Sung NS Wilson J Davenport M Sista ND Pagano JS. Reciprocal regulation of the Epstein-Barr virus BamHI-f promoter by EBNA-1 and an E2F transcription factor. Mol Cell Biol (1994) 14:7144–52. doi: 10.1128/mcb.14.11.7144-7152.1994 PMC359248 7935429

[90] Humme S Reisbach G Feederle R Delecluse HJ Bousset K Hammerschmidt W. The EBV nuclear antigen 1 (EBNA1) enhances b cell immortalization several thousandfold. Proc Natl Acad Sci USA (2003) 100:10989–94. doi: 10.1073/pnas.1832776100 PMC196914 12947043

[91] Kennedy G Komano J Sugden B. Epstein-Barr Virus provides a survival factor to burkitt’s lymphomas. Proc Natl Acad Sci USA (2003) 100:14269–74. doi: 10.1073/pnas.2336099100 PMC283581 14603034

[92] Wood VH O’Neil JD Wei W Stewart SE Dawson CW Young LS. Epstein-Barr Virus-encoded EBNA1 regulates cellular gene transcription and modulates the STAT1 and TGFbeta signaling pathways. Oncogene (2007) 26:4135–47. doi: 10.1038/sj.onc.1210496 17486072

[93] Flavell JR Baumforth KR Wood VH Davies GL Wei W Reynolds GM. Down-regulation of the TGF-beta target gene, PTPRK, by the Epstein-Barr virus encoded EBNA1 contributes to the growth and survival of Hodgkin lymphoma cells. Blood (2008) 111:292–301. doi: 10.1182/blood-2006-11-059881 17720884

[94] Valentine R Dawson CW Hu C Shah KM Owen TJ Date KL. Epstein-Barr Virus-encoded EBNA1 inhibits the canonical NF-kappaB pathway in carcinoma cells by inhibiting IKK phosphorylation. Mol Cancer (2010) 9:1. doi: 10.1186/1476-4598-9-1 20051109 PMC2818691

[95] Cao JY Mansouri S Frappier L. Changes in the nasopharyngeal carcinoma nuclear proteome induced by the EBNA1 protein of Epstein-Barr virus reveal potential roles for EBNA1 in metastasis and oxidative stress responses. J Virol (2012) 86:382–94. doi: 10.1128/JVI.05648-11 PMC3255880 22013061

[96] Sivachandran N Dawson CW Young LS Liu FF Middeldorp J Frappier L. Contributions of the Epstein-Barr virus EBNA1 protein to gastric carcinoma. J Virol (2012) 86:60–8. doi: 10.1128/JVI.05623-11 PMC3255905 22013060

[97] Soldan SS Anderson EM Frase DM Zhang Y Caruso LB Wang Y. EBNA1 inhibitors have potent and selective antitumor activity in xenograft models of Epstein-Barr virus-associated gastric cancer. Gastric Cancer (2021) 24:1076–88. doi: 10.1007/s10120-021-01193-6 PMC8338878 33929613

[98] Wang F Gregory C Sample C Rowe M Liebowitz D Murray R. Epstein-Barr Virus latent membrane protein (LMP1) and nuclear proteins 2 and 3C are effectors of phenotypic changes in b lymphocytes: EBNA-2 and LMP1 cooperatively induce CD23. J Virol (1990) 64:2309–18. doi: 10.1128/jvi.64.5.2309-2318.1990 PMC249392 2157887

[99] Kieser A Kilger E Gires O Ueffing M Kolch W Hammerschmidt W. Epstein-Barr Virus latent membrane protein-1 triggers AP-1 activity *via* the c-jun n-terminal kinase cascade. EMBO J (1997) 16:6478–85. doi: 10.1093/emboj/16.21.6478 PMC1170253 9351829

[100] Maier S Staffler G Hartmann A Hock J Henning K Grabusic K. Cellular target genes of Epstein-Barr virus nuclear antigen 2. J Virol (2006) 80:9761–71. doi: 10.1128/JVI.00665-06 PMC1617228 16973580

[101] Zhao B Mar JC Maruo S Lee S Gewurz BE Johannsen E. Epstein-Barr Virus nuclear antigen 3C regulated genes in lymphoblastoid cell lines. Proc Natl Acad Sci USA (2011) 108:337–42. doi: 10.1073/pnas.1017419108 PMC3017193 21173222

[102] Johannsen E Koh E Mosialos G Tong X Kieff E Grossman SR. Epstein-Barr Virus nuclear protein 2 transactivation of the latent membrane protein 1 promoter is mediated by J kappa and PU.1. J Virol (1995) 69:253–62. doi: 10.1128/jvi.69.1.253-262.1995 PMC188571 7983717

[103] Hofelmayr H Strobl LJ Marschall G Bornkamm GW Zimber-Strobl U. Activated Notch1 can transiently substitute for EBNA2 in the maintenance of proliferation of LMP1-expressing immortalized b cells. J Virol (2001) 75:2033–40. doi: 10.1128/JVI.75.5.2033-2040.2001 PMC114787 11160707

[104] Strobl LJ Hofelmayr H Marschall G Brielmeier M Bornkamm GW Zimber-Strobl U. Activated Notch1 modulates gene expression in b cells similarly to Epstein-Barr viral nuclear antigen 2. J Virol (2000) 74:1727–35. doi: 10.1128/JVI.74.4.1727-1735.2000 PMC111648 10644343

[105] Harada S Kieff E. Epstein-Barr Virus nuclear protein LP stimulates EBNA-2 acidic domain-mediated transcriptional activation. J Virol (1997) 71:6611–8. doi: 10.1128/jvi.71.9.6611-6618.1997 PMC191939 9261383

[106] Wood CD Veenstra H Khasnis S Gunnell A Webb HM Shannon-Lowe C. MYC activation and BCL2L11 silencing by a tumour virus through the large-scale reconfiguration of enhancer-promoter hubs. Elife (2016) 5. doi: 10.7554/eLife.18270 PMC5005034 27490482

[107] Touitou R O’Nions J Heaney J Allday MJ. Epstein-Barr Virus EBNA3 proteins bind to the C8/alpha7 subunit of the 20S proteasome and are degraded by 20S proteasomes *in vitro*, but are very stable in latently infected b cells. J Gen Virol (2005) 86:1269–77. doi: 10.1099/vir.0.80763-0 15831937

[108] White RE Groves IJ Turro E Yee J Kremmer E Allday MJ. Extensive co-operation between the Epstein-Barr virus EBNA3 proteins in the manipulation of host gene expression and epigenetic chromatin modification. PloS One (2010) 5:e13979. doi: 10.1371/journal.pone.0013979 21085583 PMC2981562

[109] Hertle ML Popp C Petermann S Maier S Kremmer E Lang R. Differential gene expression patterns of EBV infected EBNA-3A positive and negative human b lymphocytes. PloS Pathog (2009) 5:e1000506. doi: 10.1371/journal.ppat.1000506 19578441 PMC2700271

[110] Paschos K Bazot Q Ho G Parker GA Lees J Barton G. Core binding factor (CBF) is required for Epstein-Barr virus EBNA3 proteins to regulate target gene expression. Nucleic Acids Res (2017) 45:2368–83. doi: 10.1093/nar/gkw1167 PMC5389572 27903901

[111] Davies ML Xu S Lyons-Weiler J Rosendorff A Webber SA Wasil LR. Cellular factors associated with latency and spontaneous Epstein-Barr virus reactivation in b-lymphoblastoid cell lines. Virology (2010) 400:53–67. doi: 10.1016/j.virol.2010.01.002 20153012

[112] Robertson ES Lin J Kieff E. The amino-terminal domains of Epstein-Barr virus nuclear proteins 3A, 3B, and 3C interact with RBPJ(kappa). J Virol (1996) 70:3068–74. doi: 10.1128/jvi.70.5.3068-3074.1996 PMC190168 8627785

[113] Robertson ES Grossman S Johannsen E Miller C Lin J Tomkinson B. Epstein-Barr Virus nuclear protein 3C modulates transcription through interaction with the sequence-specific DNA-binding protein J kappa. J Virol (1995) 69:3108–16. doi: 10.1128/jvi.69.5.3108-3116.1995 PMC189012 7707539

[114] Johannsen E Miller CL Grossman SR Kieff E. EBNA-2 and EBNA-3C extensively and mutually exclusively associate with RBPJkappa in Epstein-Barr virus-transformed b lymphocytes. J Virol (1996) 70:4179–83. doi: 10.1128/jvi.70.6.4179-4183.1996 PMC190314 8648764

[115] Murer A McHugh D Caduff N Kalchschmidt J Barros M Zbinden A. EBV persistence without its EBNA3A and 3C oncogenes *in vivo* . PloS Pathog (2018) 14:e1007039. doi: 10.1371/journal.ppat.1007039 29709016 PMC5945050

[116] West MJ. Chromatin reorganisation in Epstein-Barr virus-infected cells and its role in cancer development. Curr Opin Virol (2017) 26:149–55. doi: 10.1016/j.coviro.2017.08.004 28910751

[117] Huen DS Henderson SA Croom-Carter D Rowe M. The Epstein-Barr virus latent membrane protein-1 (LMP1) mediates activation of NF-kappa b and cell surface phenotype *via* two effector regions in its carboxy-terminal cytoplasmic domain. Oncogene (1995) 10:549–60. 7845680

[118] Uchida J Yasui T Takaoka-Shichijo Y Muraoka M Kulwichit W Raab-Traub N. Mimicry of CD40 signals by Epstein-Barr virus LMP1 in b lymphocyte responses. Science (1999) 286:300–3. doi: 10.1126/science.286.5438.300 10514374

[119] Soni V Cahir-McFarland E Kieff E. LMP1 TRAFficking activates growth and survival pathways. Adv Exp Med Biol (2007) 597:173–87. doi: 10.1007/978-0-387-70630-6_14 17633026

[120] Cheerathodi MR Meckes DG Jr. The Epstein-Barr virus LMP1 interactome: biological implications and therapeutic targets. Future Virol (2018) 13:863–87. doi: 10.2217/fvl-2018-0120 PMC8168621 34079586

[121] Gires O Zimber-Strobl U Gonnella R Ueffing M Marschall G Zeidler R. Latent membrane protein 1 of Epstein-Barr virus mimics a constitutively active receptor molecule. EMBO J (1997) 16:6131–40. doi: 10.1093/emboj/16.20.6131 PMC1326297 9359753

[122] Kilger E Kieser A Baumann M Hammerschmidt W. Epstein-Barr Virus-mediated b-cell proliferation is dependent upon latent membrane protein 1, which simulates an activated CD40 receptor. EMBO J (1998) 17:1700–9. doi: 10.1093/emboj/17.6.1700 PMC1170517 9501091

[123] Dawson CW Tramountanis G Eliopoulos AG Young LS. Epstein-Barr Virus latent membrane protein 1 (LMP1) activates the phosphatidylinositol 3-kinase/Akt pathway to promote cell survival and induce actin filament remodeling. J Biol Chem (2003) 278:3694–704. doi: 10.1074/jbc.M209840200 12446712

[124] Eliopoulos AG Stack M Dawson CW Kaye KM Hodgkin L Sihota S. Epstein-Barr Virus-encoded LMP1 and CD40 mediate IL-6 production in epithelial cells *via* an NF-kappaB pathway involving TNF receptor-associated factors. Oncogene (1997) 14:2899–916. doi: 10.1038/sj.onc.1201258 9205097

[125] Laherty CD Hu HM Opipari AW Wang F Dixit VM. The Epstein-Barr virus LMP1 gene product induces A20 zinc finger protein expression by activating nuclear factor kappa b. J Biol Chem (1992) 267:24157–60. doi: 10.1016/S0021-9258(18)35741-7 1332946

[126] Henderson S Rowe M Gregory C Croom-Carter D Wang F Longnecker R. Induction of bcl-2 expression by Epstein-Barr virus latent membrane protein 1 protects infected b cells from programmed cell death. Cell (1991) 65:1107–15. doi: 10.1016/0092-8674(91)90007-L 1648447

[127] Fruehling S Longnecker R. The immunoreceptor tyrosine-based activation motif of Epstein-Barr virus LMP2A is essential for blocking BCR-mediated signal transduction. Virology (1997) 235:241–51. doi: 10.1006/viro.1997.8690 9281504

[128] Miller CL Burkhardt AL Lee JH Stealey B Longnecker R Bolen JB. Integral membrane protein 2 of Epstein-Barr virus regulates reactivation from latency through dominant negative effects on protein-tyrosine kinases. Immunity (1995) 2:155–66. doi: 10.1016/S1074-7613(95)80040-9 7895172

[129] Longnecker R. Epstein-Barr Virus latency: LMP2, a regulator or means for Epstein-Barr virus persistence? Adv Cancer Res (2000) 79:175–200. doi: 10.1016/S0065-230X(00)79006-3 10818681

[130] Pan Y Zhang J Zhou L Zuo J Zeng Y. *In vitro* anti-tumor immune response induced by dendritic cells transfected with EBV-LMP2 recombinant adenovirus. Biochem Biophys Res Commun (2006) 347:551–7. doi: 10.1016/j.bbrc.2006.05.214 16842756

[131] Hayashi T Horiuchi A Sano K Hiraoka N Kasai M Ichimura T. Potential role of LMP2 as tumor-suppressor defines new targets for uterine leiomyosarcoma therapy. Sci Rep (2011) 1:180. doi: 10.1038/srep00180 22355695 PMC3240965

[132] Swaminathan S Tomkinson B Kieff E. Recombinant Epstein-Barr virus with small RNA (EBER) genes deleted transforms lymphocytes and replicates *in vitro* . Proc Natl Acad Sci USA (1991) 88:1546–50. doi: 10.1073/pnas.88.4.1546 PMC51056 1847527

[133] Samanta M Iwakiri D Takada K. Epstein-Barr Virus-encoded small RNA induces IL-10 through RIG-i-mediated IRF-3 signaling. Oncogene (2008) 27:4150–60. doi: 10.1038/onc.2008.75 18362887

[134] Tsimbouri P Al-Sheikh Y Drotar ME Cushley W Wilson JB. Epstein-Barr Virus nuclear antigen-1 renders lymphocytes responsive to IL-2 but not IL-15 for survival. J Gen Virol (2008) 89:2821–32. doi: 10.1099/vir.0.83296-0 18931080

[135] Pfeffer S Zavolan M Grasser FA Chien M Russo JJ Ju J. Identification of virus-encoded microRNAs. Science (2004) 304:734–6. doi: 10.1126/science.1096781 15118162

[136] Grundhoff A Sullivan CS Ganem D. A combined computational and microarray-based approach identifies novel microRNAs encoded by human gamma-herpesviruses. RNA (2006) 12:733–50. doi: 10.1261/rna.2326106 PMC1440911 16540699

[137] Cai X Schafer A Lu S Bilello JP Desrosiers RC Edwards R. Epstein-Barr Virus microRNAs are evolutionarily conserved and differentially expressed. PloS Pathog (2006) 2:e23. doi: 10.1371/journal.ppat.0020023 16557291 PMC1409806

[138] Sakamoto Y Mariya Y Kubo K. Quantification of Epstein-Barr virus DNA is helpful for evaluation of chronic active Epstein-Barr virus infection. Tohoku J Exp Med (2012) 227:307–11. doi: 10.1620/tjem.227.307 22850617

[139] Kim WY Montes-Mojarro IA Fend F Quintanilla-Martinez L. Epstein-Barr Virus-associated T and NK-cell lymphoproliferative diseases. Front Pediatr (2019) 7:71. doi: 10.3389/fped.2019.00071 30931288 PMC6428722

[140] Sakai Y Ohga S Tonegawa Y Takada H Nakao F Nakayama H. Interferon-alpha therapy for chronic active Epstein-Barr virus infection: potential effect on the development of T-lymphoproliferative disease. J Pediatr Hematol Oncol (1998) 20:342–6. doi: 10.1097/00043426-199807000-00013 9703010

[141] Zhang Q Zhao YZ Ma HH Wang D Cui L Li WJ. A study of ruxolitinib response-based stratified treatment for pediatric hemophagocytic lymphohistiocytosis. Blood (2022) 139:3493–504. doi: 10.1182/blood.2021014860 35344583

[142] Pietersma F Piriou E van Baarle D. Immune surveillance of EBV-infected b cells and the development of non-Hodgkin lymphomas in immunocompromised patients. Leuk Lymphoma (2008) 49:1028–41. doi: 10.1080/10428190801911662 18452077

[143] Sebelin-Wulf K Nguyen TD Oertel S Papp-Vary M Trappe RU Schulzki A. Quantitative analysis of EBV-specific CD4/CD8 T cell numbers, absolute CD4/CD8 T cell numbers and EBV load in solid organ transplant recipients with PLTD. Transpl Immunol (2007) 17:203–10. doi: 10.1016/j.trim.2006.10.006 17331848

[144] Lau R Middeldorp J Farrell PJ. Epstein-Barr Virus gene expression in oral hairy leukoplakia. Virology (1993) 195:463–74. doi: 10.1006/viro.1993.1397 8393235

[145] Walling DM Flaitz CM Nichols CM. Epstein-Barr Virus replication in oral hairy leukoplakia: response, persistence, and resistance to treatment with valacyclovir. J Infect Dis (2003) 188:883–90. doi: 10.1086/378072 12964120

[146] Greenspan JS Greenspan D Lennette ET Abrams DI Conant MA Petersen V. Replication of Epstein-Barr virus within the epithelial cells of oral “hairy”. leukoplakia an AIDS-associated lesion N Engl J Med (1985) 313:1564–71. doi: 10.1056/NEJM198512193132502 2999595

[147] Resnick L Herbst JS Raab-Traub N. Oral hairy leukoplakia. J Am Acad Dermatol (1990) 22:1278–82. doi: 10.1016/0190-9622(90)70174-G 2163409

[148] Kim HJ Ko YH Kim JE Lee SS Lee H Park G. Epstein-Barr Virus-associated lymphoproliferative disorders: Review and update on 2016 WHO classification. J Pathol Transl Med (2017) 51:352–8. doi: 10.4132/jptm.2017.03.15 PMC5525035 28592786

[149] Green M Michaels MG. Epstein-Barr Virus infection and posttransplant lymphoproliferative disorder. Am J Transplant (2013) 13(Suppl 3):41–54; quiz 54. doi: 10.1111/ajt.12004 23347213

[150] Wagner HJ Wessel M Jabs W Smets F Fischer L Offner G. Patients at risk for development of posttransplant lymphoproliferative disorder: plasma versus peripheral blood mononuclear cells as material for quantification of Epstein-Barr viral load by using real-time quantitative polymerase chain reaction. Transplantation (2001) 72:1012–9. doi: 10.1097/00007890-200109270-00006 11579293

[151] Green M. Management of Epstein-Barr virus-induced post-transplant lymphoproliferative disease in recipients of solid organ transplantation. Am J Transplant (2001) 1:103–8. doi: 10.3324/haematol.2016.144428 12099356

[152] Darenkov IA Marcarelli MA Basadonna GP Friedman AL Lorber KM Howe JG. Reduced incidence of Epstein-Barr virus-associated posttransplant lymphoproliferative disorder using preemptive antiviral therapy. Transplantation (1997) 64:848–52. doi: 10.1097/00007890-199709270-00010 9326409

[153] Bar-Or A Pender MP Khanna R Steinman L Hartung HP Maniar T. Epstein-Barr Virus in multiple sclerosis: Theory and emerging immunotherapies. Trends Mol Med (2020) 26:296–310. doi: 10.1016/j.molmed.2019.11.003 31862243 PMC7106557

[154] Bjornevik K Cortese M Healy BC Kuhle J Mina MJ Leng Y. Longitudinal analysis reveals high prevalence of Epstein-Barr virus associated with multiple sclerosis. Science (2022) 375:296–301. doi: 10.1126/science.abj8222 35025605

[155] Lanz TV Brewer RC Ho PP Moon JS Jude KM Fernandez D. Clonally expanded b cells in multiple sclerosis bind EBV EBNA1 and GlialCAM. Nature (2022) 603:321–7. doi: 10.1038/s41586-022-04432-7 PMC9382663 35073561

[156] Miller G Lipman M. Release of infectious Epstein-Barr virus by transformed marmoset leukocytes. Proc Natl Acad Sci U.S.A. (1973) 70:190–4. doi: 10.1073/pnas.70.1.190 PMC433213 4346033

[157] Miller G Lipman M. Comparison of the yield of infectious virus from clones of human and simian lymphoblastoid lines transformed by Epstein-Barr virus. J Exp Med (1973) 138:1398–412. doi: 10.1084/jem.138.6.1398 PMC2139465 4357683

[158] de Martel C Ferlay J Franceschi S Vignat J Bray F Forman D. Global burden of cancers attributable to infections in 2008: a review and synthetic analysis. Lancet Oncol (2012) 13:607–15. doi: 10.1016/S1470-2045(12)70137-7 22575588

[159] Khan G Hashim MJ. Global burden of deaths from Epstein-Barr virus attributable malignancies 1990-2010. Infect Agent Cancer (2014) 9:38. doi: 10.1186/1750-9378-9-38 25473414 PMC4253616

[160] Wong Y Meehan MT Burrows SR Doolan DL Miles JJ. Estimating the global burden of Epstein-Barr virus-related cancers. J Cancer Res Clin Oncol (2022) 148:31–46. doi: 10.1007/s00432-021-03824-y 34705104 PMC8752571

[161] Khan G Fitzmaurice C Naghavi M Ahmed LA. Global and regional incidence, mortality and disability-adjusted life-years for Epstein-Barr virus-attributable malignancies, 1990-2017. BMJ Open (2020) 10:e037505. doi: 10.1136/bmjopen-2020-037505 PMC7462312 32868361

[162] Taylor GS Blackbourn DJ. Infectious agents in human cancers: lessons in immunity and immunomodulation from gammaherpesviruses EBV and KSHV. Cancer Lett (2011) 305:263–78. doi: 10.1016/j.canlet.2010.08.019 21470769

[163] Burkitt D. **A** sarcoma involving the jaws in African children. Br J Surg (1958) 46:218–23. doi: 10.1002/bjs.18004619704 13628987

[164] World Health O. Report of the ninth meeting of the WHO technical advisory group on leprosy control: Cairo, Egypt, 6-7 march 2008. Lepr Rev (2008) 79:452–70. doi: 10.47276/lr.79.4.452 19274996

[165] Ayee R Ofori MEO Wright E Quaye O. Epstein Barr Virus associated lymphomas and epithelia cancers in humans. J Cancer (2020) 11:1737–50. doi: 10.7150/jca.37282 PMC7052849 32194785

[166] Frappier L. Contributions of Epstein-Barr nuclear antigen 1 (EBNA1) to cell immortalization and survival. Viruses (2012) 4:1537–47. doi: 10.3390/v4091537 PMC3499818 23170171

[167] Magrath I. Epidemiology: clues to the pathogenesis of burkitt lymphoma. Br J Haematol (2012) 156:744–56. doi: 10.1111/j.1365-2141.2011.09013.x 22260300

[168] Costa LJ Xavier AC Wahlquist AE Hill EG. Trends in survival of patients with burkitt lymphoma/leukemia in the USA: an analysis of 3691 cases. Blood (2013) 121:4861–6. doi: 10.1182/blood-2012-12-475558 PMC3682339 23641015

[169] Buckle G Maranda L Skiles J Ong’echa JM Foley J Epstein M. Factors influencing survival among Kenyan children diagnosed with endemic burkitt lymphoma between 2003 and 2011: A historical cohort study. Int J Cancer (2016) 139:1231–40. doi: 10.1002/ijc.30170 PMC5489240 27136063

[170] Joko-Fru WY Parkin DM Borok M Chokunonga E Korir A Nambooze S. Survival from childhood cancers in Eastern Africa: A population-based registry study. Int J Cancer (2018) 143:2409–15. doi: 10.1002/ijc.31723 29981149

[171] Mbulaiteye SM Pullarkat ST Nathwani BN Weiss LM Rao N Emmanuel B. Epstein-Barr Virus patterns in US burkitt lymphoma tumors from the SEER residual tissue repository during 1979-2009. APMIS (2014) 122:5–15. doi: 10.1111/apm.12078 23607450 PMC3723754

[172] Guikema JE Schuuring E Kluin PM. Structure and consequences of IGH switch breakpoints in burkitt lymphoma. J Natl Cancer Inst Monogr (2008) 39:32–6. doi: 10.1093/jncimonographs/lgn020:32-6 18647999

[173] Allday MJ. How does Epstein-Barr virus (EBV) complement the activation of myc in the pathogenesis of burkitt’s lymphoma? Semin Cancer Biol (2009) 19:366–76. doi: 10.1016/j.semcancer.2009.07.007 PMC3770905 19635566

[174] Schmitz R Ceribelli M Pittaluga S Wright G Staudt LM. Oncogenic mechanisms in burkitt lymphoma. Cold Spring Harb Perspect Med (2014) 4(2):a014282. doi: 10.1101/cshperspect.a014282 24492847 PMC3904095

[175] Giulino-Roth L Wang K MacDonald TY Mathew S Tam Y Cronin MT. Targeted genomic sequencing of pediatric burkitt lymphoma identifies recurrent alterations in antiapoptotic and chromatin-remodeling genes. Blood (2012) 120:5181–4. doi: 10.1182/blood-2012-06-437624 PMC3537311 23091298

[176] Schmitz R Young RM Ceribelli M Jhavar S Xiao W Zhang M. Burkitt lymphoma pathogenesis and therapeutic targets from structural and functional genomics. Nature (2012) 490:116–20. doi: 10.1038/nature11378 PMC3609867 22885699

[177] Love C Sun Z Jima D Li G Zhang J Miles R. The genetic landscape of mutations in burkitt lymphoma. Nat Genet (2012) 44:1321–5. doi: 10.1038/ng.2468 PMC3674561 23143597

[178] Rohde M Bonn BR Zimmermann M Lange J Moricke A Klapper W. Relevance of ID3-TCF3-CCND3 pathway mutations in pediatric aggressive b-cell lymphoma treated according to the non-Hodgkin lymphoma Berlin-Frankfurt-Munster protocols. Haematologica (2017) 102:1091–8. doi: 10.3324/haematol.2016.156885 PMC5451341 28209658

[179] Abate F Ambrosio MR Mundo L Laginestra MA Fuligni F Rossi M. Distinct viral and mutational spectrum of endemic burkitt lymphoma. PloS Pathog (2015) 11:e1005158. doi: 10.1371/journal.ppat.1005158 26468873 PMC4607508

[180] Chakravorty S Yan B Wang C Wang L Quaid JT Lin CF. Integrated pan-cancer map of EBV-associated neoplasms reveals functional host-virus interactions. Cancer Res (2019) 79:6010–23. doi: 10.1158/0008-5472.CAN-19-0615 PMC6891172 31481499

[181] Fish K Chen J Longnecker R. Epstein-Barr Virus latent membrane protein 2A enhances MYC-driven cell cycle progression in a mouse model of b lymphoma. Blood (2014) 123:530–40. doi: 10.1182/blood-2013-07-517649 PMC3901066 24174629

[182] Dittmer DP. Not like a wrecking ball: EBV fine-tunes MYC lymphomagenesis. Blood (2014) 123:460–1. doi: 10.1182/blood-2013-11-537076 PMC3901062 24458272

[183] Sander S Calado DP Srinivasan L Kochert K Zhang B Rosolowski M. Synergy between PI3K signaling and MYC in burkitt lymphomagenesis. Cancer Cell (2012) 22:167–79. doi: 10.1016/j.ccr.2012.06.012 PMC3432451 22897848

[184] Ikeda M Hayes CK Schaller SJ Longnecker R. Latent membrane proteins from EBV differentially target cellular pathways to accelerate MYC-induced lymphomagenesis. Blood Adv (2022) 6:4283–96. doi: 10.1182/bloodadvances.2022007695 PMC9327557 35605249

[185] Kaymaz Y Oduor CI Yu H Otieno JA Ong’echa JM Moormann AM. Comprehensive transcriptome and mutational profiling of endemic burkitt lymphoma reveals EBV type-specific differences. Mol Cancer Res (2017) 15:563–76. doi: 10.1158/1541-7786.MCR-16-0305 PMC5471630 28465297

[186] Grande BM Gerhard DS Jiang A Griner NB Abramson JS Alexander TB. Genome-wide discovery of somatic coding and noncoding mutations in pediatric endemic and sporadic burkitt lymphoma. Blood (2019) 133:1313–24. doi: 10.1182/blood-2018-09-871418 PMC6428665 30617194

[187] Kalchschmidt JS Bashford-Rogers R Paschos K Gillman AC Styles CT Kellam P. Epstein-Barr Virus nuclear protein EBNA3C directly induces expression of AID and somatic mutations in b cells. J Exp Med (2016) 213:921–8. doi: 10.1084/jem.20160120 PMC4886369 27217538

[188] Takizawa M Tolarova H Li Z Dubois W Lim S Callen E. AID expression levels determine the extent of cMyc oncogenic translocations and the incidence of b cell tumor development. J Exp Med (2008) 205:1949–57. doi: 10.1084/jem.20081007 PMC2526190 18678733

[189] Thorley-Lawson D Deitsch KW Duca KA Torgbor C. The link between plasmodium falciparum malaria and endemic burkitt’s lymphoma-new insight into a 50-Year-Old enigma. PloS Pathog (2016) 12:e1005331. doi: 10.1371/journal.ppat.1005331 26794909 PMC4721646

[190] Carbone A Cesarman E Spina M Gloghini A Schulz TF. HIV-Associated lymphomas and gamma-herpesviruses. Blood (2009) 113:1213–24. doi: 10.1182/blood-2008-09-180315 18955561

[191] Shannon-Lowe C Rickinson A. The global landscape of EBV-associated tumors. Front Oncol (2019) 9:713. doi: 10.3389/fonc.2019.00713 31448229 PMC6691157

[192] Vrzalikova K Ibrahim M Nagy E Vockerodt M Perry T Wei W. Co-Expression of the Epstein-Barr virus-encoded latent membrane proteins and the pathogenesis of classic Hodgkin lymphoma. Cancers (Basel) (2018) 10(9):285. doi: 10.3390/cancers10090285 PMC6162670 30149502

[193] Lake A Shield LA Cordano P Chui DT Osborne J Crae S. Mutations of NFKBIA, encoding IkappaB alpha, are a recurrent finding in classical Hodgkin lymphoma but are not a unifying feature of non-EBV-associated cases. Int J Cancer (2009) 125:1334–42. doi: 10.1002/ijc.24502 19507254

[194] Kuppers R Engert A Hansmann ML. Hodgkin Lymphoma. J Clin Invest (2012) 122:3439–47. doi: 10.1172/JCI61245 PMC3534167 23023715

[195] Weniger MA Kuppers R. Molecular biology of Hodgkin lymphoma. Leukemia (2021) 35:968–81. doi: 10.1038/s41375-021-01204-6 PMC8024192 33686198

[196] Wu TC Mann RB Charache P Hayward SD Staal S Lambe BC. Detection of EBV gene expression in reed-sternberg cells of hodgkin’s disease. Int J Cancer (1990) 46:801–4. doi: 10.1002/ijc.2910460509 2172169

[197] Shannon-Lowe C Rickinson AB Bell AI. Epstein-Barr Virus-associated lymphomas. Philos Trans R Soc Lond B Biol Sci (2017) 372(1732). doi: 10.1098/rstb.2016.0271 PMC5597738 28893938

[198] Bargou RC Emmerich F Krappmann D Bommert K Mapara MY Arnold W. Constitutive nuclear factor-kappaB-RelA activation is required for proliferation and survival of hodgkin’s disease tumor cells. J Clin Invest (1997) 100:2961–9. doi: 10.1172/JCI119849 PMC508507 9399941

[199] Kube D Holtick U Vockerodt M Ahmadi T Haier B Behrmann I. STAT3 is constitutively activated in Hodgkin cell lines. Blood (2001) 98:762–70. doi: 10.1182/blood.V98.3.762 11468177

[200] Skinnider BF Elia AJ Gascoyne RD Patterson B Trumper L Kapp U. Signal transducer and activator of transcription 6 is frequently activated in Hodgkin and reed-sternberg cells of Hodgkin lymphoma. Blood (2002) 99:618–26. doi: 10.1182/blood.V99.2.618 11781246

[201] Finke J Fritzen R Ternes P Trivedi P Bross KJ Lange W. Expression of bcl-2 in burkitt’s lymphoma cell lines: induction by latent Epstein-Barr virus genes. Blood (1992) 80:459–69. doi: 10.1182/blood.V80.2.459.459 1378321

[202] Wang S Rowe M Lundgren E. Expression of the Epstein Barr virus transforming protein LMP1 causes a rapid and transient stimulation of the bcl-2 homologue mcl-1 levels in b-cell lines. Cancer Res (1996) 56:4610–3. 8840972

[203] Fukuda M Longnecker R. Epstein-Barr Virus latent membrane protein 2A mediates transformation through constitutive activation of the Ras/PI3-K/Akt pathway. J Virol (2007) 81:9299–306. doi: 10.1128/JVI.00537-07 PMC1951437 17582000

[204] Moody CA Scott RS Amirghahari N Nathan CO Young LS Dawson CW. Modulation of the cell growth regulator mTOR by Epstein-Barr virus-encoded LMP2A. J Virol (2005) 79:5499–506. doi: 10.1128/JVI.79.9.5499-5506.2005 PMC1082717 15827164

[205] Chang ET Adami HO. The enigmatic epidemiology of nasopharyngeal carcinoma. Cancer Epidemiol Biomarkers Prev (2006) 15:1765–77. doi: 10.1158/1055-9965.EPI-06-0353 17035381

[206] Thompson L. World health organization classification of tumours: pathology and genetics of head and neck tumours. Ear Nose Throat J (2006) 85:74. doi: 10.1177/014556130608500201 16579185

[207] Han S Tay JK Loh CJL Chu AJM Yeong JPS Lim CM. Epstein-Barr Virus epithelial cancers-a comprehensive understanding to drive novel therapies. Front Immunol (2021) 12:734293. doi: 10.3389/fimmu.2021.734293 34956172 PMC8702733

[208] Bei JX Li Y Jia WH Feng BJ Zhou G Chen LZ. A genome-wide association study of nasopharyngeal carcinoma identifies three new susceptibility loci. Nat Genet (2010) 42:599–603. doi: 10.1038/ng.601 20512145

[209] Raghupathy R Hui EP Chan AT. Epstein-Barr Virus as a paradigm in nasopharyngeal cancer: from lab to clinic. Am Soc Clin Oncol Educ Book (2014) 149–53. doi: 10.14694/EdBook_AM.2014.34.149:149-53 24857071

[210] Chang YS Tyan YS Liu ST Tsai MS Pao CC. Detection of Epstein-Barr virus DNA sequences in nasopharyngeal carcinoma cells by enzymatic DNA amplification. J Clin Microbiol (1990) 28:2398–402. doi: 10.1128/jcm.28.11.2398-2402.1990 PMC268195 2174898

[211] Hildesheim A Levine PH. Etiology of nasopharyngeal carcinoma: a review. Epidemiol Rev (1993) 15:466–85. doi: 10.1093/oxfordjournals.epirev.a036130 8174667

[212] Xu L Huang TJ Hu H Wang MY Shi SM Yang Q. The developmental transcription factor IRF6 attenuates ABCG2 gene expression and distinctively reverses stemness phenotype in nasopharyngeal carcinoma. Cancer Lett (2018) 431:230–43. doi: 10.1016/j.canlet.2017.10.016 29111349

[213] Lo AK Dawson CW Lung HL Wong KL Young LS. The role of EBV-encoded LMP1 in the NPC tumor microenvironment: From function to therapy. Front Oncol (2021) 11:640207. doi: 10.3389/fonc.2021.640207 33718235 PMC7947715

[214] Lin DC Meng X Hazawa M Nagata Y Varela AM Xu L. The genomic landscape of nasopharyngeal carcinoma. Nat Genet (2014) 46:866–71. doi: 10.1038/ng.3006 24952746

[215] Li YY Chung GT Lui VW To KF Ma BB Chow C. Exome and genome sequencing of nasopharynx cancer identifies NF-kappaB pathway activating mutations. Nat Commun (2017) 8:14121. doi: 10.1038/ncomms14121 28098136 PMC5253631

[216] Bruce JP To KF Lui VWY Chung GTY Chan YY Tsang CM. Whole-genome profiling of nasopharyngeal carcinoma reveals viral-host co-operation in inflammatory NF-kappaB activation and immune escape. Nat Commun (2021) 12:4193. doi: 10.1038/s41467-021-24348-6 34234122 PMC8263564

[217] Cancer Genome Atlas Research N. Comprehensive molecular characterization of gastric adenocarcinoma. Nature (2014) 513:202–9. doi: 10.1038/nature13480 PMC4170219 25079317

[218] Sun K Jia K Lv H Wang SQ Wu Y Lei H. EBV-positive gastric cancer: Current knowledge and future perspectives. Front Oncol (2020) 10:583463. doi: 10.3389/fonc.2020.583463 33381453 PMC7769310

[219] Chen XZ Chen H Castro FA Hu JK Brenner H. Epstein-Barr Virus infection and gastric cancer: a systematic review. Med (Baltimore) (2015) 94:e792. doi: 10.1097/MD.0000000000000792 PMC4602887 25997049

[220] Ribeiro J Oliveira C Malta M Sousa H. Epstein-Barr Virus gene expression and latency pattern in gastric carcinomas: a systematic review. Future Oncol (2017) 13:567–79. doi: 10.2217/fon-2016-0475 28118740

[221] Chen JN Ding YG Feng ZY Li HG He D Du H. Association of distinctive Epstein-Barr virus variants with gastric carcinoma in guangzhou, southern China. J Med Virol (2010) 82:658–67. doi: 10.1002/jmv.21731 20166192

[222] van Beek J Hausen A Klein Kranenbarg E van de Velde CJ Middeldorp JM van den Brule AJ. EBV-positive gastric adenocarcinomas: a distinct clinicopathologic entity with a low frequency of lymph node involvement. J Clin Oncol (2004) 22:664–70. doi: 10.1200/JCO.2004.08.061 14966089

[223] Abe H Kaneda A Fukayama M. Epstein-Barr Virus-associated gastric carcinoma: Use of host cell machineries and somatic gene mutations. Pathobiology (2015) 82:212–23. doi: 10.1159/000434683 26337667

[224] Liang Q Yao X Tang S Zhang J Yau TO Li X. Integrative identification of Epstein-Barr virus-associated mutations and epigenetic alterations in gastric cancer. Gastroenterology (2014) 147:1350–62 e4. doi: 10.1053/j.gastro.2014.08.036 25173755

[225] Leung SY Chau KY Yuen ST Chu KM Branicki FJ Chung LP. p53 overexpression is different in Epstein-Barr virus-associated and Epstein-Barr virus-negative carcinoma. Histopathology (1998) 33:311–7. 9822919

[226] Tchelebi L Ashamalla H Graves PR. Mutant p53 and the response to chemotherapy and radiation. Subcell Biochem (2014) 85:133–59. doi: 10.1007/978-94-017-9211-0_8 25201193

[227] Kaneda A Matsusaka K Aburatani H Fukayama M. Epstein-Barr Virus infection as an epigenetic driver of tumorigenesis. Cancer Res (2012) 72:3445–50. doi: 10.1158/0008-5472.CAN-11-3919 22761333

[228] Panda A Mehnert JM Hirshfield KM Riedlinger G Damare S Saunders T. Immune activation and benefit from avelumab in EBV-positive gastric cancer. J Natl Cancer Inst (2018) 110:316–20. doi: 10.1093/jnci/djx213 PMC6658862 29155997

[229] Ressing ME van Gent M Gram AM Hooykaas MJ Piersma SJ Wiertz EJ. Immune evasion by Epstein-Barr virus. Curr Top Microbiol Immunol (2015) 391:355–81. doi: 10.1007/978-3-319-22834-1_12 26428381

[230] Gallo A Miele M Badami E Conaldi PG. Molecular and cellular interplay in virus-induced tumors in solid organ recipients. Cell Immunol (2019) 343:103770. doi: 10.1016/j.cellimm.2018.02.010 29523417

[231] Bauer M Jasinski-Bergner S Mandelboim O Wickenhauser C Seliger B. Epstein-Barr Virus-associated malignancies and immune escape: The role of the tumor microenvironment and tumor cell evasion strategies. Cancers (Basel) (2021) 13(20):5189. doi: 10.3390/cancers13205189 34680337 PMC8533749

[232] van Beek J zur Hausen A Snel SN Berkhof J Kranenbarg EK van de Velde CJ. Morphological evidence of an activated cytotoxic T-cell infiltrate in EBV-positive gastric carcinoma preventing lymph node metastases. Am J Surg Pathol (2006) 30:59–65. doi: 10.1097/01.pas.0000176428.06629.1e 16330943

[233] Strong MJ Xu G Coco J Baribault C Vinay DS Lacey MR. Differences in gastric carcinoma microenvironment stratify according to EBV infection intensity: implications for possible immune adjuvant therapy. PloS Pathog (2013) 9:e1003341. doi: 10.1371/journal.ppat.1003341 23671415 PMC3649992

[234] Liu Y He S Wang XL Peng W Chen QY Chi DM. Tumour heterogeneity and intercellular networks of nasopharyngeal carcinoma at single cell resolution. Nat Commun (2021) 12:741. doi: 10.1038/s41467-021-21043-4 33531485 PMC7854640

[235] Zhao J Guo C Xiong F Yu J Ge J Wang H. Single cell RNA-seq reveals the landscape of tumor and infiltrating immune cells in nasopharyngeal carcinoma. Cancer Lett (2020) 477:131–43. doi: 10.1016/j.canlet.2020.02.010 32061950

[236] Cai TT Ye SB Liu YN He J Chen QY Mai HQ. LMP1-mediated glycolysis induces myeloid-derived suppressor cell expansion in nasopharyngeal carcinoma. PloS Pathog (2017) 13:e1006503. doi: 10.1371/journal.ppat.1006503 28732079 PMC5540616

[237] Roemer MG Advani RH Ligon AH Natkunam Y Redd RA Homer H. PD-L1 and PD-L2 genetic alterations define classical Hodgkin lymphoma and predict outcome. J Clin Oncol (2016) 34:2690–7. doi: 10.1200/JCO.2016.66.4482 PMC5019753 27069084

[238] Granai M Mundo L Akarca AU Siciliano MC Rizvi H Mancini V. Immune landscape in burkitt lymphoma reveals M2-macrophage polarization and correlation between PD-L1 expression and non-canonical EBV latency program. Infect Agent Cancer (2020) 15:28. doi: 10.1186/s13027-020-00292-w 32391073 PMC7201729

[239] Baumforth KR Birgersdotter A Reynolds GM Wei W Kapatai G Flavell JR. Expression of the Epstein-Barr virus-encoded Epstein-Barr virus nuclear antigen 1 in hodgkin’s lymphoma cells mediates up-regulation of CCL20 and the migration of regulatory T cells. Am J Pathol (2008) 173:195–204. doi: 10.2353/ajpath.2008.070845 18502823 PMC2438297

[240] Mrizak D Martin N Barjon C Jimenez-Pailhes AS Mustapha R Niki T. Effect of nasopharyngeal carcinoma-derived exosomes on human regulatory T cells. J Natl Cancer Inst (2015) 107:363. doi: 10.1093/jnci/dju363 25505237

[241] de Waal Malefyt R Haanen J Spits H Roncarolo MG te Velde A Figdor C. Interleukin 10 (IL-10) and viral IL-10 strongly reduce antigen-specific human T cell proliferation by diminishing the antigen-presenting capacity of monocytes *via* downregulation of class II major histocompatibility complex expression. J Exp Med (1991) 174:915–24. doi: 10.1084/jem.174.4.915 PMC2118975 1655948

[242] Marshall NA Vickers MA Barker RN. Regulatory T cells secreting IL-10 dominate the immune response to EBV latent membrane protein 1. J Immunol (2003) 170:6183–9. doi: 10.4049/jimmunol.170.12.6183 12794149

[243] Ren Y Yang J Li M Huang N Chen Y Wu X. Viral IL-10 promotes cell proliferation and cell cycle progression *via* JAK2/STAT3 signaling pathway in nasopharyngeal carcinoma cells. Biotechnol Appl Biochem (2020) 67:929–38. doi: 10.1002/bab.1856 31737947

[244] Zhang NN Chen JN Xiao L Tang F Zhang ZG Zhang YW. Accumulation mechanisms of CD4(+)CD25(+)FOXP3(+) regulatory T cells in EBV-associated gastric carcinoma. Sci Rep (2015) 5:18057. doi: 10.1038/srep18057 26673957 PMC4682180

[245] Ohtani H Nakayama T Yoshie O. *In situ* expression of the CCL20-CCR6 axis in lymphocyte-rich gastric cancer and its potential role in the formation of lymphoid stroma. Pathol Int (2011) 61:645–51. doi: 10.1111/j.1440-1827.2011.02717.x 22029675

[246] Chen J Yang P Xiao Y Zhang Y Liu J Xie D. Overexpression of alpha-sma-positive fibroblasts (CAFs) in nasopharyngeal carcinoma predicts poor prognosis. J Cancer (2017) 8:3897–902. doi: 10.7150/jca.20324 PMC5688944 29151978

[247] Biggi AFB Elgui de Oliveira D. The Epstein-Barr virus hacks immune checkpoints: Evidence and consequences for lymphoproliferative disorders and cancers. Biomolecules (2022) 12(3):397. doi: 10.3390/biom12030397 35327589 PMC8946074

[248] Munz C. Natural killer cell responses during human gamma-herpesvirus infections. Vaccines (Basel) (2021) 9. doi: 10.3390/vaccines9060655 PMC8232711 34203904

[249] Pappworth IY Wang EC Rowe M. The switch from latent to productive infection in epstein-barr virus-infected b cells is associated with sensitization to NK cell killing. J Virol (2007) 81:474–82. doi: 10.1128/JVI.01777-06 PMC1797427 17079298

[250] Chijioke O Muller A Feederle R Barros MH Krieg C Emmel V. Human natural killer cells prevent infectious mononucleosis features by targeting lytic Epstein-Barr virus infection. Cell Rep (2013) 5:1489–98. doi: 10.1016/j.celrep.2013.11.041 PMC3895765 24360958

[251] Jangra S Yuen KS Botelho MG Jin DY. Epstein-Barr Virus and innate immunity: Friends or foes? Microorganisms (2019) 7(6):183. doi: 10.3390/microorganisms7060183 PMC6617214 31238570

[252] Middeldorp JM. Epstein-Barr Virus-specific humoral immune responses in health and disease. Curr Top Microbiol Immunol (2015) 391:289–323. doi: 10.1007/978-3-319-22834-1_10 26428379

[253] Cruz-Tapias P CJ Anaya JM. Major histocompatibility complex: Antigen processing and presentation. autoimmunity: From bench to bedside bogota (Colombia). El Rosario University Press (2013). 29087650

[254] Bushkin Y Demaria S Mohagheghpour N Le JM. Activation of human CD8-positive T cells *via* the CD8/HLA class I complex. Cell Immunol (1990) 126:185–95. doi: 10.1016/0008-8749(90)90311-E 2105853

[255] Kuzushima K Nakamura S Nakamura T Yamamura Y Yokoyama N Fujita M. Increased frequency of antigen-specific CD8(+) cytotoxic T lymphocytes infiltrating an Epstein-Barr virus-associated gastric carcinoma. J Clin Invest (1999) 104:163–71. doi: 10.1172/JCI6062 PMC408473 10411545

[256] Miliotis CN Slack FJ. Multi-layered control of PD-L1 expression in Epstein-Barr virus-associated gastric cancer. J Cancer Metastasis Treat (2020) 6(13). doi: 10.20517/2394-4722.2020.12 PMC8244904 34212113

[257] Sharpe AH Pauken KE. The diverse functions of the PD1 inhibitory pathway. Nat Rev Immunol (2018) 18:153–67. doi: 10.1038/nri.2017.108 28990585

[258] Patel SP Kurzrock R. PD-L1 expression as a predictive biomarker in cancer immunotherapy. Mol Cancer Ther (2015) 14:847–56. doi: 10.1158/1535-7163.MCT-14-0983 25695955

[259] Ishida Y Agata Y Shibahara K Honjo T. Induced expression of PD-1, a novel member of the immunoglobulin gene superfamily, upon programmed cell death. EMBO J (1992) 11:3887–95. doi: 10.1002/j.1460-2075.1992.tb05481.x PMC556898 1396582

[260] Sharpe AH Wherry EJ Ahmed R Freeman GJ. The function of programmed cell death 1 and its ligands in regulating autoimmunity and infection. Nat Immunol (2007) 8:239–45. doi: 10.1038/ni1443 17304234

[261] Keir ME Butte MJ Freeman GJ Sharpe AH. PD-1 and its ligands in tolerance and immunity. Annu Rev Immunol (2008) 26:677–704. doi: 10.1146/annurev.immunol.26.021607.090331 18173375 PMC10637733

[262] Okazaki T Chikuma S Iwai Y Fagarasan S Honjo T. A rheostat for immune responses: the unique properties of PD-1 and their advantages for clinical application. Nat Immunol (2013) 14:1212–8. doi: 10.1038/ni.2762 24240160

[263] Honda T Egen JG Lämmermann T Kastenmüller W Torabi-Parizi P Germain RN. Tuning of antigen sensitivity by T cell receptor-dependent negative feedback controls T cell effector function in inflamed tissues. Immunity (2014) 40:235–47. doi: 10.1016/j.immuni.2013.11.017 PMC4792276 24440150

[264] Nayak L Iwamoto FM LaCasce A Mukundan S Roemer MGM Chapuy B. PD-1 blockade with nivolumab in relapsed/refractory primary central nervous system and testicular lymphoma. Blood (2017) 129:3071–3. doi: 10.1182/blood-2017-01-764209 PMC5766844 28356247

[265] Dong H Strome SE Salomao DR Tamura H Hirano F Flies DB. Tumor-associated B7-H1 promotes T-cell apoptosis: a potential mechanism of immune evasion. Nat Med (2002) 8:793–800. doi: 10.1038/nm730 12091876

[266] Schonrich G Raftery MJ. The PD-1/PD-L1 axis and virus infections: A delicate balance. Front Cell Infect Microbiol (2019) 9:207. doi: 10.3389/fcimb.2019.00207 31263684 PMC6584848

[267] Kataoka K Miyoshi H Sakata S Dobashi A Couronne L Kogure Y. Frequent structural variations involving programmed death ligands in Epstein-Barr virus-associated lymphomas. Leukemia (2019) 33:1687–99. doi: 10.1038/s41375-019-0380-5 PMC6755969 30683910

[268] Hou Y Wu K Shi X Li F Song L Wu H. Comparison of variations detection between whole-genome amplification methods used in single-cell resequencing. Gigascience (2015) 4:37. doi: 10.1186/s13742-015-0068-3 26251698 PMC4527218

[269] Morales-Sanchez A Fuentes-Panana EM. Epstein-Barr Virus-associated gastric cancer and potential mechanisms of oncogenesis. Curr Cancer Drug Targets (2017) 17:534–54. doi: 10.2174/1568009616666160926124923 27677953

[270] Xu-Monette ZY Zhou J Young KH. PD-1 expression and clinical PD-1 blockade in b-cell lymphomas. Blood (2018) 131:68–83. doi: 10.1182/blood-2017-07-740993 29118007 PMC5755041

[271] Green MR Monti S Rodig SJ Juszczynski P Currie T O’Donnell E. Integrative analysis reveals selective 9p24.1 amplification, increased PD-1 ligand expression, and further induction *via* JAK2 in nodular sclerosing Hodgkin lymphoma and primary mediastinal large b-cell lymphoma. Blood (2010) 116:3268–77. doi: 10.1182/blood-2010-05-282780 PMC2995356 20628145

[272] Chen BJ Chapuy B Ouyang J Sun HH Roemer MG Xu ML. PD-L1 expression is characteristic of a subset of aggressive b-cell lymphomas and virus-associated malignancies. Clin Cancer Res (2013) 19:3462–73. doi: 10.1158/1078-0432.CCR-13-0855 PMC4102335 23674495

[273] Cerezo M Guemiri R Druillennec S Girault I Malka-Mahieu H Shen S. Translational control of tumor immune escape *via* the eIF4F-STAT1-PD-L1 axis in melanoma. Nat Med (2018) 24:1877–86. doi: 10.1038/s41591-018-0217-1 30374200

[274] Jiang X Zhou J Giobbie-Hurder A Wargo J Hodi FS. The activation of MAPK in melanoma cells resistant to BRAF inhibition promotes PD-L1 expression that is reversible by MEK and PI3K inhibition. Clin Cancer Res (2013) 19:598–609. doi: 10.1158/1078-0432.CCR-12-2731 23095323

[275] Coelho MA de Carné Trécesson S Rana S Zecchin D Moore C Molina-Arcas M. Oncogenic RAS signaling promotes tumor immunoresistance by stabilizing PD-L1 mRNA. Immunity (2017) 47:1083–1099.e6. doi: 10.1016/j.immuni.2017.11.016 29246442 PMC5746170

[276] Kataoka K Shiraishi Y Takeda Y Sakata S Matsumoto M Nagano S. Aberrant PD-L1 expression through 3’-UTR disruption in multiple cancers. Nature (2016) 534:402–6. doi: 10.1038/nature18294 27281199

[277] Cayrol C Flemington EK. Identification of cellular target genes of the Epstein-Barr virus transactivator zta: activation of transforming growth factor beta igh3 (TGF-beta igh3) and TGF-beta 1. J Virol (1995) 69:4206–12. doi: 10.1128/jvi.69.7.4206-4212.1995 PMC189158 7769680

[278] Ramasubramanyan S Osborn K Al-Mohammad R Naranjo Perez-Fernandez IB Zuo J Balan N. Epstein-Barr Virus transcription factor zta acts through distal regulatory elements to directly control cellular gene expression. Nucleic Acids Res (2015) 43:3563–77. doi: 10.1093/nar/gkv212 PMC4402532 25779048

[279] Ndour PA Brocqueville G Ouk TS Goormachtigh G Morales O Mougel A. Inhibition of latent membrane protein 1 impairs the growth and tumorigenesis of latency II Epstein-Barr virus-transformed T cells. J Virol (2012) 86:3934–43. doi: 10.1128/JVI.05747-11 PMC3302486 22258264

[280] Green MR Rodig S Juszczynski P Ouyang J Sinha P O’Donnell E. Constitutive AP-1 activity and EBV infection induce PD-L1 in Hodgkin lymphomas and posttransplant lymphoproliferative disorders: implications for targeted therapy. Clin Cancer Res (2012) 18:1611–8. doi: 10.1158/1078-0432.CCR-11-1942 PMC3321508 22271878

[281] Fang W Zhang J Hong S Zhan J Chen N Qin T. EBV-driven LMP1 and IFN-gamma up-regulate PD-L1 in nasopharyngeal carcinoma: Implications for oncotargeted therapy. Oncotarget (2014) 5:12189–202. doi: 10.18632/oncotarget.2608 PMC4322961 25361008

[282] Bi XW Wang H Zhang WW Wang JH Liu WJ Xia ZJ. PD-L1 is upregulated by EBV-driven LMP1 through NF-κB pathway and correlates with poor prognosis in natural killer/T-cell lymphoma. J Hematol Oncol (2016) 9:109. doi: 10.1186/s13045-016-0341-7 27737703 PMC5064887

[283] Vereide DT Sugden B. Lymphomas differ in their dependence on Epstein-Barr virus. Blood (2011) 117:1977–85. doi: 10.1182/blood-2010-05-285791 PMC3056644 21088132

[284] Moon JW Kong SK Kim BS Kim HJ Lim H Noh K. IFNgamma induces PD-L1 overexpression by JAK2/STAT1/IRF-1 signaling in EBV-positive gastric carcinoma. Sci Rep (2017) 7:17810. doi: 10.1038/s41598-017-18132-0 29259270 PMC5736657

[285] Dasari V Sinha D Neller MA Smith C Khanna R. Prophylactic and therapeutic strategies for Epstein-Barr virus-associated diseases: emerging strategies for clinical development. Expert Rev Vaccines (2019) 18:457–74. doi: 10.1080/14760584.2019.1605906 30987475

[286] Chodosh J Holder VP Gan YJ Belgaumi A Sample J Sixbey JW. Eradication of latent Epstein-Barr virus by hydroxyurea alters the growth-transformed cell phenotype. J Infect Dis (1998) 177:1194–201. doi: 10.1086/515290 9593003

[287] Armand P Engert A Younes A Fanale M Santoro A Zinzani PL. Nivolumab for Relapsed/Refractory classic Hodgkin lymphoma after failure of autologous hematopoietic cell transplantation: Extended follow-up of the multicohort single-arm phase II CheckMate 205 trial. J Clin Oncol (2018) 36:1428–39. doi: 10.1200/JCO.2017.76.0793 PMC6075855 29584546

[288] Janjigian YY Shitara K Moehler M Garrido M Salman P Shen L. First-line nivolumab plus chemotherapy versus chemotherapy alone for advanced gastric, gastro-oesophageal junction, and oesophageal adenocarcinoma (CheckMate 649): a randomised, open-label, phase 3 trial. Lancet (2021) 398:27–40. doi: 10.1016/S0140-6736(21)00797-2 34102137 PMC8436782

[289] Kang YK Boku N Satoh T Ryu MH Chao Y Kato K. Nivolumab in patients with advanced gastric or gastro-oesophageal junction cancer refractory to, or intolerant of, at least two previous chemotherapy regimens (ONO-4538-12, ATTRACTION-2): a randomised, double-blind, placebo-controlled, phase 3 trial. Lancet (2017) 390:2461–71. doi: 10.1016/S0140-6736(17)31827-5 28993052

[290] Ferris RL Blumenschein G Jr. Fayette J Guigay J Colevas AD Licitra L. Nivolumab for recurrent squamous-cell carcinoma of the head and neck. N Engl J Med (2016) 375:1856–67. doi: 10.1056/NEJMoa1602252 PMC5564292 27718784

[291] Fuchs CS Doi T Jang RW Muro K Satoh T Machado M. Safety and efficacy of pembrolizumab monotherapy in patients with previously treated advanced gastric and gastroesophageal junction cancer: Phase 2 clinical KEYNOTE-059 trial. JAMA Oncol (2018) 4:e180013. doi: 10.1001/jamaoncol.2018.0013 29543932 PMC5885175

[292] Hsu C Lee SH Ejadi S Even C Cohen RB Le Tourneau C. Safety and antitumor activity of pembrolizumab in patients with programmed death-ligand 1-positive nasopharyngeal carcinoma: Results of the KEYNOTE-028 study. J Clin Oncol (2017) 35:4050–6. doi: 10.1200/JCO.2017.73.3675 28837405

[293] Burtness B Harrington KJ Greil R Soulieres D Tahara M de Castro G Jr.. Pembrolizumab alone or with chemotherapy versus cetuximab with chemotherapy for recurrent or metastatic squamous cell carcinoma of the head and neck (KEYNOTE-048): a randomised, open-label, phase 3 study. Lancet (2019) 394:1915–28. doi: 10.1016/S0140-6736(19)32591-7 31679945

[294] Lv K Yin T Yu M Chen Z Zhou Y Li F. Treatment advances in EBV related lymphoproliferative diseases. Front Oncol (2022) 12:838817. doi: 10.3389/fonc.2022.838817 35515118 PMC9063483

[295] Corallo S Fuca G Morano F Salati M Spallanzani A Gloghini A. Clinical behavior and treatment response of Epstein-Barr virus-positive metastatic gastric cancer: Implications for the development of future trials. Oncologist (2020) 25:780–6. doi: 10.1634/theoncologist.2020-0037 PMC7485344 32272500

[296] Qiu MZ He CY Yang DJ Zhou DL Zhao BW Wang XJ. Observational cohort study of clinical outcome in Epstein-Barr virus associated gastric cancer patients. Ther Adv Med Oncol (2020) 12:1758835920937434. doi: 10.1177/1758835920937434 32670421 PMC7338646

[297] Xie T Peng Z Liu Y Zhang Z Zhang X Li J. Clinicopathological characteristics and response to chemotherapy in treatment-naive Epstein-Barr virus associated gastric cancer: A retrospective study. Front Oncol (2021) 11:611676. doi: 10.3389/fonc.2021.611676 34631508 PMC8495155

[298] Papadopoulos EB Ladanyi M Emanuel D Mackinnon S Boulad F Carabasi MH. Infusions of donor leukocytes to treat Epstein-Barr virus-associated lymphoproliferative disorders after allogeneic bone marrow transplantation. N Engl J Med (1994) 330:1185–91. doi: 10.1056/NEJM199404283301703 8093146

[299] Cui X Snapper CM. Epstein Barr Virus: Development of vaccines and immune cell therapy for EBV-associated diseases. Front Immunol (2021) 12:734471. doi: 10.3389/fimmu.2021.734471 34691042 PMC8532523

[300] Rooney CM Smith CA Ng CY Loftin S Li C Krance RA. Use of gene-modified virus-specific T lymphocytes to control Epstein-barr-virus-related lymphoproliferation. Lancet (1995) 345:9–13. doi: 10.1016/S0140-6736(95)91150-2 7799740

[301] Rooney CM Smith CA Ng CY Loftin SK Sixbey JW Gan Y. Infusion of cytotoxic T cells for the prevention and treatment of Epstein-Barr virus-induced lymphoma in allogeneic transplant recipients. Blood (1998) 92:1549–55. doi: 10.1182/blood.V92.5.1549.417k32_1549_1555 9716582

[302] Heslop HE Ng CY Li C Smith CA Loftin SK Krance RA. Long-term restoration of immunity against Epstein-Barr virus infection by adoptive transfer of gene-modified virus-specific T lymphocytes. Nat Med (1996) 2:551–5. doi: 10.1038/nm0596-551 8616714

[303] Heslop HE Slobod KS Pule MA Hale GA Rousseau A Smith CA. Long-term outcome of EBV-specific T-cell infusions to prevent or treat EBV-related lymphoproliferative disease in transplant recipients. Blood (2010) 115:925–35. doi: 10.1182/blood-2009-08-239186 PMC2817637 19880495

[304] Comoli P Pedrazzoli P Maccario R Basso S Carminati O Labirio M. Cell therapy of stage IV nasopharyngeal carcinoma with autologous Epstein-Barr virus-targeted cytotoxic T lymphocytes. J Clin Oncol (2005) 23:8942–9. doi: 10.1200/JCO.2005.02.6195 16204009

[305] Louis CU Straathof K Bollard CM Ennamuri S Gerken C Lopez TT. Adoptive transfer of EBV-specific T cells results in sustained clinical responses in patients with locoregional nasopharyngeal carcinoma. J Immunother (2010) 33:983–90. doi: 10.1097/CJI.0b013e3181f3cbf4 PMC2964409 20948438

[306] Huang J Fogg M Wirth LJ Daley H Ritz J Posner MR. Epstein-Barr Virus-specific adoptive immunotherapy for recurrent, metastatic nasopharyngeal carcinoma. Cancer (2017) 123:2642–50. doi: 10.1002/cncr.30541 28222215

[307] Chia WK Teo M Wang WW Lee B Ang SF Tai WM. Adoptive T-cell transfer and chemotherapy in the first-line treatment of metastatic and/or locally recurrent nasopharyngeal carcinoma. Mol Ther (2014) 22:132–9. doi: 10.1038/mt.2013.242 PMC3978790 24297049

[308] Bollard CM Gottschalk S Torrano V Diouf O Ku S Hazrat Y. Sustained complete responses in patients with lymphoma receiving autologous cytotoxic T lymphocytes targeting Epstein-Barr virus latent membrane proteins. J Clin Oncol (2014) 32:798–808. doi: 10.1200/JCO.2013.51.5304 24344220 PMC3940538

[309] Zheng Y Parsonage G Zhuang X Machado LR James CH Salman A. Human leukocyte antigen (HLA) A*1101-restricted Epstein-Barr virus-specific T-cell receptor gene transfer to target nasopharyngeal carcinoma. Cancer Immunol Res (2015) 3:1138–47. doi: 10.1158/2326-6066.CIR-14-0203-T PMC4456157 25711537

[310] Cho HI Kim UH Shin AR Won JN Lee HJ Sohn HJ. A novel Epstein-Barr virus-latent membrane protein-1-specific T-cell receptor for TCR gene therapy. Br J Cancer (2018) 118:534–45. doi: 10.1038/bjc.2017.475 PMC5830600 29360818

[311] Fousek K Watanabe J Joseph SK George A An X Byrd TT. CAR T-cells that target acute b-lineage leukemia irrespective of CD19 expression. Leukemia (2021) 35:75–89. doi: 10.1038/s41375-020-0792-2 32205861 PMC7519582

[312] Liu E Marin D Banerjee P Macapinlac HA Thompson P Basar R. Use of CAR-transduced natural killer cells in CD19-positive lymphoid tumors. N Engl J Med (2020) 382:545–53. doi: 10.1056/NEJMoa1910607 PMC7101242 32023374

[313] Wang M Munoz J Goy A Locke FL Jacobson CA Hill BT. KTE-X19 CAR T-cell therapy in relapsed or refractory mantle-cell lymphoma. N Engl J Med (2020) 382:1331–42. doi: 10.1056/NEJMoa1914347 PMC7731441 32242358

[314] Chen R Zhang D Mao Y Zhu J Ming H Wen J. A human fab-based immunoconjugate specific for the LMP1 extracellular domain inhibits nasopharyngeal carcinoma growth *in vitro* and *in vivo* . Mol Cancer Ther (2012) 11:594–603. doi: 10.1158/1535-7163.MCT-11-0725 22169768

[315] Tang X Tang Q Mao Y Huang X Jia L Zhu J. CD137 Co-stimulation improves the antitumor effect of LMP1-specific chimeric antigen receptor T cells *In vitro* and *In vivo* . Onco Targets Ther (2019) 12:9341–50. doi: 10.2147/OTT.S221040 PMC6847990 31807014

[316] Poole CL James SH. Antiviral therapies for herpesviruses: Current agents and new directions. Clin Ther (2018) 40:1282–98. doi: 10.1016/j.clinthera.2018.07.006 PMC7728158 30104016

[317] Pagano JS Datta AK. Perspectives on interactions of acyclovir with Epstein-Barr and other herpes viruses. Am J Med (1982) 73:18–26. doi: 10.1016/0002-9343(82)90057-2 6285710

[318] Pagano JS Sixbey JW Lin JC. Acyclovir and Epstein-Barr virus infection. J Antimicrob Chemother (1983) 12(Suppl B):113–21. doi: 10.1093/jac/12.suppl_B.113 6313591

[319] Malouf MA Chhajed PN Hopkins P Plit M Turner J Glanville AR. Anti-viral prophylaxis reduces the incidence of lymphoproliferative disease in lung transplant recipients. J Heart Lung Transplant (2002) 21:547–54. doi: 10.1016/S1053-2498(01)00407-7 11983544

[320] Hocker B Bohm S Fickenscher H Kusters U Schnitzler P Pohl M. (Val-)Ganciclovir prophylaxis reduces Epstein-Barr virus primary infection in pediatric renal transplantation. Transpl Int (2012) 25:723–31. doi: 10.1111/j.1432-2277.2012.01485.x 22533698

[321] Tynell E Aurelius E Brandell A Julander I Wood M Yao QY. Acyclovir and prednisolone treatment of acute infectious mononucleosis: a multicenter, double-blind, placebo-controlled study. J Infect Dis (1996) 174:324–31. doi: 10.1093/infdis/174.2.324 8699062

[322] Colby BM Shaw JE Elion GB Pagano JS. Effect of acyclovir [9-(2-hydroxyethoxymethyl)guanine] on Epstein-Barr virus DNA replication. J Virol (1980) 34:560–8. doi: 10.1128/jvi.34.2.560-568.1980 PMC288736 6246281

[323] Abele G Eriksson B Harmenberg J Wahren B. Inhibition of varicella-zoster virus-induced DNA polymerase by a new guanosine analog, 9-[4-hydroxy-2-(hydroxymethyl)butyl]guanine triphosphate. Antimicrob Agents Chemother (1988) 32:1137–42. doi: 10.1128/AAC.32.8.1137 PMC172365 2847643

[324] Abdulkarim B Bourhis J. Antiviral approaches for cancers related to Epstein-Barr virus and human papillomavirus. Lancet Oncol (2001) 2:622–30. doi: 10.1016/S1470-2045(01)00520-4 11902553

[325] Neyts J Sadler R De Clercq E Raab-Traub N Pagano JS. The antiviral agent cidofovir [(S)-1-(3-hydroxy-2-phosphonyl-methoxypropyl)cytosine] has pronounced activity against nasopharyngeal carcinoma grown in nude mice. Cancer Res (1998) 58:384–8. 9458076

[326] Yoshizaki T Wakisaka N Kondo S Murono S Shimizu Y Nakashima M. Treatment of locally recurrent Epstein-Barr virus-associated nasopharyngeal carcinoma using the anti-viral agent cidofovir. J Med Virol (2008) 80:879–82. doi: 10.1002/jmv.21165 18360900

[327] Abdulkarim B Sabri S Zelenika D Deutsch E Frascogna V Klijanienko J. Antiviral agent cidofovir decreases Epstein-Barr virus (EBV) oncoproteins and enhances the radiosensitivity in EBV-related malignancies. Oncogene (2003) 22:2260–71. doi: 10.1038/sj.onc.1206402 12700662

[328] De Clercq E Holy A. Acyclic nucleoside phosphonates: a key class of antiviral drugs. Nat Rev Drug Discovery (2005) 4:928–40. doi: 10.1038/nrd1877 16264436

[329] Gallant JE Daar ES Raffi F Brinson C Ruane P DeJesus E. Efficacy and safety of tenofovir alafenamide versus tenofovir disoproxil fumarate given as fixed-dose combinations containing emtricitabine as backbones for treatment of HIV-1 infection in virologically suppressed adults: a randomised, double-blind, active-controlled phase 3 trial. Lancet HIV (2016) 3:e158–65. doi: 10.1016/S2352-3018(16)00024-2 27036991

[330] De Clercq E. Tenofovir alafenamide (TAF) as the successor of tenofovir disoproxil fumarate (TDF). Biochem Pharmacol (2016) 119:1–7. doi: 10.1016/j.bcp.2016.04.015 27133890

[331] Drosu NC Edelman ER Housman DE. Tenofovir prodrugs potently inhibit Epstein-Barr virus lytic DNA replication by targeting the viral DNA polymerase. Proc Natl Acad Sci U.S.A. (2020) 117:12368–74. doi: 10.1073/pnas.2002392117 PMC7275665 32409608

[332] Wagstaff AJ Bryson HM. Foscarnet. a reappraisal of its antiviral activity, pharmacokinetic properties and therapeutic use in immunocompromised patients with viral infections. Drugs (1994) 48:199–226. doi: 10.2165/00003495-199448020-00007 7527325

[333] Oertel SH Ruhnke MS Anagnostopoulos I Kahl AA Frewer AF Bechstein WO. Treatment of Epstein-Barr virus-induced posttransplantation lymphoproliferative disorder with foscarnet alone in an adult after simultaneous heart and renal transplantation. Transplantation (1999) 67:765–7. doi: 10.1097/00007890-199903150-00023 10096538

[334] Afshar K Rao AP Patel V Forrester K Ganesh S. Use of foscarnet therapy for EBV infection following control of PTLD with enhancement of cellular immunity in a lung-transplant recipient. J Transplant (2011) 2011:919651. doi: 10.1155/2011/919651 21423547 PMC3056220

[335] Israel BF Kenney SC. Virally targeted therapies for EBV-associated malignancies. Oncogene (2003) 22:5122–30. doi: 10.1038/sj.onc.1206548 12910249

[336] Oker N Kapetanakis N-I Busson P. Review: Biological and pharmacological basis of cytolytic viral activation in EBV-associated nasopharyngeal carcinoma, herpesviridae. (2016). doi: 10.5772/64738

[337] Middeldorp JM Novalić Z Verkuijlen SAWM Greijer AE Middeldorp JM. Cytolytic Epstein-Barr virus reactivation therapy for EBV-associated gastric carcinoma. Clin Oncol Res (2020). doi: 10.31487/j.COR.2020.07.08

[338] Wildeman MA Novalic Z Verkuijlen SA Juwana H Huitema AD Tan IB. Cytolytic virus activation therapy for Epstein-Barr virus-driven tumors. Clin Cancer Res (2012) 18:5061–70. doi: 10.1158/1078-0432.CCR-12-0574 22761471

[339] Yiu SPT Dorothea M Hui KF Chiang AKS. Lytic induction therapy against Epstein-Barr virus-associated malignancies: Past, present, and future. Cancers (Basel) (2020) 12(8):2142. doi: 10.3390/cancers12082142 PMC7465660 32748879

[340] Kim SJ Kim JH Ki CS Ko YH Kim JS Kim WS. Epstein-Barr Virus reactivation in extranodal natural killer/T-cell lymphoma patients: a previously unrecognized serious adverse event in a pilot study with romidepsin. Ann Oncol (2016) 27:508–13. doi: 10.1093/annonc/mdv596 26658891

[341] Hui KF Ho DN Tsang CM Middeldorp JM Tsao GS Chiang AK. Activation of lytic cycle of Epstein-Barr virus by suberoylanilide hydroxamic acid leads to apoptosis and tumor growth suppression of nasopharyngeal carcinoma. Int J Cancer (2012) 131:1930–40. doi: 10.1002/ijc.27439 22261816

[342] Choi CK Ho DN Hui KF Kao RY Chiang AK. Identification of novel small organic compounds with diverse structures for the induction of Epstein-Barr virus (EBV) lytic cycle in EBV-positive epithelial malignancies. PloS One (2015) 10:e0145994. doi: 10.1371/journal.pone.0145994 26717578 PMC4696655

[343] Hui KF Chiang AK. Suberoylanilide hydroxamic acid induces viral lytic cycle in Epstein-Barr virus-positive epithelial malignancies and mediates enhanced cell death. Int J Cancer (2010) 126:2479–89. doi: 10.1002/ijc.24945 19816947

[344] Perrine SP Hermine O Small T Suarez F O’Reilly R Boulad F. A phase 1/2 trial of arginine butyrate and ganciclovir in patients with Epstein-Barr virus-associated lymphoid malignancies. Blood (2007) 109:2571–8. doi: 10.1182/blood-2006-01-024703 PMC1852196 17119113

[345] Ghosh SK Perrine SP Faller DV. Advances in virus-directed therapeutics against Epstein-Barr virus-associated malignancies. Adv Virol (2012) 2012:509296. doi: 10.1155/2012/509296 22500168 PMC3303631

[346] Ghosh SK Perrine SP Williams RM Faller DV. Histone deacetylase inhibitors are potent inducers of gene expression in latent EBV and sensitize lymphoma cells to nucleoside antiviral agents. Blood (2012) 119:1008–17. doi: 10.1182/blood-2011-06-362434 PMC3271713 22160379

[347] Daigle D Gradoville L Tuck D Schulz V Wang’ondu R Ye J. Valproic acid antagonizes the capacity of other histone deacetylase inhibitors to activate the Epstein-barr virus lytic cycle. J Virol (2011) 85:5628–43. doi: 10.1128/JVI.02659-10 PMC3094991 21411522

[348] Bierer BE Nathan DG. The effect of desferrithiocin, an oral iron chelator, on T-cell function. Blood (1990) 76:2052–9. doi: 10.1182/blood.V76.10.2052.2052 2242426

[349] Kraus RJ Yu X Cordes BA Sathiamoorthi S Iempridee T Nawandar DM. Hypoxia-inducible factor-1alpha plays roles in Epstein-Barr virus’s natural life cycle and tumorigenesis by inducing lytic infection through direct binding to the immediate-early BZLF1 gene promoter. PloS Pathog (2017) 13:e1006404. doi: 10.1371/journal.ppat.1006404 28617871 PMC5487075

[350] Yiu SPT Hui KF Choi CK Kao RYT Ma CW Yang D. Intracellular iron chelation by a novel compound, C7, reactivates epstein(-)Barr virus (EBV) lytic cycle *via* the ERK-autophagy axis in EBV-positive epithelial cancers. Cancers (Basel) (2018) 10(12):505. doi: 10.3390/cancers10120505 PMC6316324 30544928

[351] Lee EK Kim SY Noh KW Joo EH Zhao B Kieff E. Small molecule inhibition of Epstein-Barr virus nuclear antigen-1 DNA binding activity interferes with replication and persistence of the viral genome. Antiviral Res (2014) 104:73–83. doi: 10.1016/j.antiviral.2014.01.018 24486954 PMC3964181

[352] Gianti E Messick TE Lieberman PM Zauhar RJ. Computational analysis of EBNA1 “druggability” suggests novel insights for Epstein-Barr virus inhibitor design. J Comput Aided Mol Des (2016) 30:285–303. doi: 10.1007/s10822-016-9899-y 27048620 PMC5180362

[353] Kim SY Song KA Kieff E Kang MS. Small molecule and peptide-mediated inhibition of Epstein-Barr virus nuclear antigen 1 dimerization. Biochem Biophys Res Commun (2012) 424:251–6. doi: 10.1016/j.bbrc.2012.06.095 22735264

[354] AlQarni S Al-Sheikh Y Campbell D Drotar M Hannigan A Boyle S. Lymphomas driven by Epstein-Barr virus nuclear antigen-1 (EBNA1) are dependant upon Mdm2. Oncogene (2018) 37:3998–4012. doi: 10.1038/s41388-018-0147-x 29691476 PMC6054874

[355] Cohen JI. Vaccine development for Epstein-Barr virus. Adv Exp Med Biol (2018) 1045:477–93. doi: 10.1007/978-981-10-7230-7_22 PMC6328312 29896681

[356] Bu W Joyce MG Nguyen H Banh DV Aguilar F Tariq Z. Immunization with components of the viral fusion apparatus elicits antibodies that neutralize Epstein-Barr virus in b cells and epithelial cells. Immunity (2019) 50:1305–1316 e6. doi: 10.1016/j.immuni.2019.03.010 30979688 PMC6660903

[357] Sokal EM Hoppenbrouwers K Vandermeulen C Moutschen M Leonard P Moreels A. Recombinant gp350 vaccine for infectious mononucleosis: a phase 2, randomized, double-blind, placebo-controlled trial to evaluate the safety, immunogenicity, and efficacy of an Epstein-Barr virus vaccine in healthy young adults. J Infect Dis (2007) 196:1749–53. doi: 10.1086/523813 18190254

[358] Spriggs MK Armitage RJ Comeau MR Strockbine L Farrah T Macduff B. The extracellular domain of the Epstein-Barr virus BZLF2 protein binds the HLA-DR beta chain and inhibits antigen presentation. J Virol (1996) 70:5557–63. doi: 10.1128/jvi.70.8.5557-5563.1996 PMC190515 8764069

[359] Chesnokova LS Nishimura SL Hutt-Fletcher LM. Fusion of epithelial cells by Epstein-Barr virus proteins is triggered by binding of viral glycoproteins gHgL to integrins alphavbeta6 or alphavbeta8. Proc Natl Acad Sci U.S.A. (2009) 106:20464–9. doi: 10.1073/pnas.0907508106 PMC2787161 19920174

[360] Chen J Sathiyamoorthy K Zhang X Schaller S Perez White BE Jardetzky TS. Ephrin receptor A2 is a functional entry receptor for Epstein-Barr virus. Nat Microbiol (2018) 3:172–80. doi: 10.1038/s41564-017-0081-7 PMC5972547 29292384

[361] Brooks JM Long HM Tierney RJ Shannon-Lowe C Leese AM Fitzpatrick M. Early T cell recognition of b cells following Epstein-Barr virus infection: Identifying potential targets for prophylactic vaccination. PloS Pathog (2016) 12:e1005549. doi: 10.1371/journal.ppat.1005549 27096949 PMC4838210

[362] Adhikary D Behrends U Moosmann A Witter K Bornkamm GW Mautner J. Control of Epstein-Barr virus infection *in vitro* by T helper cells specific for virion glycoproteins. J Exp Med (2006) 203:995–1006. doi: 10.1084/jem.20051287 16549597 PMC2118290

[363] Ura T Okuda K Shimada M. Developments in viral vector-based vaccines. Vaccines (Basel) (2014) 2:624–41. doi: 10.3390/vaccines2030624 PMC4494222 26344749

[364] Gu SY Huang TM Ruan L Miao YH Lu H Chu CM. First EBV vaccine trial in humans using recombinant vaccinia virus expressing the major membrane antigen. Dev Biol Stand (1995) 84:171–7. 7796951

[365] Taylor GS Haigh TA Gudgeon NH Phelps RJ Lee SP Steven NM. Dual stimulation of Epstein-Barr virus (EBV)-specific CD4+- and CD8+-t-cell responses by a chimeric antigen construct: potential therapeutic vaccine for EBV-positive nasopharyngeal carcinoma. J Virol (2004) 78:768–78. doi: 10.1128/JVI.78.2.768-778.2004 PMC368843 14694109

[366] Taylor GS Jia H Harrington K Lee LW Turner J Ladell K. A recombinant modified vaccinia ankara vaccine encoding Epstein-Barr virus (EBV) target antigens: a phase I trial in UK patients with EBV-positive cancer. Clin Cancer Res (2014) 20:5009–22. doi: 10.1158/1078-0432.CCR-14-1122-T PMC4340506 25124688

[367] Smith C Tsang J Beagley L Chua D Lee V Li V. Effective treatment of metastatic forms of Epstein-Barr virus-associated nasopharyngeal carcinoma with a novel adenovirus-based adoptive immunotherapy. Cancer Res (2012) 72:1116–25. doi: 10.1158/0008-5472.CAN-11-3399 22282657

[368] Ruhl J Leung CS Munz C. Vaccination against the Epstein-Barr virus. Cell Mol Life Sci (2020) 77:4315–24. doi: 10.1007/s00018-020-03538-3 PMC7223886 32367191

[369] Kanekiyo M Bu W Joyce MG Meng G Whittle JR Baxa U. Rational design of an Epstein-Barr virus vaccine targeting the receptor-binding site. Cell (2015) 162:1090–100. doi: 10.1016/j.cell.2015.07.043 PMC4757492 26279189

[370] Roldao A Mellado MC Castilho LR Carrondo MJ Alves PM. Virus-like particles in vaccine development. Expert Rev Vaccines (2010) 9:1149–76. doi: 10.1586/erv.10.115 20923267

[371] Bottcher JP Reis e Sousa C. The role of type 1 conventional dendritic cells in cancer immunity. Trends Cancer (2018) 4:784–92. doi: 10.1016/j.trecan.2018.09.001 PMC6207145 30352680

[372] Tornesello AL Tagliamonte M Buonaguro FM Tornesello ML Buonaguro L. Virus-like particles as preventive and therapeutic cancer vaccines. Vaccines (Basel) (2022) 10(2):227. doi: 10.3390/vaccines10020227 35214685 PMC8879290

[373] Ogembo JG Muraswki MR McGinnes LW Parcharidou A Sutiwisesak R Tison T. A chimeric EBV gp350/220-based VLP replicates the virion b-cell attachment mechanism and elicits long-lasting neutralizing antibodies in mice. J Transl Med (2015) 13:50. doi: 10.1186/s12967-015-0415-2 25885535 PMC4328182

[374] Perez EM Foley J Tison T Silva R Ogembo JG. Novel Epstein-Barr virus-like particles incorporating gH/gL-EBNA1 or gB-LMP2 induce high neutralizing antibody titers and EBV-specific T-cell responses in immunized mice. Oncotarget (2017) 8:19255–73. doi: 10.18632/oncotarget.13770 PMC5386682 27926486

[375] Delecluse HJ Pich D Hilsendegen T Baum C Hammerschmidt W. A first-generation packaging cell line for Epstein-Barr virus-derived vectors. Proc Natl Acad Sci U.S.A. (1999) 96:5188–93. doi: 10.1073/pnas.96.9.5188 PMC21839 10220441

[376] Feederle R Shannon-Lowe C Baldwin G Delecluse HJ. Defective infectious particles and rare packaged genomes produced by cells carrying terminal-repeat-negative epstein-barr virus. J Virol (2005) 79:7641–7. doi: 10.1128/JVI.79.12.7641-7647.2005 PMC1143645 15919916

[377] Ruiss R Jochum S Wanner G Reisbach G Hammerschmidt W Zeidler R. A virus-like particle-based Epstein-Barr virus vaccine. J Virol (2011) 85:13105–13. doi: 10.1128/JVI.05598-11 PMC3233152 21994444

[378] Pavlova S Feederle R Gartner K Fuchs W Granzow H Delecluse HJ. An Epstein-Barr virus mutant produces immunogenic defective particles devoid of viral DNA. J Virol (2013) 87:2011–22. doi: 10.1128/JVI.02533-12 PMC3571473 23236073

[379] van Zyl DG Tsai MH Shumilov A Schneidt V Poirey R Schlehe B. Immunogenic particles with a broad antigenic spectrum stimulate cytolytic T cells and offer increased protection against EBV infection ex vivo and in mice. PloS Pathog (2018) 14:e1007464. doi: 10.1371/journal.ppat.1007464 30521644 PMC6298685

[380] Ehlers B Spiess K Leendertz F Peeters M Boesch C Gatherer D. Lymphocryptovirus phylogeny and the origins of Epstein-Barr virus. J Gen Virol (2010) 91:630–42. doi: 10.1099/vir.0.017251-0 19923263

[381] Cohen JI Fauci AS Varmus H Nabel GJ. Epstein-Barr Virus: an important vaccine target for cancer prevention. Sci Transl Med (2011) 3:107fs7. doi: 10.1126/scitranslmed.3002878 PMC3501269 22049067

[382] Huang S Yasuda T. Pathologically relevant mouse models for Epstein-Barr virus-associated b cell lymphoma. Front Immunol (2021) 12:639844. doi: 10.3389/fimmu.2021.639844 33732260 PMC7959712

[383] Zhang B Kracker S Yasuda T Casola S Vanneman M Homig-Holzel C. Immune surveillance and therapy of lymphomas driven by Epstein-Barr virus protein LMP1 in a mouse model. Cell (2012) 148:739–51. doi: 10.1016/j.cell.2011.12.031 PMC3313622 22341446

[384] Felix NJ Allen PM. Specificity of T-cell alloreactivity. Nat Rev Immunol (2007) 7:942–53. doi: 10.1038/nri2200 18007679

[385] Leung C Chijioke O Gujer C Chatterjee B Antsiferova O Landtwing V. Infectious diseases in humanized mice. Eur J Immunol (2013) 43:2246–54. doi: 10.1002/eji.201343815 23913412

[386] Munz C. EBV infection of mice with reconstituted human immune system components. Curr Top Microbiol Immunol (2015) 391:407–23. doi: 10.1007/978-3-319-22834-1_14 26428383

[387] Munz C. Humanized mouse models for Epstein Barr virus infection. Curr Opin Virol (2017) 25:113–8. doi: 10.1016/j.coviro.2017.07.026 28837889

[388] Wang F. Nonhuman primate models for Epstein-Barr virus infection. Curr Opin Virol (2013) 3:233–7. doi: 10.1016/j.coviro.2013.03.003 PMC3713193 23562212

[389] Peng RJ Han BW Cai QQ Zuo XY Xia T Chen JR. Genomic and transcriptomic landscapes of Epstein-Barr virus in extranodal natural killer T-cell lymphoma. Leukemia (2019) 33:1451–62. doi: 10.1038/s41375-018-0324-5 PMC6756073 30546078

[390] Arvey A Tempera I Tsai K Chen HS Tikhmyanova N Klichinsky M. An atlas of the Epstein-Barr virus transcriptome and epigenome reveals host-virus regulatory interactions. Cell Host Microbe (2012) 12:233–45. doi: 10.1016/j.chom.2012.06.008 PMC3424516 22901543

[391] Tang KW Alaei-Mahabadi B Samuelsson T Lindh M Larsson E. The landscape of viral expression and host gene fusion and adaptation in human cancer. Nat Commun (2013) 4:2513. doi: 10.1038/ncomms3513 24085110 PMC3806554

[392] Khoury JD Tannir NM Williams MD Chen Y Yao H Zhang J. Landscape of DNA virus associations across human malignant cancers: analysis of 3,775 cases using RNA-seq. J Virol (2013) 87:8916–26. doi: 10.1128/JVI.00340-13 PMC3754044 23740984

[393] Liu Y Huang H. Genome wide profiling of Epstein-Barr virus (EBV) isolated from EV-related malignancies. Epstein-Barr Virus - New Trends IntechOpen (2020).

[394] Kwok H Chiang AK. From conventional to next generation sequencing of Epstein-Barr virus genomes. Viruses (2016) 8:60. doi: 10.3390/v8030060 26927157 PMC4810250

[395] Xu M Zhang WL Zhu Q Zhang S Yao YY Xiang T. Genome-wide profiling of Epstein-Barr virus integration by targeted sequencing in Epstein-Barr virus associated malignancies. Theranostics (2019) 9:1115–24. doi: 10.7150/thno.29622 PMC6401403 30867819

[396] Xu M Yao Y Chen H Zhang S Cao SM Zhang Z. Genome sequencing analysis identifies Epstein-Barr virus subtypes associated with high risk of nasopharyngeal carcinoma. Nat Genet (2019) 51:1131–6. doi: 10.1038/s41588-019-0436-5 PMC6610787 31209392

[397] Mani SKK Yan B Cui Z Sun J Utturkar S Foca A. Restoration of RNA helicase DDX5 suppresses hepatitis b virus (HBV) biosynthesis and wnt signaling in HBV-related hepatocellular carcinoma. Theranostics (2020) 10:10957–72. doi: 10.7150/thno.49629 PMC7532671 33042264

[398] Rahman N Sun J Li Z Pattnaik A Mohallem R Wang M. The cytoplasmic LSm1-7 and nuclear LSm2-8 complexes exert opposite effects on hepatitis b virus biosynthesis and interferon responses. Front Immunol (2022) 13:970130. doi: 10.3389/fimmu.2022.970130 36016928 PMC9396650

[399] Kazemian M Ren M Lin JX Liao W Spolski R Leonard WJ. Comprehensive assembly of novel transcripts from unmapped human RNA-seq data and their association with cancer. Mol Syst Biol (2015) 11:826. doi: 10.15252/msb.156172 26253570 PMC4562499

[400] Bayda N Tilloy V Chaunavel A Bahri R Halabi MA Feuillard J. Comprehensive Epstein-Barr virus transcriptome by RNA-sequencing in angioimmunoblastic T cell lymphoma (AITL) and other lymphomas. Cancers (Basel) (2021) 13(4):610. doi: 10.3390/cancers13040610 33557089 PMC7913808

[401] Wang C Li D Zhang L Jiang S Liang J Narita Y. RNA Sequencing analyses of gene expression during Epstein-Barr virus infection of primary b lymphocytes. J Virol (2019) 93:e00226–19. doi: 10.1128/JVI.00226-19 31019051 PMC6580941

[402] Aran D Hu Z Butte AJ. xCell: digitally portraying the tissue cellular heterogeneity landscape. Genome Biol (2017) 18:220. doi: 10.1186/s13059-017-1349-1 29141660 PMC5688663

[403] Racle J Gfeller D. EPIC: A tool to estimate the proportions of different cell types from bulk gene expression data. Methods Mol Biol (2020) 2120:233–48. doi: 10.1007/978-1-0716-0327-7_17 32124324

[404] Menden K Marouf M Oller S Dalmia A Magruder DS Kloiber K. Deep learning-based cell composition analysis from tissue expression profiles. Sci Adv (2020) 6:eaba2619. doi: 10.1126/sciadv.aba2619 32832661 PMC7439569

[405] Tang F Barbacioru C Wang Y Nordman E Lee C Xu N. mRNA-seq whole-transcriptome analysis of a single cell. Nat Methods (2009) 6:377–82. doi: 10.1038/nmeth.1315 19349980

[406] Chauss D Freiwald T McGregor R Yan B Wang L Nova-Lamperti E. Autocrine vitamin d signaling switches off pro-inflammatory programs of TH1 cells. Nat Immunol (2022) 23:62–74. doi: 10.1038/s41590-021-01080-3 34764490 PMC7612139

[407] Yan B Freiwald T Chauss D Wang L West E Mirabelli C. SARS-CoV-2 drives JAK1/2-dependent local complement hyperactivation. Sci Immunol (2021) 6(58). doi: 10.1126/sciimmunol.abg0833 PMC8139422 33827897

[408] Jin S Li R Chen MY Yu C Tang LQ Liu YM. Single-cell transcriptomic analysis defines the interplay between tumor cells, viral infection, and the microenvironment in nasopharyngeal carcinoma. Cell Res (2020) 30:950–65. doi: 10.1038/s41422-020-00402-8 PMC7784966 32901110

[409] SoRelle ED Dai J Reinoso-Vizcaino NM Barry AP Chan C Luftig MA. Time-resolved transcriptomes reveal diverse b cell fate trajectories in the early response to Epstein-Barr virus infection. Cell Rep (2022) 40:111286. doi: 10.1016/j.celrep.2022.111286 36044865 PMC9879279

[410] Harley JB Chen X Pujato M Miller D Maddox A Forney C. Transcription factors operate across disease loci, with EBNA2 implicated in autoimmunity. Nat Genet (2018) 50:699–707. doi: 10.1038/s41588-018-0102-3 29662164 PMC6022759

[411] Leong MML Lung ML. The impact of Epstein-Barr virus infection on epigenetic regulation of host cell gene expression in epithelial and lymphocytic malignancies. Front Oncol (2021) 11:629780. doi: 10.3389/fonc.2021.629780 33718209 PMC7947917

[412] Okabe A Funata S Matsusaka K Namba H Fukuyo M Rahmutulla B. Regulation of tumour related genes by dynamic epigenetic alteration at enhancer regions in gastric epithelial cells infected by Epstein-Barr virus. Sci Rep (2017) 7:7924. doi: 10.1038/s41598-017-08370-7 28801683 PMC5554293

[413] Wang L Laing J Yan B Zhou H Ke L Wang C. Epstein-Barr Virus episome physically interacts with active regions of the host genome in lymphoblastoid cells. J Virol (2020) 94(24):e01390–20. doi: 10.1128/JVI.01390-20 32999023 PMC7925191

[414] Zhao W Mo Y Wang S Midorikawa K Ma N Hiraku Y. Quantitation of DNA methylation in Epstein-Barr virus-associated nasopharyngeal carcinoma by bisulfite amplicon sequencing. BMC Cancer (2017) 17:489. doi: 10.1186/s12885-017-3482-3 28716111 PMC5514474

[415] Jiang S Zhou H Liang J Gerdt C Wang C Ke L. The Epstein-Barr virus regulome in lymphoblastoid cells. Cell Host Microbe (2017) 22:561–573 e4. doi: 10.1016/j.chom.2017.09.001 29024646 PMC5662195

[416] Wen Y Xu H Han J Jin R Chen H. How does Epstein-Barr virus interact with other microbiomes in EBV-driven cancers? Front Cell Infect Microbiol (2022) 12:852066. doi: 10.3389/fcimb.2022.852066 35281433 PMC8904896

